# Combining SIMS and mechanistic modelling to reveal nutrient kinetics in an algal-bacterial mutualism

**DOI:** 10.1371/journal.pone.0251643

**Published:** 2021-05-20

**Authors:** Hannah Laeverenz Schlogelhofer, François J. Peaudecerf, Freddy Bunbury, Martin J. Whitehouse, Rachel A. Foster, Alison G. Smith, Ottavio A. Croze

**Affiliations:** 1 Cavendish Laboratory, University of Cambridge, Cambridge, United Kingdom; 2 Department of Applied Mathematics and Theoretical Physics, University of Cambridge, Cambridge, United Kingdom; 3 Department of Plant Sciences, University of Cambridge, Cambridge, United Kingdom; 4 Swedish Museum of Natural History, Stockholm, Sweden; 5 Department of Ecology, Environment and Plant Sciences, Stockholm University, Stockholm, Sweden; University of Houston, UNITED STATES

## Abstract

Microbial communities are of considerable significance for biogeochemical processes, for the health of both animals and plants, and for biotechnological purposes. A key feature of microbial interactions is the exchange of nutrients between cells. Isotope labelling followed by analysis with secondary ion mass spectrometry (SIMS) can identify nutrient fluxes and heterogeneity of substrate utilisation on a single cell level. Here we present a novel approach that combines SIMS experiments with mechanistic modelling to reveal otherwise inaccessible nutrient kinetics. The method is applied to study the onset of a synthetic mutualistic partnership between a vitamin B_12_-dependent mutant of the alga *Chlamydomonas reinhardtii* and the B_12_-producing, heterotrophic bacterium *Mesorhizobium japonicum*, which is supported by algal photosynthesis. Results suggest that an initial pool of fixed carbon delays the onset of mutualistic cross-feeding; significantly, our approach allows the first quantification of this expected delay. Our method is widely applicable to other microbial systems, and will contribute to furthering a mechanistic understanding of microbial interactions.

## Introduction

Microbial communities underpin many globally important processes, from biogeochemical cycles [1] and the ecology of aquatic [2] and terrestrial food webs [3,4], to wastewater treatment [5,6] and the health of agricultural soils [7]. A key feature of the interactions within these communities is the exchange of metabolites between species [8]. In aquatic environments, photosynthetic carbon fixation by phytoplankton supports higher trophic levels, but also provides an important carbon source for heterotrophic bacteria [9,10]. Conversely, bacteria have been shown to provide limiting nutrients to algae, including nitrates, phosphates and iron [11], vitamins [12,13] and carbon dioxide [14]. Depending on environmental conditions, these metabolite exchanges control the outcome of microbial interactions, from parasitic, through commensal, to mutualistic [15,16].

To exploit microbial communities for biotechnological applications, it is crucial to be able to predict and control microbial interactions. Extensive studies of natural microbial communities using metagenomics, metatranscriptomics and metaproteomics have provided considerable insight into potential metabolite exchanges [17,18]. However, to obtain a fully predictive, mechanistic understanding of microbial interactions it is also essential to use bottom-up approaches employing laboratory model systems and mathematical models [19,20]. For example, the comparison of a nutrient-implicit Lotka-Volterra model with experiments studying co-cultures of genetically engineered strains of yeast that each provide a different essential nutrient to the other demonstrated a limiting nutrient-induced shift from mutualism via parasitism to competition [21]. Moreover, studies of engineered yeast communities combining agar pad experiments and models incorporating nutrient diffusion revealed that cross-feeding interactions influence genetic drift during spatial expansion [22], and that spatial self-organisation favours cooperation over cheating [23].

The exact metabolic interactions within microbial communities are often unknown. Secondary ion mass spectrometry (SIMS, NanoSIMS), an imaging mass spectrometry technique capable of analysing single microbial cells, reviewed in [24–27], has been instrumental in identifying new symbioses and microbial interactions for both cultured and non-cultured associations [28–30]. Moreover, the metabolic activity and phylogenetic identity (16S rRNA) of single cells can be linked by combining *in situ* hybridization methods with SIMS [31,32]. Using SIMS and NanoSIMS to visualise and quantify substrate utilisation in single cells, filaments, and colonies of microbial cells has helped to determine the heterogeneity of single cell metabolic activity [31,33], sub-cellular location of assimilated substrates [34,35], nutrient exchanges between symbiotic partners [28,29] and the effect of physical attachment on carbon and nitrogen fluxes between bacteria and microalgae [36,37].

In these studies, SIMS was primarily used to visualise and measure nutrient assimilation and transfer. In the dilute aquatic environment, microbial interactions will involve dynamic nutrient exchanges, particularly at the onset of association, when metabolite fluxes may be quite different from those arising during a stable, long-term interaction. Here we explore the establishment of mutualistic interactions with a well-characterised model system: a co-culture of the cobalamin (vitamin B_12_) dependent, photosynthetic alga *Chlamydomonas reinhardtii* metE7 strain [38] and the B_12_-producing, heterotrophic bacterium *Mesorhizobium japonicum* (previously *Mesorhizobium loti* [39]). This laboratory model system has environmental relevance because omics studies have shown that vitamin-mediated mutualistic interactions between microalgae and bacteria are widespread in terrestrial and marine environments [18]. Further, the system has been extensively studied in the laboratory. Previous studies of a closely related system comprising the naturally B_12_-dependent alga *Lobomonas rostrata*, have demonstrated mutualistic growth dynamics predicated on the exchange of vitamin B_12_ and organic carbon photosynthate [38,40]. The relative proportions of the two organisms are stably maintained over hundreds of generations, but can be perturbed by supplementation with cobalamin or an organic carbon source like glycerol [40]. The effect of environment geometry on the mutualistic dynamics of spatially separated populations was also recently modelled mathematically, and realised experimentally [41]. Here, SIMS experiments that follow the temporal variation in ^13^*C* labelling are combined with a mechanistic, nutrient-explicit model to gain further insight into how these organisms interact. In principle, isotope labelling and SIMS alone could quantify all nutrient exchanges of interest. In practice, however, this would require a myriad of time-consuming experiments in order to separate the coupled dynamics. The value of our mechanistic model is to allow information about the nutrient kinetics to be deduced from a minimal, and feasible to perform, set of experiments. Further, the model helps suggest which metabolic processes are most significantly contributing to observations, permits use of the SIMS data to explore potential mechanisms for the observed single cell heterogeneity and, in future, could help identify trends in how metabolic processes and nutrient dynamics change under different environmental conditions.

### Mechanistic model

**Fig 1:**
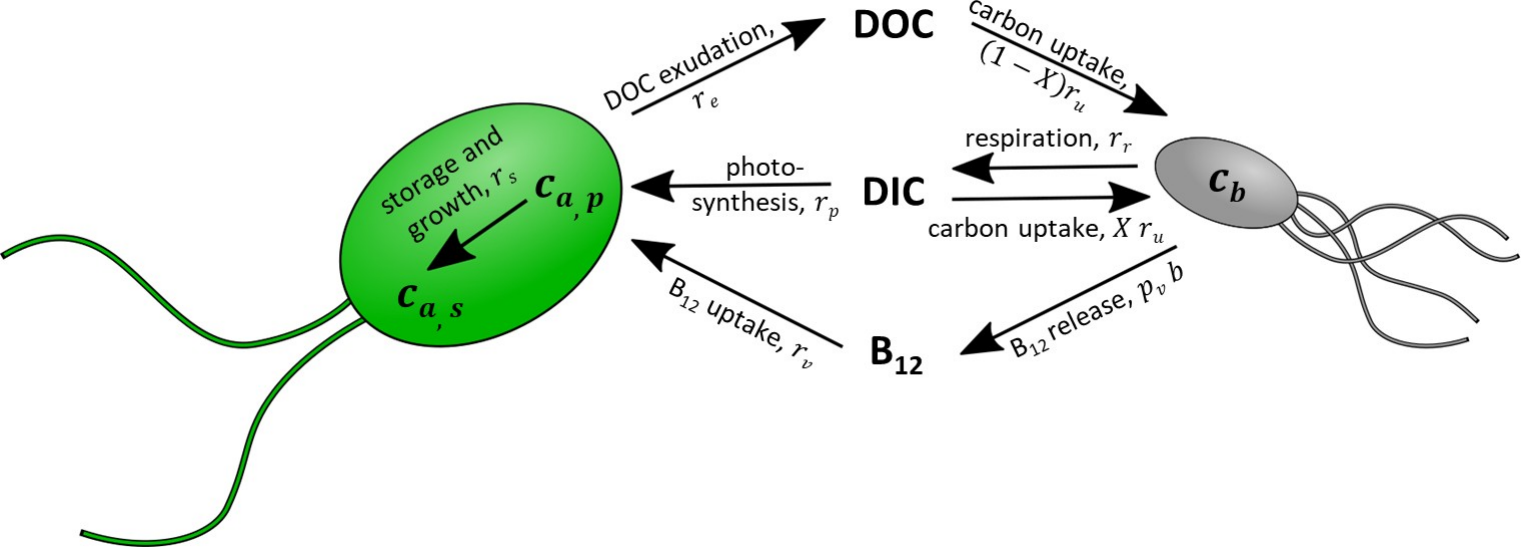
Schematic to illustrate the nutrient kinetics included in the algal-bacterial co-culture model. Vitamin B_12_ is released by bacteria and required for algal growth. Bacterial growth is dependent on DOC produced by algae. Also considered are: algal photosynthesis, carbon storage, and DOC exudation from excess photosynthesis; bacterial respiration and DIC uptake.

To better understand the carbon kinetics revealed by isotope labelling experiments and the underlying mutualistic microbial dynamics of the algal-bacterial co-culture, a mechanistic model was formulated. The nutrient-explicit model (shown schematically in Fig 1) does not rely on detailed metabolic fluxes but nevertheless aims to capture the essential nutrient exchanges between the algae and bacteria. Algal population growth depends on the external concentration of B_12_ *v*, which originates from bacterial release. Similarly, bacterial population growth depends on the external concentration of algal-derived dissolved organic carbon (DOC), modelled as an effective single carbon source *c*_*o*_. All other nutrients are assumed to be non-limiting. The exchange of B_12_ and DOC thus provides mutualistic coupling between the two species. The nutrient-limited growth is modelled using a Monod model, and a logistic term is added to model the long-term growth of limited batch cultures [42].

Finally, the co-culture is assumed to be well-mixed, such that

$$\frac{da}{dt}=\mu_{a}\;a\left(1-\frac{a}{K_{a}}\right)\left(\frac{v}{K_{v}+v}\right)$$ (1)

$$\mathrm{and}\;\frac{db}{dt}=\mu_{b}\;b\left(1-\frac{b}{K_{b}}\right)(\frac{c_{o}}{K_{c}+c_{o}}),$$ (2)

with *a* and *b* the algal and bacterial cell densities respectively, *μ*_*a*_ and *μ*_*b*_ the maximum growth rates (defined here with units *h*^−1^), *K*_*a*_ and *K*_*b*_ the carrying capacities (here with units *cells*/*mL*), and *K*_*v*_ and *K*_*c*_ the half-saturation concentrations (here with units *mol*/*mL*). The carbon biomass concentrations for algae and bacteria are given by

$$c_{a}=\frac{a}{Y_{a,c}}$$ (3)

$${and\;c}_{b}=\frac{b}{Y_{b,c}}$$ (4)

respectively, with *Y*_*a*,*c*_ and *Y*_*b*,*c*_ the carbon yield parameters, which are defined as the number of cells formed per mole of carbon (i.e. with units *cells*/*molC*) and assumed to be constant.

Although dissolved inorganic carbon (DIC) is assumed to be non-limiting (as in the experiments), accounting for DIC kinetics was essential to connect the model to SIMS experiments, where isotope labelling relied on assimilation of ^13^*C* via DIC. Heterotrophic bacteria must respire to produce the ATP required to drive cellular metabolism [43], therefore a significant fraction of the carbon consumed by bacteria will be transformed into carbon dioxide through respiration. This is modelled using the bacterial growth efficiency (BGE), defined as

$$\eta^{\prime }=\frac{{\dot{c}}_{b}}{{\dot{c}}_{b}+r_{r}},$$ (5)

with ${\dot{c}}_{b}$ the bacterial carbon biomass growth rate and *r*_*r*_ the respiration rate. In order for the carbon fluxes to remain balanced, while also maintaining an active carbon turnover at carrying capacity, *η*′ decreases to zero as the bacterial cell density increases to carrying capacity. Therefore

$$\eta^{\prime }=\eta (1-\frac{b}{K_{b}}),$$ (6)

with *η* the maximum BGE, i.e. the growth efficiency for the exponential growth phase when *b*≪*K*_*b*_. For *η*→1, respiration goes to zero and does not affect carbon uptake. Instead, with *η*→0 respiration rate is high compared to growth rate and thus strongly affects the carbon kinetics. The total rate of carbon uptake by bacteria is given by $r_{u}={\dot{c}}_{b}+r_{r}$ and so from Eqs (2) and (4)-(6)

$$r_{u}=\frac{\mu_{b}\;b}{\eta \;Y_{b,c}}\left(\frac{c_{o}}{K_{c}+c_{o}}\right)$$ (7)

and the total bacterial respiration rate, defined as DIC production, is therefore given by

$$r_{r}=\left(1-\eta (1-\frac{b}{K_{b}})\right)r_{u}.$$ (8)

As any living cell, heterotrophic bacteria can assimilate inorganic carbon through carboxylation reactions [44,45]. The model incorporates this observation by including a DIC uptake parameter *X*, defined as

$$X=\frac{r_{u}^{DIC}}{r_{u}},$$ (9)

with $r_{u}^{DIC}$ the DIC uptake rate and *r*_*u*_ the total carbon uptake rate.

Further, the model minimally describes photosynthesis and carbon storage in algae by splitting algal carbon biomass into two internal components, *photosynthetically-active* carbon *c*_*a*,*p*_, available for exudation, and *stored* carbon *c*_*a*,*s*_, used for biomass growth, in storage compounds (e.g. starch) and for cellular maintenance. We define the fraction of carbon ‘*stored’* by algae as

$$\phi_{s}=\frac{c_{a,s}}{c_{a}},$$ (10)

from which a *rate of storage* can be defined as

$$r_{s}={\dot{c}}_{a,s}=\frac{\phi_{s}\dot{a}}{Y_{a,c}}.$$ (11)

For simplicity, the model does not consider the carbon concentrating mechanism of *C*. *reinhardtii* explicitly. Instead carbon dioxide and bicarbonate are considered as one entity (i.e. DIC) and photosynthetic assimilation of DIC corresponds to the uptake of both forms of inorganic carbon. Total carbon must be conserved and therefore the rate of photosynthetic carbon assimilation is given by

$$r_{p}=\frac{\dot{a}}{Y_{a,c}}+r_{e},$$ (12)

with $\dot{a}/Y_{a,c}$ the algal carbon biomass growth rate and *r*_*e*_ the total rate of DOC exudation by the whole algal population, which is assumed to be linearly dependent on the algal cell density and the fraction of stored carbon, such that

$$r_{e}=(1-\phi_{s})p_{c}\;a.$$ (13)

The parameter *p*_*c*_ is assumed to be constant and can be interpreted as a measure of the rate of DOC release by algae per unit of *photosynthetically-active* algal biomass. Thus, the model effectively describes DOC exudation as originating from excess algal photosynthesis. Taking all these different contributions to the carbon kinetics into account, the model defines the rate of change of the DOC and DIC concentrations as

$$\frac{dc_{o}}{dt}=r_{e}-(1-X)r_{u}$$ (14)

$$\mathrm{and}\;\frac{dc_{i}}{dt}=r_{r}-X\;r_{u}-r_{p},$$ (15)

respectively.

For the B_12_ kinetics, the internal B_12_ recycling dynamics for algae and bacteria are neglected, including the reacquisition of released vitamins by the bacteria. The experimental details of the vitamin import/export dynamics have yet to be investigated for our system, so it is not possible to describe it as has been done for some bacterial systems [46]. Here, the total B_12_ uptake rate for the whole algal population is given by

$$r_{v}=\frac{\mu_{a}\;a}{Y_{a,v}}(\frac{v}{K_{v}+v}),$$ (16)

with *Y*_*a*,*v*_ the B_12_ yield for algae in the exponential growth phase, i.e. *a*≪*K*_*a*_, and has units *cells*/*molB*_12_. It is assumed that there is a constant B_12_ release rate per bacterial cell *p*_*v*_, meaning that

$$\frac{dv}{dt}=p_{v}b-r_{v}.$$ (17)

Combining the differential equations for the carbon concentrations and the definition of atomic fraction *f* = ^13^*C*/(^13^*C*+^12^*C*), we can write down differential equations for the dynamics of the atomic fractions, observed experimentally using SIMS. Full details are given in Supplementary Methods in S1 Text, as an example, the atomic fraction for bacteria can be shown to be given by

$$\frac{df_{b}}{dt}=(X\;f_{i}+(1-X)f_{o}-f_{b})\frac{\mu_{b}}{\eta }(\frac{c_{o}}{K_{c}+c_{o}}),$$ (18)

with *f*_*b*_, *f*_*i*_ and *f*_*o*_ the atomic fractions of ^13^*C* for bacteria, DIC and DOC respectively, and all other parameters as previously defined.

In summary, the mechanistic model describes algal growth dependent on B_12_ produced by bacteria, with photosynthetic uptake of DIC accounting for the algal carbon biomass growth and DOC exudation. The bacterial growth is dependent on the DOC produced by algae, respiration produces carbon dioxide (DIC) and provides the bacteria with the energy they require to grow. In addition to DOC uptake, bacteria are also able to assimilate DIC through metabolic carboxylation reactions.

### Model parameters

A brief overview of the model parameterisation is provided here; full details can be found in the Supplementary Methods in S1 Text. Model parameters were estimated by fitting the model to the experimental results for both growth and isotope fraction simultaneously. The Matlab ordinary differential equation solver *ode45* was used to numerically solve the model equations. To reduce the number of free parameters, the majority of model parameters were determined using a simplified version of the co-culture model (i.e. with *ϕ*_*s*_ = 0, *η*′ = 1 and *X* = 0) to run a global fit of three independent co-cultures (i.e. not the same as the stable isotope experiments in this work) between *C*. *reinhardtii* metE7 and *M*. *japonicum*, for which colony forming units, particle counts and B_12_ concentrations were measured. The algal and bacterial carbon yields were estimated from dry mass measurements and EA-IRMS analysis. The carrying capacity for axenic bacteria was obtained from fitting a logistic growth equation to data obtained by [40] for *M*. *japonicum* grown axenically with 0.1% glycerol. The few remaining parameters were obtained from fitting the model to the main stable isotope experiments in this work, see Results. Global parameter optimisations were performed using the *GlobalSearch* and *createOptimProblem* functions in Matlab’s global optimisation toolbox, with *fmincon* as the solver for each minimisation. The results for the model parameters and initial conditions for *C*. *reinhardtii* metE7 and *M*. *japonicum* grown both axenically and in co-culture are given in Table 1. Although the individual parameters can have different values, it was assumed that the underlying mechanisms of the model are the same for axenic and co-culture conditions. In particular, it was assumed that the carbon metabolism of bacteria will behave in the same way when they are grown axenically or in co-culture: growth, carbon assimilation and respiration rate will all be correlated in the same way for both conditions.

**Table 1:** Model parameters and initial conditions.

**Parameter**	**Symbol**	**Units**	**Axenic algae**	**Axenic bacteria**	**Co-culture**
Algal carrying capacity	*K*_*a*_	*cells mL*^−1^	2.3×10^6^ [a]	-	2.3×10^6^ [a]
Bacterial carrying capacity	*K*_*b*_	*cells mL*^−1^	-	1.3×10^9^ [b]	1.14×10^9^ [a]
B_12_ half-saturation concentration	*K*_*v*_	*mol mL*^−1^	2.6×10^−14^ [a]	-	2.6×10^−14^[a]
DOC half-saturation concentration	*K*_*c*_	*mol mL*^−1^	-	1.5×10^−6^ [c]	6.3×10^−7^ [d]
Maximum bacterial growth rate	*μ*_*b*_	*h*^−1^	-	0.15 [c]	0.42 [a]
Maximum algal growth rate	*μ*_*a*_	*h*^−1^	0.21 [a]	-	0.21 [a]
Algal B_12_ yield	*Y*_*a*,*v*_	*cells mol*^−1^	1.13×10^19^ [e]	-	1.13×10^19^ [e]
Algal carbon yield	*Y*_*a*,*c*_	*cells mol*^−1^	4×10^12^ [f]	-	4×10^12^ [f]
Bacterial carbon yield	*Y*_*b*,*c*_	*cells mol*^−1^	-	5×10^14^ [f]	5×10^14^ [f]
B_12_ release rate	*p*_*v*_	*mol cell*^−1^ *h*^−1^	-	-	2×10^−23^ [g]
DOC production parameter	*p*_*c*_	*mol cell*^−1^ *h*^−1^	-	-	5.4×10^−15^ [h]
Fraction of storage	*ϕ*_*s*_		0.87 [i]	-	0.9 [j]
Maximum BGE	*η*		-	0.51, 0.15, 0.39, 0.63 [^c^,^k^]	0.51 [j]
DIC uptake fraction	*X*		-	0.046, 0.042, 0.022, 0.009 [^c^,^k^]	0.015 [j]
**Initial conditions**	**Symbol**	**Units**	**Axenic algae**	**Axenic bacteria**	**Co-culture**
Algal cell density	*a*(0)	*cells mL*^−1^	0.0032 *K*_*a*_ [i]	0	0.005 *K*_*a*_ [l]
Bacterial cell density	*b*(0)	*cells mL*^−1^	0	8.8×10^6^, 1.6×10^7^, 1.8×10^7^, 1.3×10^7^ [c,k]	0.017 *K*_*b*_ [l]
DOC concentration	*c*_*o*_(0)	*mol mL*^−1^	0	4×10^−5^, 4×10^−6^, 4×10^−7^, 1.7×10^−7^ [c,k]	0.0014 *K*_*c*_ [l]
B_12_ concentration	*v*(0)	*mol mL*^−1^	0.374 *K*_*v*_ [i]	0	0
DIC concentration	*c*_*i*_(0)	*mol mL*^−1^	5 *K*_*c*_	5 *K*_*c*_	5 *K*_*c*_
Algal atomic fraction	*f*_*a*_(0)		0.0108	-	0.59 [m]
Photosynthetically-active atomic fraction	*f*_*a*,*p*_(0)		0.0108	-	0.65 [m]
Bacterial atomic fraction	*f*_*b*_(0)		-	0.0108	0.0108
DOC atomic fraction	*f*_*o*_(0)		0.0108	0.0108	0.64 [m]
DIC atomic fraction	*f*_*i*_(0)		0.65 [i]	0.65 [i]	0.65 [i]

[a] Obtained from fitting a simplified co-culture model (i.e. *ϕ*_*s*_ = 0, *η*′ = 1 and *X* = 0) to population growth and B_12_ concentration data.

[b] From fitting a logistic growth equation to data obtained by [40] for *M*. *japonicum* grown axenically with 0.1% glycerol.

[c] From a global parameter optimisation performed for the four axenic cultures of *M*. *japonicum* grown with different concentrations of glycerol, i.e. the growth and isotope data from all for cultures were fit simultaneously. The residual sum of squares for this global parameter optimisation was 0.58, whereas when respiration was not included in the model it was 2.24.

[d] From the definition $K_{c}=\frac{K_{b}}{Y_{b,c}k_{b,c}}$, with non-dimensional parameter *k*_*b*,*c*_ = 3.6 obtained from [a].

[e] From the definition $Y_{a,v}=\frac{K_{a}}{K_{v}k_{a,v}},$ with non-dimensional parameter *k*_*a*,*v*_ = 7.8 obtained from [a].

[f] From dry mass measurements and EA-IRMS analysis.

[g] From the definition $p_{v}=\frac{s_{v}\mu_{a}K_{v}}{K_{b}}$, with non-dimensional parameter *s*_*v*_ = 4.2 obtained from [a].

[h] From the definition $p_{c}=\frac{s_{c}\mu_{b}K_{c}}{K_{a}}$, with non-dimensional parameter *s*_*c*_ = 0.047 obtained from [l].

[i] Parameter optimisation results from fitting the model to the axenic, pre-labelling culture of *C*. *reinhardtii* metE7. The residual sum of squares for this global parameter optimisation result was 0.313.

[j] Estimated using parameter optimisation results for the axenic cultures.

[k] For the 0.1%, 0.01%, 0.001% and no glycerol cultures respectively. See S7 Table for details.

[l] Parameter optimisation results from fitting the model to co-culture growth and SIMS data, i.e. fit 1 in S8 Table.

[m] Estimated using the model results for the axenic, pre-labelling culture of algae.

The model parameter values obtained from model parameterisations for *C*. *reinhardtii* metE7 and *M*. *japonicum* grown both axenically and in co-culture. See Supplementary Methods in S1 Text for details of the parameterisation methods and S6 Table for details of the non-dimensional model parameters.

## Results

### Inorganic carbon acquisition by axenic bacteria

**Fig 2:**
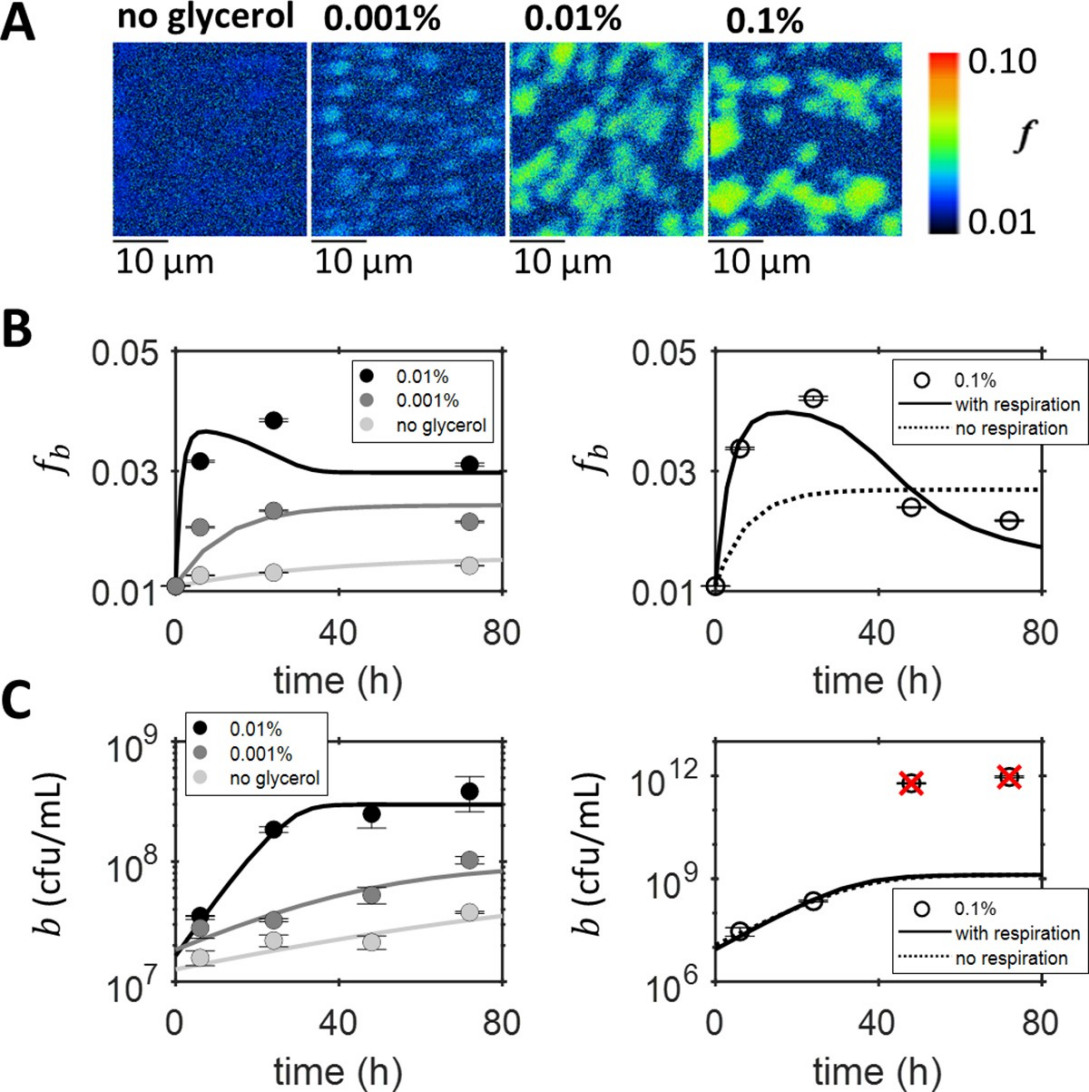
Inorganic carbon acquisition by axenic bacteria. (A) Example images of the atomic fraction of ^13^*C*, *f*, obtained by SIMS analysis of bacterial cells sampled after 24 *h* of axenic cultures grown with different concentrations of unlabelled glycerol and 5 *mM NaH*^13^*CO*_3_. The colour map shows the scale, starting at natural abundance. (B) The mean atomic fraction of ^13^*C*, *f*_*b*_, for dilution-corrected SIMS measurements (for the 0–0.01% glycerol cultures on the left and 0.1% glycerol culture on the right) demonstrate inorganic carbon acquisition by the bacteria. Error bars correspond to standard errors. (C) Bacterial growth measured using viable counts, plotted on a logarithmic scale as the mean of two measurements (with standard error shown as error bars), shows an increase in the exponential growth rate and carrying capacity for higher initial concentration of glycerol (0–0.01% glycerol cultures shown on the left and 0.1% glycerol culture on the right). Red crosses indicate points that were unexpectedly high (approximately 1×10^12^ *cfu mL*^−1^) and therefore considered outliers and not included in the parameter optimisation. The results of fitting the model to both the growth and SIMS data, with parameters and their units as specified in Table 1, are also plotted for the (B) atomic fraction *f*_*b*_ and (C) cell density *b*. For the 0.1% glycerol culture, shown separately on the right, results from two different parameter optimisations are compared. For the fit including respiration (solid line), i.e. with η as a free parameter, the results are given in Table 1. For the fit neglecting respiration (dotted line), i.e. *η*′ = 1, the parameter optimisation results are *K*_*c*_ = 6.6×10^−6^ *molC mL*^−1^, *μ*_*b*_ = 0.15 *h*^−1^ and for the 0.1% glycerol culture *b*(0) = 1.2×10^7^ *cells mL*^−1^ and *X* = 0.025.

Axenic cultures of the rhizobial bacterium *M*. *japonicum* allowed quantification of bacterial inorganic carbon acquisition and provided a benchmark for applying our SIMS-modelling approach to the co-culture. *M*. *japonicum* was grown axenically for 72 *h* with 5 *mM NaH*^13^*CO*_3_ (the labelled DIC source) and different concentrations of unlabelled glycerol, providing a source of organic carbon. SIMS images (Fig 2A) were used to determine the atomic fraction of ^13^*C*, *f*, for individual bacterial cells. The quantity *f*_*b*_ (Fig 2B) represents the average fraction of 13*C* for a distribution of single cell measurements; the effects of single cell heterogeneity are discussed further below.

Concomitantly to SIMS, bacterial abundance was quantified using viable counts. As expected, higher glycerol concentrations resulted in faster exponential growth and larger carrying capacities (Fig 2C). Even with no glycerol added to the growth medium, bacterial growth was still observed (see also S7 Fig). This could be due to internal stored carbon carried forward from the pre-culture or slow growth on other organic carbon sources, e.g. Tris buffer, present in the media. A similar experiment showed that for two days after transfer into axenic culture or co-culture the growth of *M*. *japonicum* remained the same, and only after this did the growth curves diverge when *M*. *japonicum* in axenic culture stopped growing, whereas in co-culture the bacteria transitioned to using algal photosynthate which supported further growth [47]. During the first 24 *h*, when all cultures analysed were in the exponential growth phase, greater ^13^*C*-enrichment was observed for bacteria grown with a higher concentration of glycerol (Fig 2B). Since only inorganic carbon was labelled, the increase in *f*_*b*_ demonstrates DIC acquisition by *M*. *japonicum*.

The co-culture model was applied to interpret the SIMS results for the axenic cultures of *M*. *japonicum*. Mathematically, the model used for axenic bacteria is given by Eqs (2), (14), (15), (17) and (18), with *a* = *v* = *r*_*e*_ = *r*_*p*_ = 0, which describes logistic growth of a bacterial population growing on a limiting organic carbon source.

The model was applied to globally fit both the SIMS and growth data simultaneously. Two such global fits were performed, one including respiration and another ignoring it. In the latter case, the model was unable to reproduce the data well (dotted line in Fig 2B and 2C). This suggests that DIC uptake and respiration are essential to accurately describe the carbon kinetics of axenic bacteria. The bacteria grown with 0.1% glycerol showed a prominent peak in *f*_*b*_, which the model without respiration was unable to reproduce (Fig 2B). This can be explained by considering that only respiration provides the feedback of unlabelled carbon necessary for *f*_*b*_ to decrease. Respiration converts glycerol to *CO*_2_, which is released into solution and lowers the total labelled fraction of DIC. Thus, the labelled fraction of carbon consumed by bacteria decreases, causing *f*_*b*_ to decrease. Fit results for the growth efficiency *η*∈[0.15−0.63] and DIC uptake parameter *X*∈[0.009−0.046] (S7 Table) are similar to those reported in the literature, e.g. *η*∈[0.05−0.6] [48] and *X*∈[0.014−0.065] [45]. Moreover, the DIC uptake parameter *X* was found to increase as a function of the exponential growth rate *μ*_*B*_, according to *X* = *m* ln(*μ*_*B*_)+*n* with *m* = 0.0167±0.0004, *n* = 0.0785±0.0013 and *R*^2^ = 0.999. A negative correlation between the growth efficiency *η* and ln(*μ*_*B*_) was found, giving *η* = *p* ln(*μ*_*B*_)+*q* with *p* = −0.10±0.12, *q* = 0.12±0.36 and *R*^2^ = 0.282 (S7 Table and S8 Fig).

Overall, this study of axenic cultures revealed how the combination of temporal SIMS measurements with modelling can help determine which key metabolic phenomena are responsible for observed isotope labelling dynamics.

### Carbon transfer from algae to bacteria in co-culture

To gain new insights into the establishment of mutualistic algal-bacterial interactions, we applied the combined SIMS-modelling approach to study a co-culture between *C*. *reinhardtii* metE7 and *M*. *japonicum*. The algae were pre-labelled and not washed prior to co-culture inoculation (see Experimental Procedures and S9 Fig), therefore DOC from the pre-culture was carried over into the co-culture. This provided the best chance of observing bacterial assimilation of algal derived carbon, given that the time-scale for DOC to become available to bacteria in the co-culture had not been measured previously.

**Fig 3:**
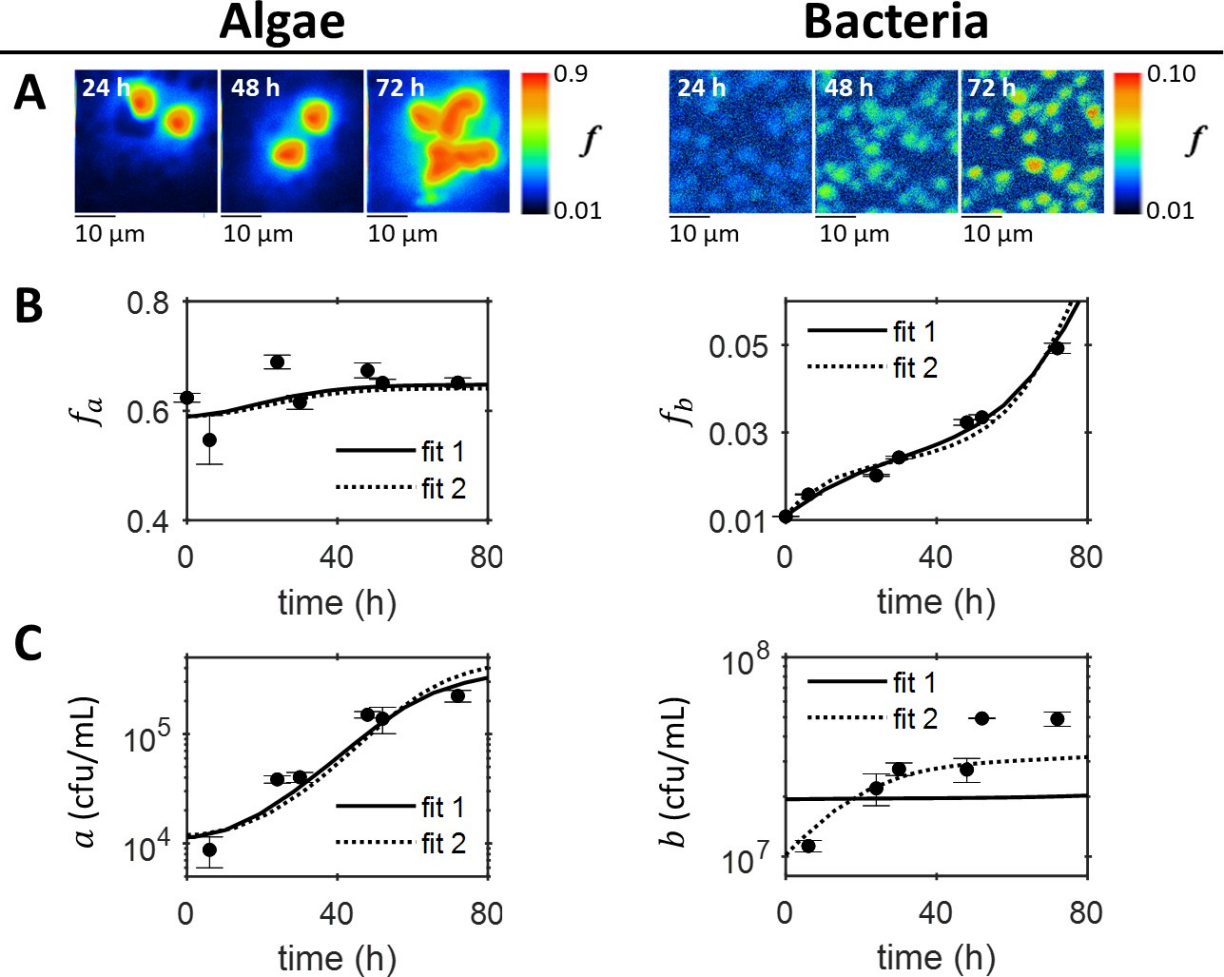
The algal-bacterial co-culture. (A) Example images of the atomic fraction of ^13^*C*, *f*, obtained by SIMS analysis of algal and bacterial cells sampled from the co-culture. The colour maps show the scale, starting at natural abundance. (B) The mean atomic fraction of ^13^*C*, *f*_*a*_ and *f*_*b*_ for algae and bacteria respectively, calculated from the dilution-corrected SIMS measurements for at least 5 algal cells and 100 bacterial cells per time-point (circles). Error bars correspond to standard errors. (C) Algal and bacterial growth measured using viable counts, plotted as the mean (with standard error shown as error bars) of two viable count measurements (circles). The results of fitting the model to both the growth and SIMS data are also plotted. Fit 1 fixed the initial *f*_*o*_(0) = 0.64, estimated using results for the pre-labelling culture of algae, whereas fit 2 included *f*_*o*_(0) as a free parameter. The parameter values and initial conditions are as specified in Table 1 and S8 Table. Although fit 2 fits the data better, it gives a low initial atomic fraction for the DOC *f*_*o*_(0) and high initial DOC concentration *c*_*o*_(0).

The labelled carbon kinetics in the co-culture were followed using SIMS over a period of 72 *h*. SIMS images (Fig 3A) were used to determine the atomic fraction of ^13^*C*, *f*, for individual bacterial and algal cells. The quantities *f*_*a*_ and *f*_*b*_ denote the average atomic fractions for a population of algae and bacteria respectively (Fig 3B); effects relating to single cell heterogeneity are considered below. Sustained population growth was observed for both the algal and bacterial populations (Fig 3C), which implied that the two populations were not limited by nutrients other than the exchanged metabolites. In spite of algal population growth, *f*_*a*_ remained approximately constant throughout the co-culture (Fig 3B), which indicates a likely equilibrium for ^13^*C* in algae, with *f*_*a*_ equal to the atomic fraction of DIC *f*_*i*_ (see Eq (S11) in Supplementary Methods in S1 Text). Unlike for axenic bacteria, where *f*_*b*_ reached a maximum (Fig 2B), for the co-culture *f*_*b*_ continues to increase throughout the experiment (Fig 3B). Bacterial respiration only has a significant effect on the ^13^*C* content of the DIC when the respiration rate is fast enough. The co-cultured bacteria are growing more slowly than when grown with high concentrations of glycerol. Therefore, the total rate of respiration is slower, and hence the feedback of unlabelled CO_2_ is also slower. In addition, it should be noted that in the axenic cultures of bacteria (Fig 2) the only labelled carbon source is the DIC, whereas in the co-culture (Fig 3) the bacteria can become labelled from both uptake of labelled DIC and labelled algal photosynthate. The continuous increase in *f*_*b*_ (Fig 3B) is therefore unlikely to come only from DIC uptake, suggesting instead that the bacteria assimilated ^13^*C* from labelled DOC produced by the algae. However, on their own, the SIMS results could not provide information on the precise carbon kinetics within the co-culture. In the early stages of a co-culture the question remains: are cells growing on mutually produced nutrients excreted into the media, on internal stores/organic media components or on nutrients carried-over in the media from the pre-culture? Combining SIMS data with our mechanistic model allowed this question to be addressed.

### Hidden nutrient kinetics

To further analyse the SIMS data and explore possible nutrient kinetics that couple the interaction partners, we formulated a mechanistic model of the algal-bacterial co-culture (as defined above) and performed parameter optimisations (see Table 1, Supplementary Methods in S1 Text, S5–S8 Tables, and S5 and S6 Figs). Fig 3B and 3C shows two separate global fits of the model to the algal and bacterial atomic fractions and cell densities. Fit 1 fixed the initial atomic fraction of ^13^*C* for DOC at *f*_*o*_(0) = 0.64, the expected value from the pre-labelled culture of algae (see Supplementary Results in S1 Text), whereas fit 2 included *f*_*o*_(0) as a free parameter. Fit 2 may appear to better describe the data, because it better reproduces bacterial growth, but the parameter optimisation result for *f*_*o*_(0) in fit 2 was close to natural abundance (S8 Table), which is not realistic for a culture expected to contain some labelled DOC from the highly labelled algal pre-culture. Neither fit was thus able to quantitatively capture the observations, which illustrates a trade-off between bacterial growth and isotope labelling that limited the effectiveness of the co-culture parameter optimisations. This suggests that our model is probably too simple to be fully quantitative. For example, the carbon yield for the bacterial cells *Y*_*b*,*c*_ might not be constant, internal storage could have contributed to bacterial growth or algal cell lysis could have provided a less enriched organic carbon source to the bacteria. Nonetheless, the results from fit 1 are qualitatively reasonable (the bacterial population is growing, albeit at a slower rate than expected), and could be used to predict the nutrient kinetics in the co-culture that are not directly inferable from our measurements.

**Fig 4:**
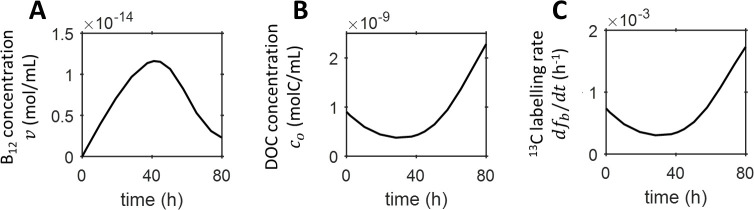
Nutrient kinetics in the co-culture predicted by the model. Concentrations of (A) B_12_ and (B) DOC in the co-culture predicted by the nutrient-explicit co-culture model using the parameter optimisation results obtained from fit 1 (Table 1). (C) Isotope labelling rate $\frac{df_{b}}{dt}$ calculated using Eq (18).

The co-culture medium was assumed to be initially vitamin-free because bacteria were washed thoroughly prior to establishing the co-culture and B_12_ was assumed to have been fully depleted in the 48 *h* pre-labelling culture of algae. This is a reasonable simplifying assumption to make since B_12_ measurements for axenic cultures of *C*. *reinhardtii* metE7 containing 100 *ng L*^−1^ B_12_ showed that the B_12_ remaining in the media reached its minimum after two days (S11 Fig). Using parameters from fit 1 (Table 1), the model revealed the potential B_12_ and DOC kinetics driving the microbial growth dynamics (Fig 4A and 4B). The vitamin concentration *v* increases from zero, peaks, and then starts to decrease after about 40 *h* (Fig 4A). Conversely, the DOC concentration *c*_*o*_ drops from the initial concentration *c*_*o*_(0), carried over from the unwashed algal pre-culture, to a local minimum, and then starts to rise after approximately 30 *h* (Fig 4B), a few hours before the turnaround in B_12_ concentration. These results can be interpreted in terms of the production/release and consumption of nutrients, and the resulting population growth. At the start of the experiment bacterial DOC uptake during growth was likely responsible for the initial depletion of DOC (Fig 4B), which occurred at a faster rate than could be replenished by the algae. The model results also suggest that growing bacteria were initially producing B_12_ faster than the algal uptake rate, allowing the vitamin concentration to increase (Fig 4A). As it did so, the algae grew and photosynthesised, producing DOC to be utilised by the bacteria, which proliferated in turn. The turnaround in the nutrient kinetics occurs when production/release and consumption rates are matched, seen mathematically by setting $\frac{dv}{dt}=0=\frac{dc_{o}}{dt}$ in Eqs (14) and (17). Fig 4A and 4B suggests that beyond the turning point at ≈30 *h*, DOC became more abundant as production by algae out-paced bacterial consumption. A short time later, the concentration of B_12_ began to decrease as release by bacteria fell below algal consumption.

Furthermore, the time evolution of the derivative of the bacterial atomic fraction $\frac{df_{b}}{dt}$ (obtainable from the model; Eq (18)) is seen to mirror closely the fall and rise of the DOC, reproducing a turnaround at approximately the same time (Fig 4C). The model implies that this is because the rate of DOC uptake by bacteria is proportional to the DOC concentration, such that a decrease in the DOC concentration decreases the uptake rate, which directly slows down the rate of ^13^*C* assimilation. Thus, the model, while not providing a fully quantitative description of the growth dynamics, is nevertheless able to predict the temporal variation of the nutrient kinetics from a minimal set of isotope labelling experiments. In future studies, the initial conditions for the model, e.g. the initial population densities *a*(0) and *b*(0), and thus the starting ratio *a*(0): *b*(0), could be varied to see how this influences the predicted timescales for the nutrient kinetics.

### Single cell heterogeneity

The SIMS results discussed thus far were averages obtained from several single cell measurements. We now turn to the heterogeneity in atomic fraction revealed by SIMS and not accessible by other methods such as EA-IRMS (see S3 and S4 Figs for histograms of the single cell data). For this we concentrated on bacteria which provided better statistics than algae (minimum 80 bacterial cells measured per time point, versus 5−29 cells per time-point for algae). For unlabelled bacteria at natural abundance the single cell measurements showed a narrow distribution of atomic fractions (S3 Fig), indicating that all bacteria started at approximately the same value. For axenic bacteria, increasing the glycerol concentration caused greater DIC uptake, and ^13^*C* was seen to be more widely spread across the population (S3 Fig). For the highest glycerol concentration, the cell distribution was seen to broaden and then narrow again over time, corresponding to the rise and fall of the mean atomic fraction, and a transition of the culture to stationary phase. In contrast, for bacteria in co-culture, the distribution of single cell atomic fractions broadened steadily over time (S4 Fig).

**Fig 5:**
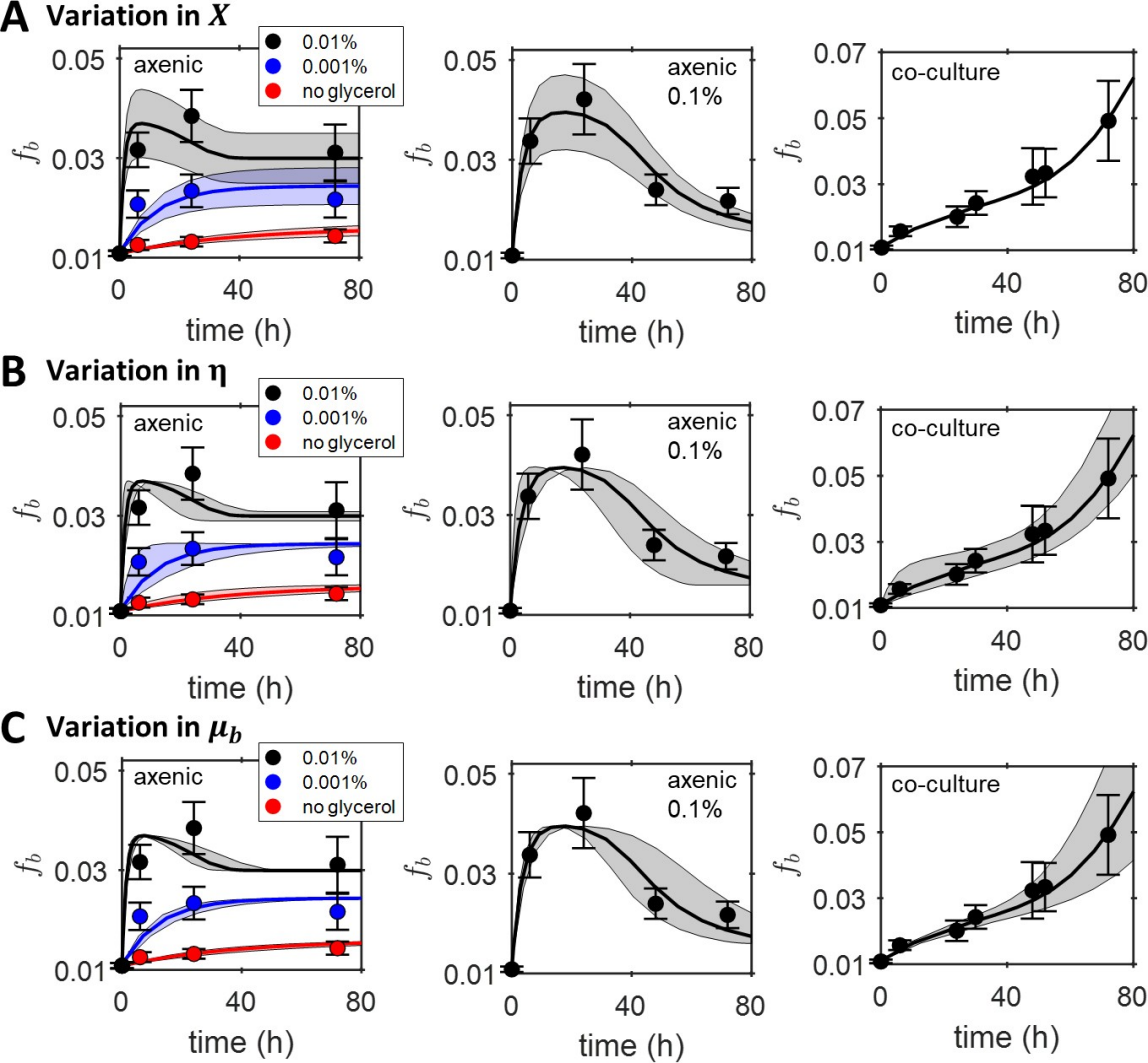
Comparison of single cell heterogeneity predicted by the model and measured experimentally with SIMS. The dilution-corrected results for the mean atomic fraction *f*_*b*_ obtained using SIMS (circles with error bars indicating standard deviations of single cell values). Solid lines indicate the model fit results, while shaded regions indicate predicted ranges of *f*_*b*_ values, when a range in a specific model parameter is considered. (A) The range of *X* values considered for 0.1%, 0.01%, 0.001% and no glycerol cultures of axenic bacteria were *X*∈[0.034,0.058], *X*∈[0.031,0.053], *X*∈[0.016,0.028] and *X*∈[0.007,0.011] respectively. For the co-culture the range considered was *X*∈[0.011,0.019]. (B) The range of *η* values considered for 0.1%, 0.01%, 0.001% and no glycerol cultures of axenic bacteria were *η*∈[0.21,0.81], *η*∈[0.05,0.25], *η*∈[0.09,0.69] and *η*∈[0.33,0.93] respectively. For the co-culture the range considered was *η*∈[0.11,0.91]. (C) For axenic cultures of bacteria *μ*_*b*_∈[0.11,0.19] and for the co-culture *μ*_*b*_∈[0.34,0.50] in units of *h*^−1^. Variation in *X* best accounts for the observed temporal trends in the standard deviations of the single cell data for the axenic cultures, whereas variation in *μ*_*b*_ best accounts for the co-culture results.

These single cell results clearly indicate heterogeneity in isotope labelling across the bacterial populations. To analyse heterogeneity, a stochastic, structured model would strictly be required, for example as was used to explain how the circadian clock and environmental cycles affect cell size control and generate two subpopulations in the cyanobacterium *Synechococcus elongatus* [49]. Our mean field model could, however, still be usefully applied to simulate heterogeneity and investigate potential origins of the observed single cell distributions by solving the model for parameter values above and below the fit results obtained for the mean atomic fractions (Table 1). Specifically, we considered the effect of varying the DIC uptake parameter *X*, bacterial maximum growth efficiency *η* and maximum bacterial growth rate *μ*_*b*_, with ranges given in the legend of Fig 5. The resulting variations in predicted bacterial atomic fractions (shaded areas in Fig 5) could then be compared with the variation observed experimentally, considered as the standard deviations of the SIMS single cell distributions (error bars in Fig 5). For axenic bacteria, a distribution in the values of *X* appeared to best account for the experimental variation (standard deviation) in the atomic fraction *f*_*b*_, especially for the culture grown at the highest glycerol concentration, where the model successfully reproduced the experimentally observed narrowing of the distribution at long times (Fig 5A). The comparison with experimental trends for variations in *η* and *μ*_*b*_ was less favourable (Fig 5B and 5C). Instead, for bacteria in co-culture, the progressive broadening of the distribution of *f*_*b*_ was best described by a distribution in *μ*_*b*_ (Fig 5C), with distributions in *η* and *X* not doing as well in the comparison (Fig 5A and 5B).

## Discussion

While several studies have demonstrated mutualistic interaction between bacteria and algae mediated by nutrient exchange [36,37,40], none have integrated time-resolved SIMS with mechanistic modelling to elucidate nutrient kinetics, as we have done here. Our approach allows the quantification of nutrient kinetics that control the inception and temporal development of an algal-bacterial mutualism. More broadly, this connects to the question of how co-occurrence can, on an evolutionary timescale, transform non-specialised relationships into more specialised partnerships, from streamlined microbial metabolisms [50–52] to plant-microbe interactions [53,54].

Initially, our SIMS-modelling approach demonstrated the uptake of labelled DIC by the heterotrophic bacterium *M*. *japonicum*, grown axenically on an unlabelled carbon source (glycerol). This confirmed similar results from previous studies of DIC uptake by heterotrophic bacteria [44,45], while also providing more extensive data in terms of temporal dynamics and concentration of organic carbon. Fractional DIC uptake, described by the parameter *X*, and respiration, described by the bacterial growth efficiency parameter *η*, were found to be essential for quantitatively describing the results. This was demonstrated by fitting the model to results of ^13^*C* labelling experiments, which provided values for these parameters. The DIC uptake parameter *X* was found to increase as a function of the exponential growth rate, and a faster exponential growth rate corresponded to a higher initial concentration of glycerol. The increase in *X* can be reasonably associated with an increase in the carboxylation reactions responsible for DIC acquisition with faster growth [44,45], however a more detailed model explicitly considering relevant metabolic processes like carboxylation reactions, respiration and carbon storage would be required to further investigate the functional relationships emerging from our data. For example, incorporating these metabolic processes more explicitly within our SIMS-modelling framework could lead to considering different metabolites that might in future be interesting to isotopically enrich in order to experimentally test different metabolic models. Following further testing on this and other model systems, our SIMS-modelling approach could be used in future studies to investigate how parameters like *X* and *η* are affected by environmental variables, including temperature, nutrient limitation and energetic quality of the organic carbon substrate [43,55].

The SIMS-modelling approach was then used to shed light on the role of nutrient exchange during the onset of mutualistic interaction in a co-culture of *M*. *japonicum* bacteria and vitamin B_12_-dependent *C*. *reinhardtii* metE7 algae, which has not been previously quantified. SIMS results showed that the labelling dynamics for bacteria in the co-culture was different to the axenic cultures. This shows that it is unlikely that the labelling of bacteria in the co-culture comes only form DIC uptake and suggests that bacteria assimilated algal-derived labelled carbon. Using our mechanistic model, we further revealed the nutrient kinetics that couple the mutualistic partners. Combining SIMS data with the model reasonably predicts that initial DOC in the co-culture (carried forward from the algal pre-culture) delayed the onset of reciprocal mutualistic interaction, defined to start when both partners are growing on reciprocally produced nutrients. Significantly, our combined SIMS-modelling approach also allowed us to quantify the delay: microalgae and bacteria started to grow exclusively on what each partner was producing only after about 30 *h* into the co-culture.

For comparison, a recent study by [56] quantified a cross-feeding mutualism between two yeast strains, each genetically engineered to release a metabolite required by the other. A lag phase of ~25h in the co-culture growth was observed: growth was initially slow and, following the lag, became faster. Pre-starving one of the yeast strains reduced the lag phase. It should be stressed that the lag found by [56] is defined in terms of growth rate of cells washed free of excess nutrients prior to co-culture inoculation, which is distinct from the delay we have reported. The latter is a delay directly associated with the nutrient kinetics underlying a mutualistic interaction and specifically defines the shift from growth using internal stores or nutrients carried over from pre-cultures to growth dependent on the exchange of nutrients between the interaction partners. These kinetics would be difficult to observe using current methods (e.g. it is not possible to measure them using growth measurements alone or by probing gene activation), but can naturally be studied using our combined modelling and SIMS method. Compared to other synthetic studies [21–23], our method will also be advantageous for relating microbial growth and nutrient dynamics, and for studying interactions between wild-type microorganisms, including environmental species, that are not possible to genetically engineer, but for which it is straight forward to isotope label nutrients supporting their growth.

We emphasise that the present study considers the onset of a mutualistic interaction between algae and bacteria, which interacted in co-culture for only a few generations. This contrasts with recent experimental evolution investigations on cross-feeding bacteria that have demonstrated how, over hundreds of generations, the production of communally valuable nutrients can stimulate the metabolism of mutualistic microbial partners, increasing the productivity and stability of the partnerships [57–60]. In our study, *C*. *reinhardtii* metE7 and *M*. *japonicum* have not had time to evolve and metabolically adapt to each other. Instead, their interaction seems predicated purely on the exchange of vitamin B_12_ and DOC. Indeed, DOC necessary for bacterial growth was found to be present in the supernatant of axenic algal cultures (S12 Fig); conversely, B_12_ levels in the media of axenic cultures of *M*. *japonicum* were found to be substantial and sufficient to sustain algal growth [47]. Furthermore, the same study demonstrated that B_12_ production by *M*. *japonicum* is no greater on a per cell basis when cocultured with metE7 than when cultured axenically with glycerol; this suggests that there is no induction of B_12_ synthesis by *metE7*. The interaction we have studied is thus a simple cross-feeding of metabolites released in suspension (as described by our model) and represents the very first stage of what could evolve into a metabolically more complex relationship, as shown for bacteria [57–60], should the microbial partners continue to interact.

Exploiting the single cell resolution of SIMS, our results also revealed the heterogeneity of carbon uptake across a bacterial population. The distribution of atomic fractions for axenic bacteria displayed a width (standard deviation) that was non-monotonic with time, whereas for the bacteria in co-culture with algae, this width increased monotonically. To explain these trends, we simulated the variation of phenotypes across the bacterial population using our model, which was solved with parameter values above and below the fit results. A distribution in inorganic carbon uptake parameter *X* gave the best agreement with experiment for axenic cultures, whereas a distribution in bacterial growth rate *μ*_*b*_ best accounted for the co-culture measurements. Regarding the latter, it could reflect the heterogeneous environment for bacteria growing on algal exudates comprising a mix of compounds, each corresponding to a different growth rate. Conversely, axenic bacteria were fed on a single carbon substrate. Future studies could compare structured mechanistic models and computer simulations that describe variation in population dynamics and nutrient kinetics across microbial populations [49,61,62] with the approach to modelling heterogeneity used here.

After a broader validation and potential refinement of the model, our approach of combining mathematical modelling with isotope labelling and EA-IRMS/SIMS can be applied to revealing quantitative details in microbial interactions, both in the laboratory and the environment. The approach is particularly relevant in situations where several metabolic processes are simultaneously present and therefore make carrying out several individual isotope labelling measurements difficult. Our model offers a simple and mechanistic platform that would not require significant modifications to describe a variety of microbial systems, including those involving several species, provided the relevant biological parameters can be determined. Table 2 summarises the steps required for applying our method to such systems.

**Table 2:** Summary of how to apply our method to other microbial systems.

**Modelling** • Adapt our model to reflect the relevant species and nutrients of interest. In summary, this involves ○ Defining nutrient dependent growth rates for each species. ○ Defining nutrient uptake and release rates. ○ Considering metabolic details relevant to the description of the nutrient dynamics. • Consider what nutrients could be isotopically labelled and obtain from the model equations for the isotope dynamics.
**Independent parameterisation** • Perform growth experiments to determine growth-related parameters like carrying capacity and maximum growth rate. • Obtain as many of the model parameters as possible from independent experimental measurements e.g. carbon yield from EA-IRMS.
**Isotope labelling experiments** • Perform isotope labelling experiments using SIMS, EA-IRMS or other techniques to fit the remaining parameters and investigate the nutrient dynamics [a].

[a] We suggest that it could be advantageous to first perform bulk isotope analysis like EA-IRMS for parameter optimisation and studying axenic cultures in different conditions (e.g. nutrient concentrations, light intensity, temperature). Further investigation of more intricate nutrient dynamics could then be performed using SIMS, which has the advantage of more easily studying multiple time points from the same culture (because smaller sample volumes are required compared to EA-IRMS) and can reveal single cell heterogeneity.

For example, it would be interesting to apply our method to study tripartite interactions between bacteria, microalgae and fungi [63] involving the exchange of key metabolites such as carbon compounds and nitrates (that could be followed using isotope labelling), and vitamins (i.e. using bioassays to test model predictions). This would accelerate discovery towards a mechanistic understanding of microbial interactions and, in complement with top-down omics approaches [17,18], move towards application of our approach to complex synthetic and environmental microbial communities.

## Materials and methods

### Algal and bacterial strains

The B_12_-dependent alga used in this work was *C*. *reinhardtii* metE7 [38]. The B_12_-producing bacterium used was *M*. *japonicum* (MAFF 303099), previously named *Mesorhizobium loti* [39], originally a gift from Prof Allan Downie, John Innes Centre, UK. We used viable counts on enriched plates and microscopy (nucleic acid staining and fluorescence imaging) to check that contaminants were not growing in our cultures.

### Growth conditions

Cultures were grown in a 12 *h*−12 *h* light-dark cycle at 25°*C*, shaking at 120 *rpm*. The intensity of the photosynthetically active radiation was approximately 70 μ*mol m*^−2^ *s*^−1^, measured using a Skye PAR sensor (SKP 215). Tris-minimal medium was used for all cultures, meaning that *C*. *reinhardtii* metE7 grew phototrophically in our experiments. Tris-minimal medium is based on TAP [64] but omits the acetic acid and *HCl* is used to titrate to *pH* 7 [65]. Trace element solutions (S1 Table) were adapted from [66] to include cobalt, since it is required as the central ion of vitamin B_12_. The cobalt concentration was chosen to be the same as in Hutner’s trace elements [67]. Cyanocobalamin (referred to as B_12_ throughout this work), glycerol and sodium bicarbonate were added to the medium as required (S2 Table).

Dissolved sodium ^13^*C*-bicarbonate (Sigma-Aldrich *NaH*^13^*CO*_3_, 98 *atm*% ^13^*C*) was used for the stable isotope labelling of microbial cultures (the work-flow is illustrated in S1 Fig). A sample from the 600 *mL* axenic pre-culture of algae was washed and re-suspended in 1 *L* of fresh media containing 7.5×10^−14^ *mol mL*^−1^ B_12_ (i.e. 100 *ng L*^−1^ B_12_) and 5 *mM NaH*^13^*CO*_3_. This pre-labelling culture of algae was grown for 48 *h* (see Supplementary Information for the experimental and model results for this culture). An axenic pre-culture of bacteria was grown in media with 0.1% (*v*/*v*) glycerol, which was then sampled, washed and re-suspended in 750 *mL* fresh media containing 5 *mM NaH*^13^*CO*_3_, to which 250 *mL* of pre-labelled algae was added to initiate the co-culture. The algae were not washed prior to co-culture inoculation, meaning that DOC was carried forward from the algal pre-culture. This was done to have the best chance of observing uptake of algal photosynthate by *M*. *japonicum*. In order to also improve the chances of achieving a reciprocal mutualism within the time frame of the experiment, a relatively high inoculum of bacteria was chosen. The starting ratio of bacterial:algal cells was approximately 1000:1. The long-term ratios at which the algae and bacteria stably coexist and mutually support one another fluctuates between 10 and 100 bacterial cells to each algal cell [47]. *M*. *japonicum* viable cell density tends to hold steady even after all carbon sources are removed from the medium, so although there may be some lysis at the onset of the co-culture it is unlikely to be very substantial. Furthermore, [68] showed that lysis is unlikely to be a sufficient path to delivering B_12_ to B_12_ dependent algae. Cultures of axenic bacteria were grown with 5 *mM NaH*^13^*CO*_3_ and different concentrations of unlabelled glycerol.

### Population growth

Population growth was monitored using viable counts. A series of 10-fold dilutions were performed and aliquots of 20 μ*L* from relevant dilutions (i.e. chosen such that approximately 10 to 100 colonies would result after plating) were spotted onto TY agar plates. The plates were tilted back and forth to disperse the cells and make the colonies easier to distinguish [69]. Plates were incubated in continuous light at 25°*C* for approximately 5 days and in the dark at 30°*C* for approximately 2 days, for algal and bacterial colonies respectively. Two independent viable counts were obtained for each time-point and the results converted to values for the population size in units of colony forming units per unit volume (*cfu mL*^−1^). We note that viable counts cannot detect dormant cells, but provided reasonable estimates of population size, and particularly the subset that contributes to carbon uptake.

### Isotope Ratio Mass Spectrometry

Elemental Analysis-Isotope Ratio Mass Spectrometry (EA-IRMS) was used to measure ^13^*C* ratios for bulk samples of algal and bacterial biomass. The analysis was performed by the Godwin lab, Department of Earth Sciences, University of Cambridge using the Thermo Delta V Plus and Costech. EA-IRMS also measured total carbon and nitrogen content, which was used to calculate the C:N ratio and, together with dry mass and cell density measurements, to estimate the carbon yield (i.e. *cells molC*^−1^) for algae and bacteria; see Supplementary Methods in S1 Text and S4 Table for details.

### Secondary ion mass spectrometry

#### Sample preparation

We here briefly outline the SIMS sample preparation procedure; full details are in Supplementary Methods in S1 Text. Samples were chemically fixed using formaldehyde. Vacuum filtration was used to deposit the cells onto 0.22 μ*m* pore size membrane filters with a ≈20 *nm* gold coating. Nucleic acid staining and confocal microscopy (Olympus Fluoview FV1200) were used to confirm an even distribution of cells on the filter. A single hole punch was used to cut out 4−6 *mm* disks from the filter samples. Following this, a Zeiss laser micro-dissection (LMD) microscope (Zeiss LSM710-NLO, LCI facility, Karolinska Institute, Stockholm) was used to image the autofluorescence of the algal chlorophyll and create laser marks on the samples, which were used to easily and quickly locate areas of interest with the SIMS instrument camera. These LMD images were used to identify algal cells in the SIMS images and allowed individual algal cells to be differentiated from bacterial microcolonies. Lastly, samples were placed on conductive sticky tape, mounted onto a glass disk and sputter coated with gold.

#### SIMS analysis

SIMS analysis was performed using the CAMECA IMS 1280 at the NordSIM facility (Department of Geosciences, Swedish Museum of Natural History, Stockholm). The instrument uses a Gaussian focussed primary ion beam of caesium ions (*Cs*^+^). For selected positions on the filter sample, 45×45 μ*m* square areas were pre-sputtered for 10 *s* with a 3 *nA* primary ion beam. Within this pre-sputtered region, 100 scans of a 35×35 μ*m* square area were measured using a ≈60−80 *pA* primary ion beam (spot size of approximately 1 μ*m*). The secondary ion mass peaks were measured using an ion counting electron multiplier in peak hopping mode with a 44 *ns* electronically gated dead-time. The count times for the ^12^*C*^14^*N*^−^, ^12^*C*^15^*N*^−^ (not used in subsequent analysis) and ^13^*C*^14^*N*^−^ secondary ion peaks were 1, 0.5 and 2 *s* respectively. A mass resolution (*M*/Δ*M*) of approximately 6000 for the preliminary experiments (results shown in S10 Fig) and 7000 for the final experiments was used; a mass resolution of 6000−7000 was sufficient in resolving both the ^12^*C*^14^*N*^−^ and ^13^*C*^14^*N*^−^ peaks. Interference of ^11^*B*^16^*O*^−^ with the ^13^*C*^14^*N*^−^ peak at mass 27 was not an issue because no boron or boron containing compounds were used in the culture media and we measured natural abundance (i.e. mean and standard deviation values of 0.01110±0.00004 and 0.0108±0.0002 for algae and bacteria respectively) in the unlabelled controls. The SIMS measurements were run once for bacterial cells and repeated 1−8 times for each algal cell. The WinImage2 software (CAMECA) was used to obtain the isotope ratio *R* = ^13^*C*/^12^*C* for single cells of algae and bacteria (see Supplementary Methods in S1 Text for details). The atomic fraction of ^13^*C*, i.e. *f* = ^13^*C*/(^13^*C*+^12^*C*), was calculated using

$$f=\frac{R}{1+R}.$$ (19)

Several technical considerations were taken into account (see Supplementary Methods in S1 Text and S2 Fig for full details). First, a depth analysis was performed by taking repeated measurements of the same cells, which demonstrated that a single measurement was sufficiently representative for bacteria, whereas for algal cells the mean of three repeated measurements was used to obtain a representative measurement. Second, a scattering effect associated with highly labelled algae was observed, therefore for the analysis described in this work only bacteria from scan areas not containing labelled algae were included. Lastly, the dilution effect, due to chemical fixation and nucleic acid staining introducing unlabelled carbon into cells, was taken into consideration (S3 Table). To estimate the undiluted atomic fraction of ^13^*C*, SIMS results were *dilution-corrected* using the method established in [70].

## Supporting information

S1 Fig Work-flow for the stable isotope labelling cultures. Schematic overview and time-line of the stable-isotope labelling cultures using the alga *C*. *reinhartii* metE7 and the bacterium *M*. *japonicum*, as described in detail in the text. The vertical white and grey bars indicate the 12 *h* light and 12 *h* dark periods respectively. Samples were taken at different time-points for single cell carbon isotope analysis using SIMS and bulk carbon isotope analysis of the algal and bacterial biomass using EA-IRMS.
(TIF)

S2 FigTechnical considerations for SIMS experiments.(A) The difference between the atomic fraction of ^13^*C* in algal cells obtained from the third and first measurements (Δ*f* = *f*_3_−*f*_1_) relative to the mean ($\bar{f}=(f_{1}+f_{2}+f_{3})/3$). These results show that the carbon isotopes are not homogeneously distributed within the algal cells. (B) Example SIMS result for the ^12^*C*^14^*N* isotope images of bacteria obtained for two repeated measurements at the same sample location. The colour maps indicate the scale for the SIMS measurements in units of secondary ion counts, which were accumulated over 100 scans. These results imply that the majority of the bacterial biomass is sputtered away during the first measurement. (C) Comparison between the mean and standard deviation of the atomic fraction of ^13^*C* in bacterial cells (〈*f*〉 and *σ*_*f*_ respectively) for scan areas of co-culture samples that do not contain any highly labelled algal cells (black bars) and areas that contain at least one highly labelled algal cell (green bars). These results imply a scattering effect causes the atomic fractions of ^13^*C* obtained for bacterial cells to be both higher and more variable when the scan area contains a labelled algal cell. (D) Atomic fraction of ^13^*C* obtained by EA-IRMS and SIMS analysis (black diamonds) for both algae (left) and bacteria (right), with the red lines showing the results of the least squares fit of Eq (S4) in Supplementary Methods in S1 Text, using *f*_*ch*_ = 0.0108 (S3 Table). The *D* = 0 case is plotted (black dotted line) to show that if there was no dilution effect the EA-IRMS and SIMS results would be expected to give the same results. The dilution effect means that the SIMS measurements provide an underestimate of the true, undiluted *f*.(TIF)

S3 Fig Histograms for the SIMS results of bacterial cells grown in axenic cultures. Histogram plots showing the dilution-corrected SIMS results for the single cell measurements of *f*. The number of cells (n) analysed and included in the calculation of the mean is indicated for each time point. The red stars indicate the points that were considered outliers and therefore excluded from the calculation of the mean. These small number of data points (i.e. 4 points for the 6 *h* sample from the no glycerol culture and 1 point for the 6 *h* sample from the 0.001% glycerol culture) with a relatively high atomic fraction of ^13^*C* could be the result of experimental artefacts like sub-resolution organic matter debris or cross-contamination between samples, see Supplementary Methods in S1 Text for details. Note that different scales have been used for the vertical axes.
(TIF)

S4 Fig Histograms for the SIMS results of algal and bacterial cells grown in co-culture. Histogram plots showing the dilution-corrected SIMS results for single cell measurements of the atomic fraction of ^13^*C* for algal and bacterial cells at different time-points of the co-culture. The number of cells (n) analysed and included in the calculation of the mean is indicated for each time point. The red bars indicate the algal cells that were considered ‘outliers’ and not included in the calculation of the mean because they were close to natural abundance and therefore considered inactive. The red stars indicate the of bacteria data points that were considered outliers and therefore excluded from the calculation of the mean. These outliers (i.e. 1 point for the 6 *h* sample and 3 points for the 48 *h* sample) could be the result of experimental artefacts like sub-resolution organic matter debris or cross-contamination between samples, see Supplementary Methods in S1 Text for details. Note that different scales have been used for the vertical axes.
(TIF)

S5 Fig Parameter optimisation for a simplified co-culture model. Fit of data obtained for co-cultures of *C*. *reinhardtii* metE7 and *M*. *japonicum*. Each row of plots corresponds to an independent experiment, with the first column the evolution of algal density, in the second column the evolution of bacterial density and in the third column the evolution of vitamin concentration determined by a bioassay, as described in [71]. The mean of each variable appears as a continuous line, with the shaded region showing the standard deviation for 8, 4 and 5 replicates for experiment 1, 2 and 3 respectively. The global fit with a unique set of parameters for the three independent experiments is shown in black dashed lines. The horizontal axes show time in days.
(TIF)

S6 Fig Logistic growth fit for *M*. *japonicum*. Data taken from [40] for *M*. *japonicum* grown axenically in 0.1% glycerol was fit with the logistic growth equation *b* = *K*_*b*_/(1+*M e*^−*r t*^), with *M* = 2.8×10^4^, *r* = 3.9 and carrying capacity *K*_*b*_ = 1.3×10^9^. No error bars are shown because the fractional errors for the data points were within 20%, and appear smaller than a data point on the graph.
(TIF)

S7 Fig Fit for the exponential growth rate of bacteria. Plotted points show the viable count results for the growth of *M*. *japonicum* in the co-culture (black) and in the axenic culture grown without glycerol (grey). The dotted lines indicate the results for the exponential growth fit using equation *b* = *b*(0) exp(*μ*_*B*_ *t*), giving *b*(0) = 1.2±0.01×10^7^ *cfu mL*^−1^ and *μ*_*B*_ = 0.022±0.005 *h*^−1^ for the co-culture and *b*(0) = 1.5±0.01×10^7^ *cfu mL*^−1^ and *μ*_*B*_ = 0.012±0.004 *h*^−1^ for the axenic culture.
(TIF)

S8 Fig For axenic bacteria the DIC uptake parameter and bacterial growth efficiency depend on the initial exponential growth rate. (A) The relationship between the DIC uptake parameter *X* and the initial exponential growth rate *μ*_*B*_ = *μ*_*b*_ *c*_*o*_(0)/(*c*_*o*_(0)+*K*_*c*_) was approximated with a logarithmic fit using equation *X* = *m* ln(*μ*_*B*_)+*n*, giving *m* = 0.0167±0.0004 and *n* = 0.0785±0.0013, with *R*^2^ = 0.999. (B) The relationship between the bacterial growth efficiency *η* and *μ*_*B*_ was approximated with a logarithmic fit using equation *η* = *p* ln(*μ*_*B*_)+*q*, giving *p* = −0.10±0.12 and *q* = 0.12±0.36, with *R*^2^ = 0.282.
(TIF)

S9 Fig The pre-labelling, axenic culture of *C. reinhardtii* metE7. (A) Example images of the atomic fraction of ^13^*C*, *f*, obtained by SIMS analysis of algal cells sampled at different time-points of the pre-labelling, axenic culture grown with 5*mM NaH*^13^*CO*_3_. The colour map shows the scale, starting at natural abundance. (B) Histogram plots showing the dilution-corrected SIMS results for single cell measurements of the atomic fraction of ^13^*C* in individual algal cells. The number of cells (n) analysed and included in the calculation of the mean is indicated for each time point. The red bars indicate the algal cells that were considered ‘outliers’ and not included in the calculation of the mean because they were close to natural abundance and therefore considered inactive. Note that different scales have been used for the vertical axes. (C) The mean atomic fraction of ^13^*C* for the dilution-corrected SIMS measurements (diamonds). Error bars, showing the standard error, are small compared to the size of the plotted points. (D) Algal growth measured using viable counts (*cfu mL*^−1^), plotted on a logarithmic scale as the mean and standard error of two measurements (diamonds). The results of the model fit, with parameters and initial conditions as specified in Table 1, are also plotted for (C) *f*_*a*_ and (D) *a*.
(TIF)

S10 Fig Comparison of the SIMS results for the preliminary experiment, final experiment and unlabelled control cultures. Preliminary SIMS results (grey circles) for the mean carbon isotope fraction are compared with the results from the final experiment (black circles) for the pre-labelling cultures of algae grown with 5 *mM NaH*^13^*CO*_3_, axenic cultures of bacteria grown with 0.1% glycerol and 5 *mM NaH*^13^*CO*_3_ and the algal-bacterial co-culture. Error bars correspond to the standard errors. In the preliminary experiment, control cultures of axenic algae and a co-culture were grown with 5 *mM* unlabeled *NaHCO*_3_ and a sample at 48 *h* was taken and analysed using SIMS to show that the cells remained at natural abundance (red circles). For algal cells, the preliminary experiment only obtained one SIMS measurement, whereas for the final experiment the values plotted represent the mean value for algal cells where the single cell values are the mean of 2–3 repeated SIMS measurements taken at the same location on the filter. The SIMS results presented here have not been dilution-corrected. Note that different scales have been used for the vertical axes.
(TIF)

S11 Fig Growth and B12 uptake of *C. reinhardtii* metE7 in axenic cultures containing *7.5×10^−14^ mol*/*mL* B12 (i.e. *100 ng*/*L*). (A) Growth of *C*. *reinhardtii* metE7 in Tris minimal medium + 7.5×10^−14^ *mol*/*mL* B_12_ measured by counting cell density using a Z2 particle count analyser (Beckman Coulter Ltd). (B) Total B_12_ measured in the cells and media of the *C*. *reinhardtii* metE7 cultures. (C) B_12_ remaining in the media of the *C*. *reinhardtii* metE7 cultures. A bioassay, as described in [71], was used to quantify the B_12_ concentration, which measured the growth of a B_12_-dependent *Salmonella typhimurium* strain AR3612 when incubated with the sample of interest. Note that the B_12_ remaining in the media reaches a low point of roughly 1×10^−14^ *mol*/*mL* B_12_ after 2 days. Why this does not decrease to zero is unclear. Perhaps the affinity of the algal B_12_ uptake system is not high enough, or the bioassay overestimates B_12_, particularly when there is a lot of algal debris in the media. Error bars: standard deviation (n = 4).
(TIF)

S12 Fig The effect of *C*. *reinhardtii* metE7 on the growth of *M*. *japonicum*. *M*. *japonicum* was cultured in 20 mL TP medium + 0.001% glycerol at 25°C and 12-hour light (100 μE·m^-2^·s^-1^) and 12-hour dark cycles with 120rpm rotational shaking. After *M*. *japonicum* cultures reached stationary phase, 2 mL of 1) stationary phase metE7 cultured in the same manner but with 200 ng·L^-1^ B_12_ instead of 0.001% glycerol was added to one set of *M*. *japonicum* cultures (black solid line), 2) the same metE7 culture filtered through a 0.4 μm filter (to remove the cells) was added to the second set (black dashed line), 3) TP medium was added to the third set (grey line). *M*. *japonicum* CFUs were measured before and after addition of these cultures. Error bars = standard deviation, n = 3.
(TIF)

S1 Table Trace elements adapted from (Kropat et al. 2011). The concentrations of the different chemical components for each of the seven stock solutions of trace elements for the Tris-minimal media used in this work. For 1 *L* of Tris-minimal media, 1 *mL* of each solution was added.
(DOCX)

S2 Table List of cultures. A complete list of the cultures grown as part of the stable isotope labelling experiments described in this work. Tris-minimal growth medium was used for all cultures with the addition of B_12_, glycerol and sodium bicarbonate as listed in this table. These cultures were grown in 2 *L* conical flasks except for the pre-cultures, which were grown in 1 *L* flasks. Note that for B_12_ concentrations 1 *ng*/*L* = 7.5×10^−16^ *mol*/*mL*.
(DOCX)

S3 Table The dilution factor results. The dilution factor, *D*, was obtained from a least squares fit of Eq (S4) in Supplementary Methods in S1 Text using the curve fitting application in Matlab and with *f*_*ch*_ = 0.0108. This table lists the results for *D*, the 95% confidence bounds, the number of points in the fit, *n*, and the least square displacements, *R*^2^. For bacteria, the fit was carried out using only data from axenic cultures.
(DOCX)

S4 Table C-N content and carbon yield (*Y*_*a*,*c*_ and *Y*_*b*,*c*_ for algae and bacteria respectively). Summary of the EA-IRMS results for the carbon and nitrogen content and the carbon yield for the alga *C*. *reinhardtii* metE7 and bacterium *M*. *japonicum*. The table gives the mean, standard error and the number of samples included in the mean (*n*).
(DOCX)

S5 Table Fitting intervals for the parameter optimisations. These were the fitting intervals used for the various free parameters and free initial conditions of the parameter optimisations run for axenic algae, axenic bacteria and the co-culture. When units are not specified the parameter/initial condition is in dimensionless units. The fitting intervals for *ϕ*_*s*_, *η* and *X* came from their definition requiring these parameters to be between 0 and 1. Other fitting intervals were chosen to ensure that the parameter optimisation results were reasonable when considering their biological interpretation. In particular, the choice of fitting intervals was informed by the physiologically relevant parameter ranges for the mutualistic association of *M*. *japonicum* and *L*. *rostrata* provided by (Peaudecerf et al. 2018).
(DOCX)

S6 Table Non-dimensional model parameters. The non-dimensional model parameter definitions and estimated values for *C*. *reinhardtii* metE7 and *M*. *japonicum* grown both axenically and in co-culture.
(DOCX)

S7 TableCulture specific model parameters and initial conditions for axenic bacteria.Model parameter values for the axenic cultures of *M*. *japonicum* grown with different concentrations of glycerol determined by a global parameter optimisation performed for the four axenic cultures of *M*. *japonicum* grown with 0.1%, 0.01%, 0.001% and no glycerol. The global free parameters were *μ*_*b*_ and *K*_*c*_, which were constrained to have the same value for all four cultures. The free parameters and initial conditions that were permitted to be different for the different cultures were *η*, *X* and *b*(0). The initial DOC concentration *c*_*o*_(0) for the culture grown without glycerol was also included as a free parameter. The fixed initial conditions were ${\hat{c}}_{i}(0)=5,\;\hat{v}(0)=0,$ *f*_*b*_(0) = 0.0108, *f*_*o*_(0) = 0.0108, and *f*_*i*_(0) = 0.65, since for the experiments it was assumed that the DIC was in excess, initially there was no B_12_ and the bacteria had natural abundance, the glycerol was unlabelled and the atomic fraction of ^13^*C* in the DIC was taken as the estimate obtained from the parameter optimisation for axenic algae (see Table 1). The residual sum of squares for this global parameter optimisation result was 0.58, whereas when respiration was not included in the model it was 2.24.(DOCX)

S8 TableComparison of parameter optimisation results for the algal-bacterial co-culture.Results of different parameter optimisation results for the co-culture between *C*. *reinhardtii* metE7 and *M*. *japonicum*. The only free parameter was *s*_*c*_ and the free initial conditions were $\hat{a}(0),\;\hat{b}(0)$ and ${\hat{c}}_{o}(0)$, with ${\hat{f}}_{o}(0)$ included as an additional free initial condition for fit 2. The parameters *ϕ*_*s*_ = 0.9, *η* = 0.51 and *X* = 0.015 were estimated using results from axenic cultures as specified in the text. All other parameters had values as specified in Table 1. The fixed initial conditions were ${\hat{c}}_{i}(0)=5$ (i.e. DIC concentration in excess), $\hat{v}(0)=0$ (i.e. initially no B_12_ in the media), *f*_*a*_(0) = 0.59 and *f*_*a*,*p*_(0) = 0.65 (i.e. using model results for the pre-labelling, axenic culture of algae, see text for details), *f*_*b*_(0) = 0.0108 (i.e. bacteria initially have natural abundance), and *f*_*i*_(0) = 0.65 (i.e. from the parameter optimisation result for axenic algae, see Table 1).(DOCX)

S1 Text A file that includes the supplementary methods and supplementary results. (DOCX)
