# Supplementary figures and images for: Combining SIMS and mechanistic modelling to reveal nutrient kinetics in an algal-bacterial mutualism
Source: PLoS One. 2021 May 20;16(5):e0251643. doi: 10.1371/journal.pone.0251643 (PMC8136852; doi:10.1371/journal.pone.0251643)

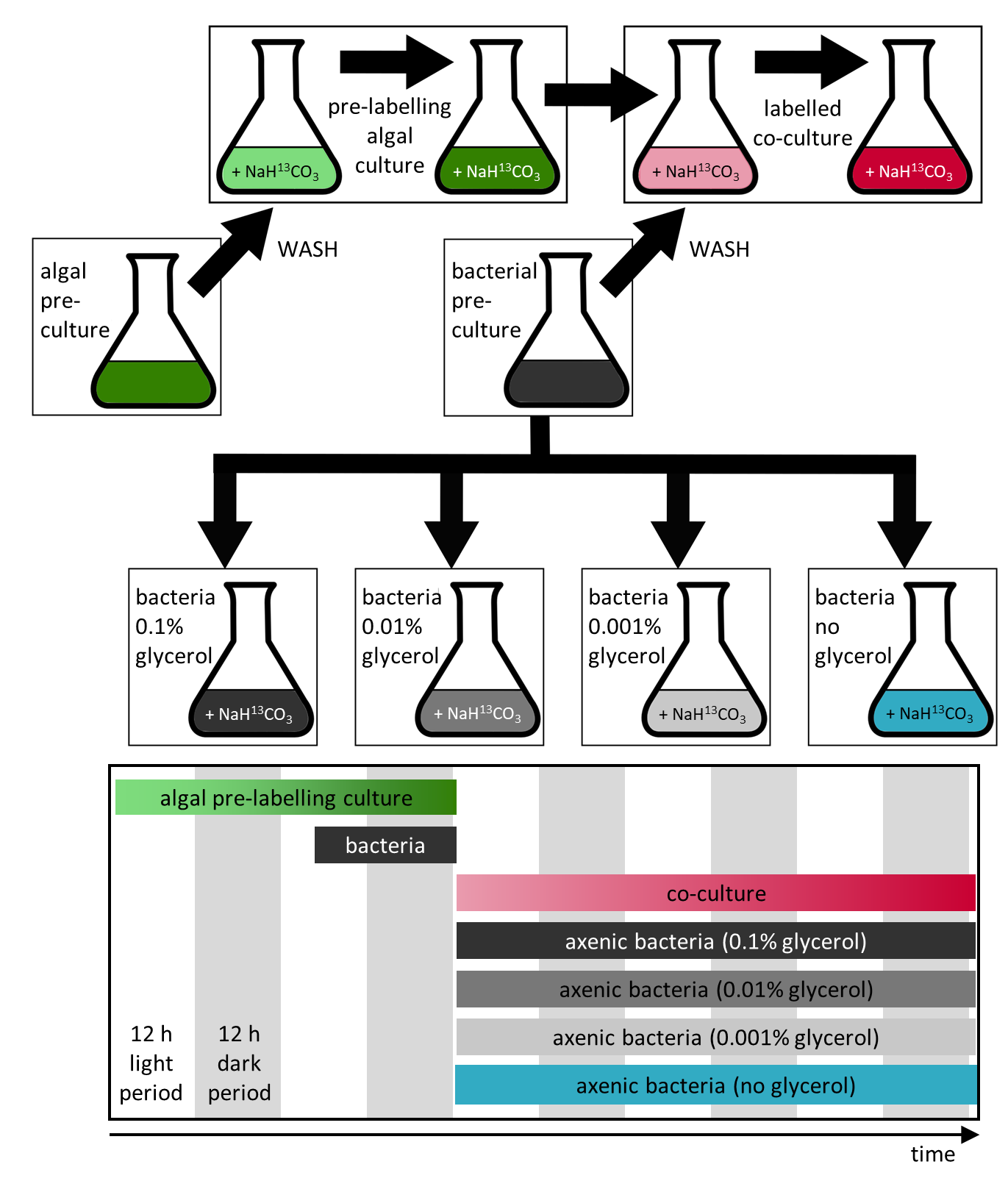

Supplement: S1 Fig — Schematic overview and time-line of the stable-isotope labelling cultures using the alga C. reinhartii metE7 and the bacterium M. japonicum, as described in detail in the text. The vertical white and grey bars indicate the 12 h light and 12 h dark periods respectively. Samples were taken at different time-points for single cell carbon isotope analysis using SIMS and bulk carbon isotope analysis of the algal and bacterial biomass using EA-IRMS. (TIF) [file pone.0251643.s001.tif]

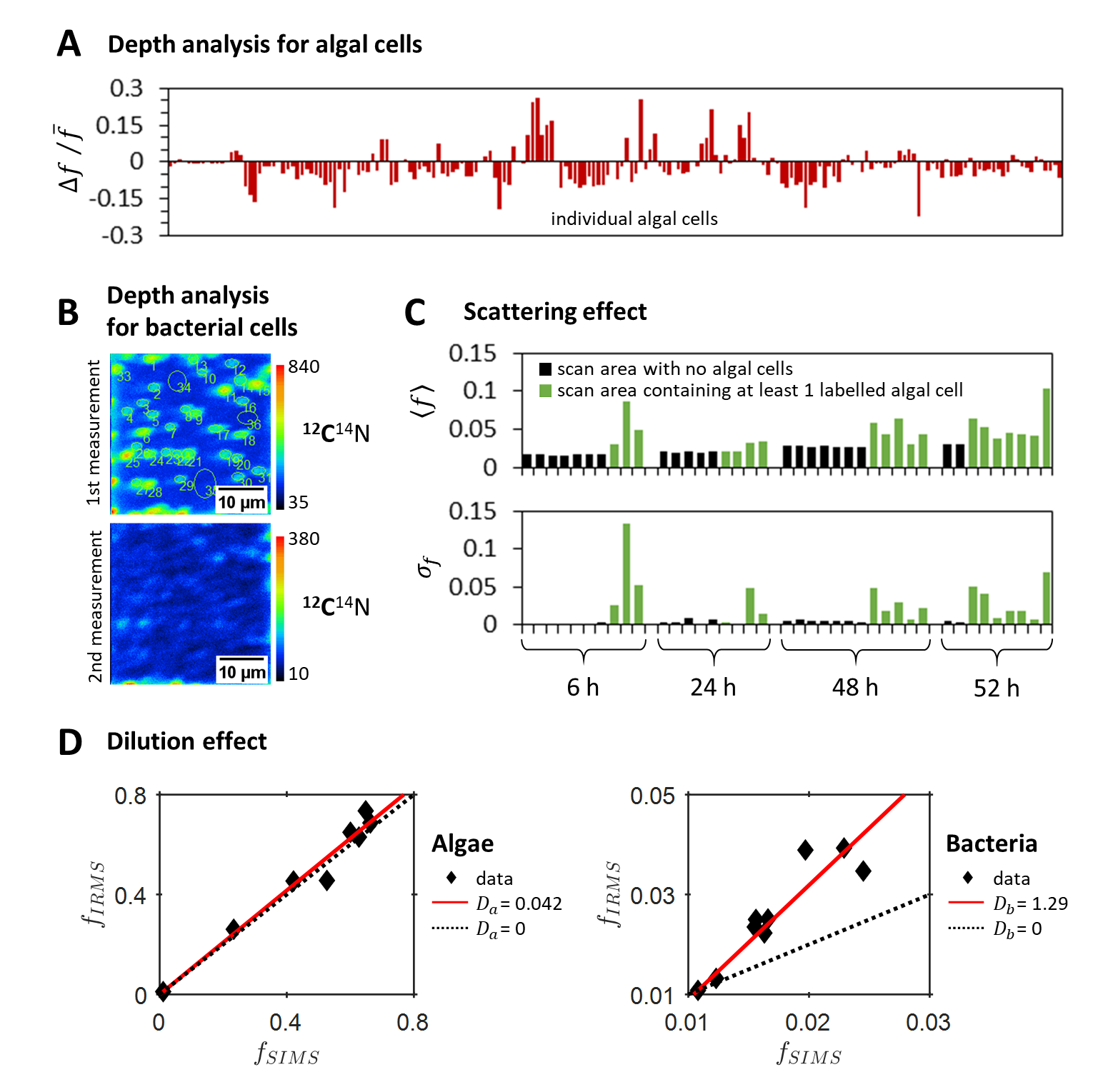

Supplement: S2 Fig — (A) The difference between the atomic fraction of 13C in algal cells obtained from the third and first measurements (Δf = f3−f1) relative to the mean (f¯=(f1+f2+f3)/3). These results show that the carbon isotopes are not homogeneously distributed within the algal cells. (B) Example SIMS result for the 12C14N isotope images of bacteria obtained for two repeated measurements at the same sample location. The colour maps indicate the scale for the SIMS measurements in units of secondary ion counts, which were accumulated over 100 scans. These results imply that the majority of the bacterial biomass is sputtered away during the first measurement. (C) Comparison between the mean and standard deviation of the atomic fraction of 13C in bacterial cells (〈f〉 and σf respectively) for scan areas of co-culture samples that do not contain any highly labelled algal cells (black bars) and areas that contain at least one highly labelled algal cell (green bars). These results imply a scattering effect causes the atomic fractions of 13C obtained for bacterial cells to be both higher and more variable when the scan area contains a labelled algal cell. (D) Atomic fraction of 13C obtained by EA-IRMS and SIMS analysis (black diamonds) for both algae (left) and bacteria (right), with the red lines showing the results of the least squares fit of Eq (S4) in Supplementary Methods in S1 Text, using fch = 0.0108 (S3 Table). The D = 0 case is plotted (black dotted line) to show that if there was no dilution effect the EA-IRMS and SIMS results would be expected to give the same results. The dilution effect means that the SIMS measurements provide an underestimate of the true, undiluted f. (TIF) [file pone.0251643.s002.tif]

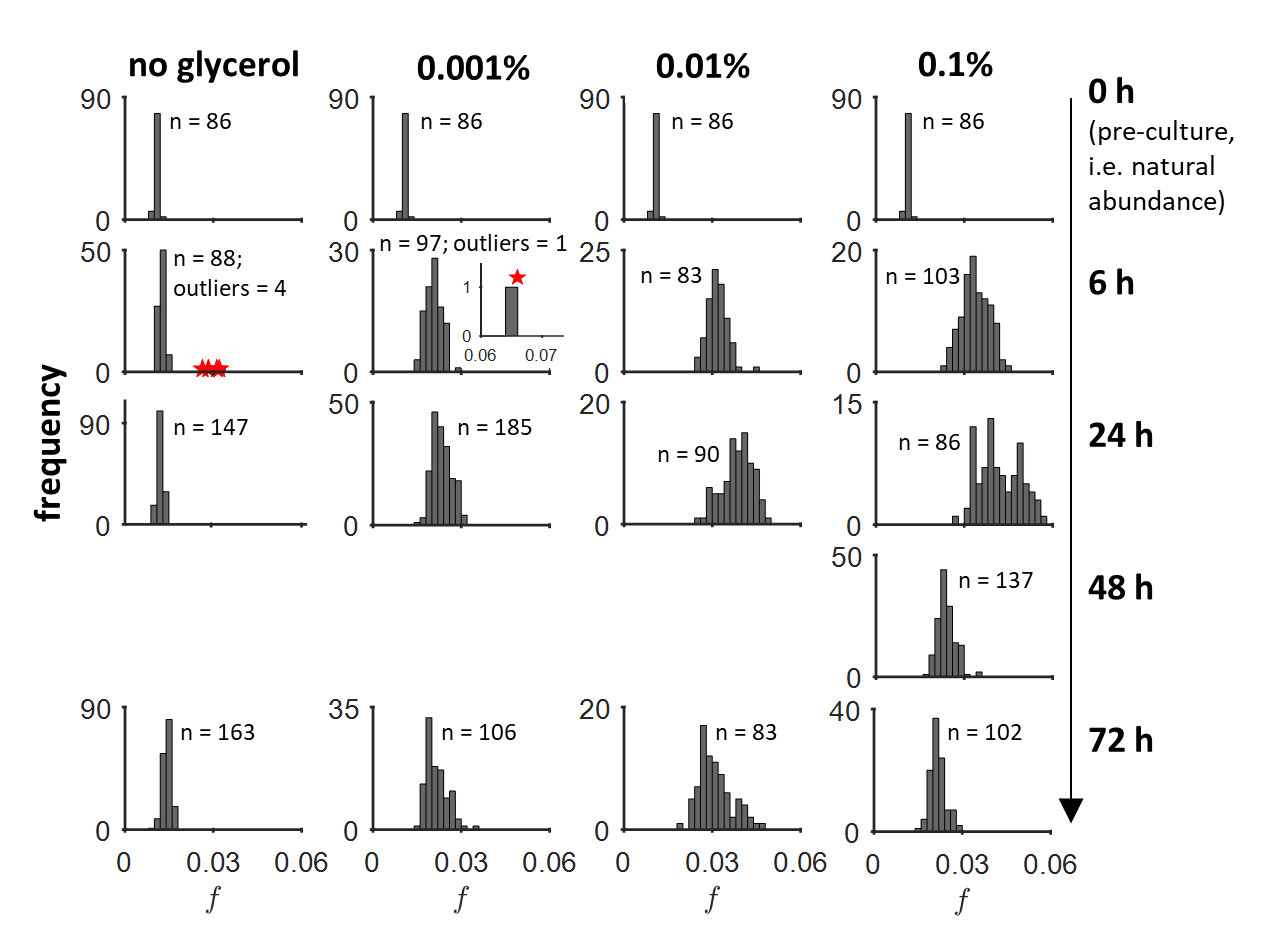

Supplement: S3 Fig — Histogram plots showing the dilution-corrected SIMS results for the single cell measurements of f. The number of cells (n) analysed and included in the calculation of the mean is indicated for each time point. The red stars indicate the points that were considered outliers and therefore excluded from the calculation of the mean. These small number of data points (i.e. 4 points for the 6 h sample from the no glycerol culture and 1 point for the 6 h sample from the 0.001% glycerol culture) with a relatively high atomic fraction of 13C could be the result of experimental artefacts like sub-resolution organic matter debris or cross-contamination between samples, see Supplementary Methods in S1 Text for details. Note that different scales have been used for the vertical axes. (TIF) [file pone.0251643.s003.tif]

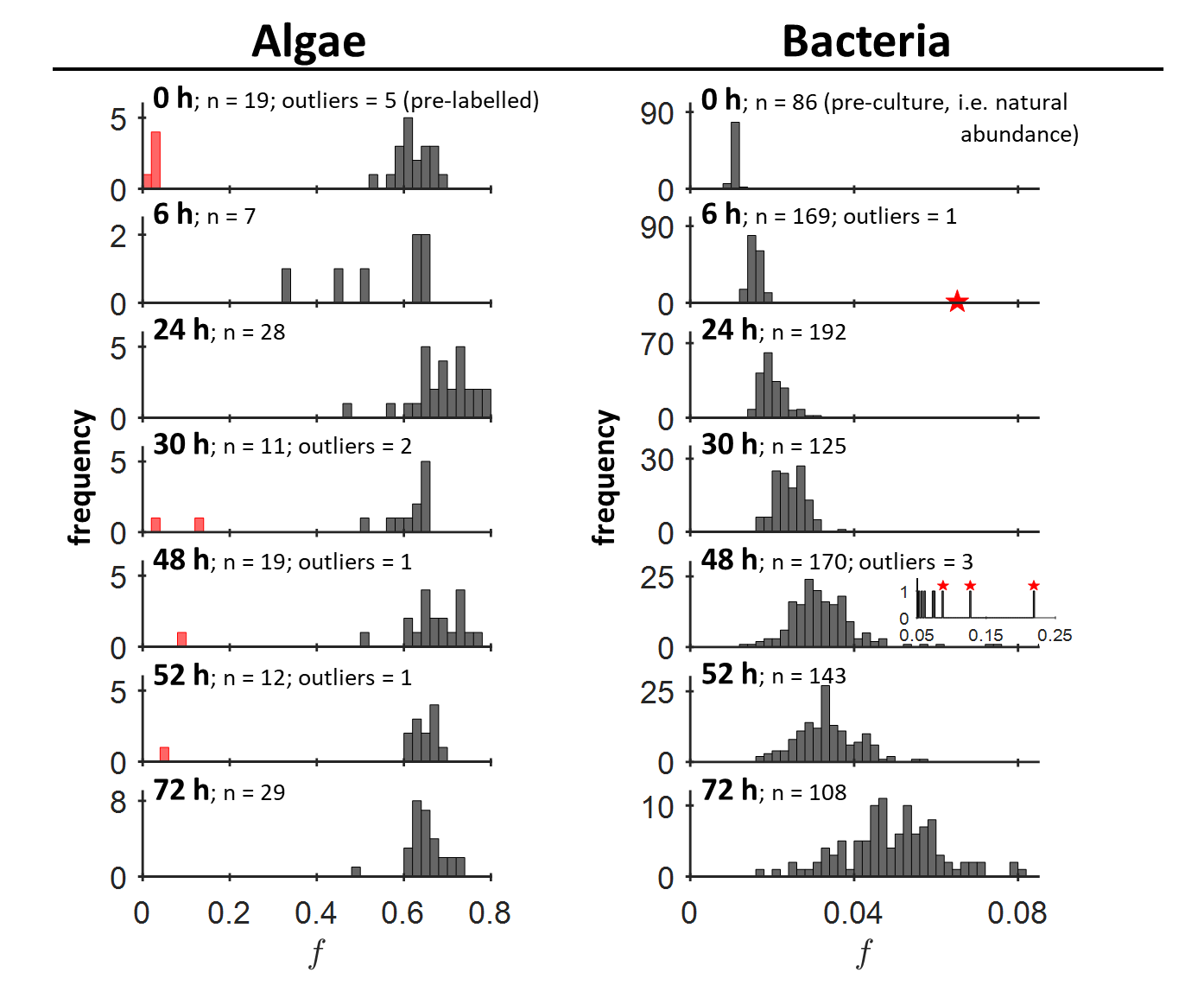

Supplement: S4 Fig — Histogram plots showing the dilution-corrected SIMS results for single cell measurements of the atomic fraction of 13C for algal and bacterial cells at different time-points of the co-culture. The number of cells (n) analysed and included in the calculation of the mean is indicated for each time point. The red bars indicate the algal cells that were considered ‘outliers’ and not included in the calculation of the mean because they were close to natural abundance and therefore considered inactive. The red stars indicate the of bacteria data points that were considered outliers and therefore excluded from the calculation of the mean. These outliers (i.e. 1 point for the 6 h sample and 3 points for the 48 h sample) could be the result of experimental artefacts like sub-resolution organic matter debris or cross-contamination between samples, see Supplementary Methods in S1 Text for details. Note that different scales have been used for the vertical axes. (TIF) [file pone.0251643.s004.tif]

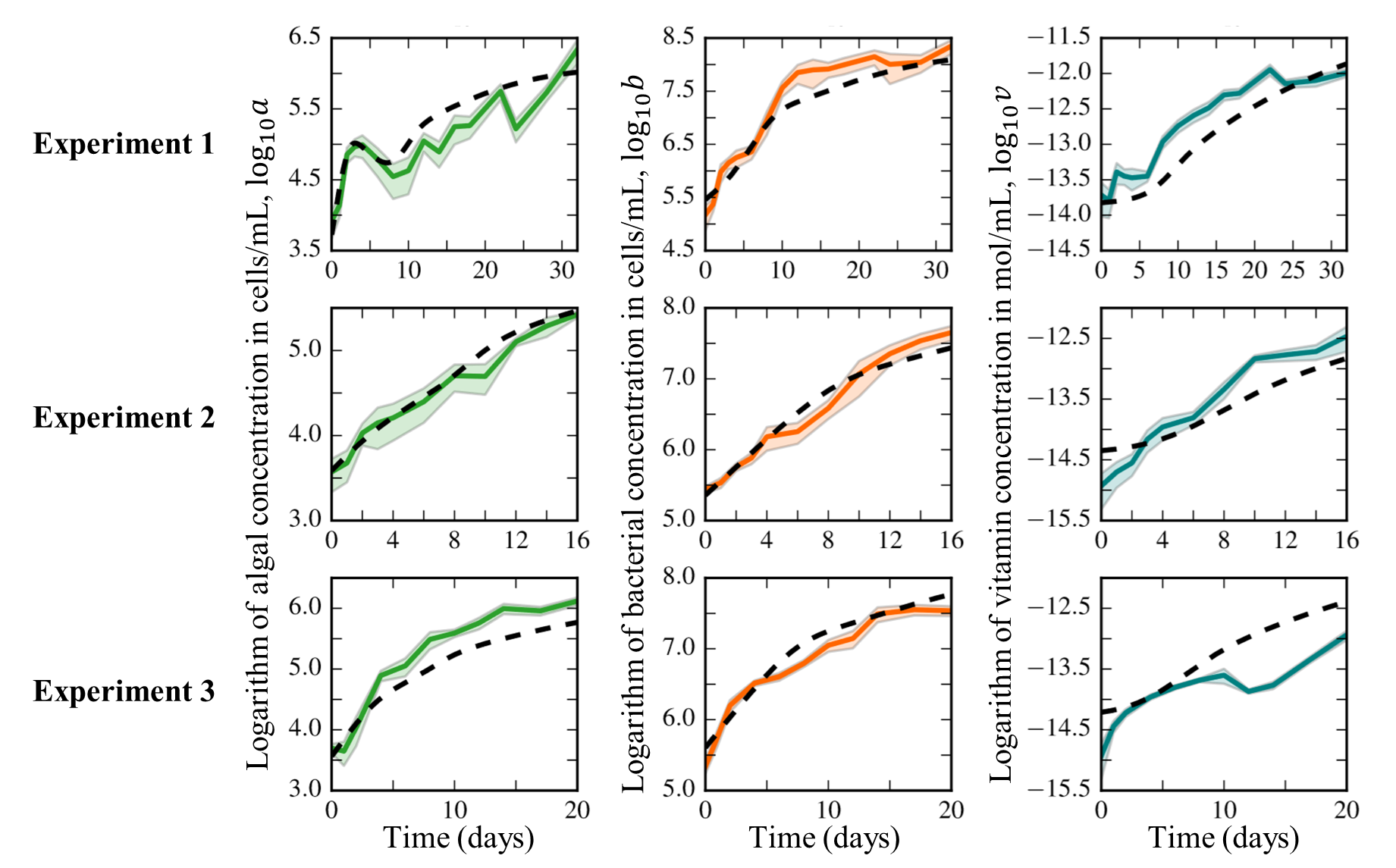

Supplement: S5 Fig — Fit of data obtained for co-cultures of C. reinhardtii metE7 and M. japonicum. Each row of plots corresponds to an independent experiment, with the first column the evolution of algal density, in the second column the evolution of bacterial density and in the third column the evolution of vitamin concentration determined by a bioassay, as described in [71]. The mean of each variable appears as a continuous line, with the shaded region showing the standard deviation for 8, 4 and 5 replicates for experiment 1, 2 and 3 respectively. The global fit with a unique set of parameters for the three independent experiments is shown in black dashed lines. The horizontal axes show time in days. (TIF) [file pone.0251643.s005.tif]

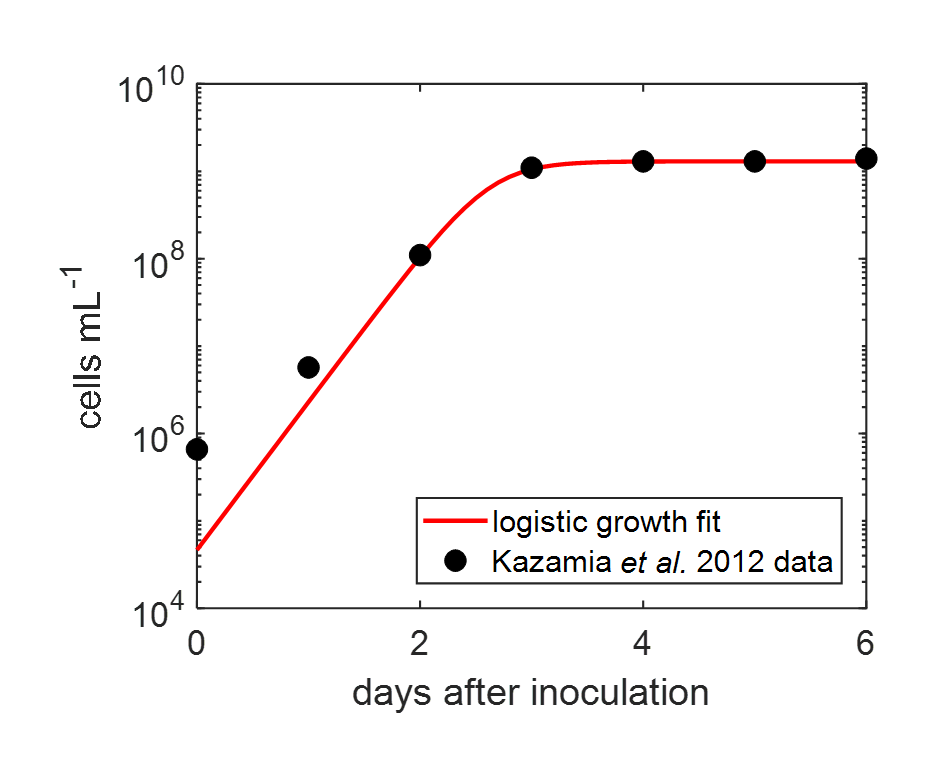

Supplement: S6 Fig — Data taken from [40] for M. japonicum grown axenically in 0.1% glycerol was fit with the logistic growth equation b = Kb/(1+M e−r t), with M = 2.8×10^4^, r = 3.9 and carrying capacity Kb = 1.3×10^9^. No error bars are shown because the fractional errors for the data points were within 20%, and appear smaller than a data point on the graph. (TIF) [file pone.0251643.s006.tif]

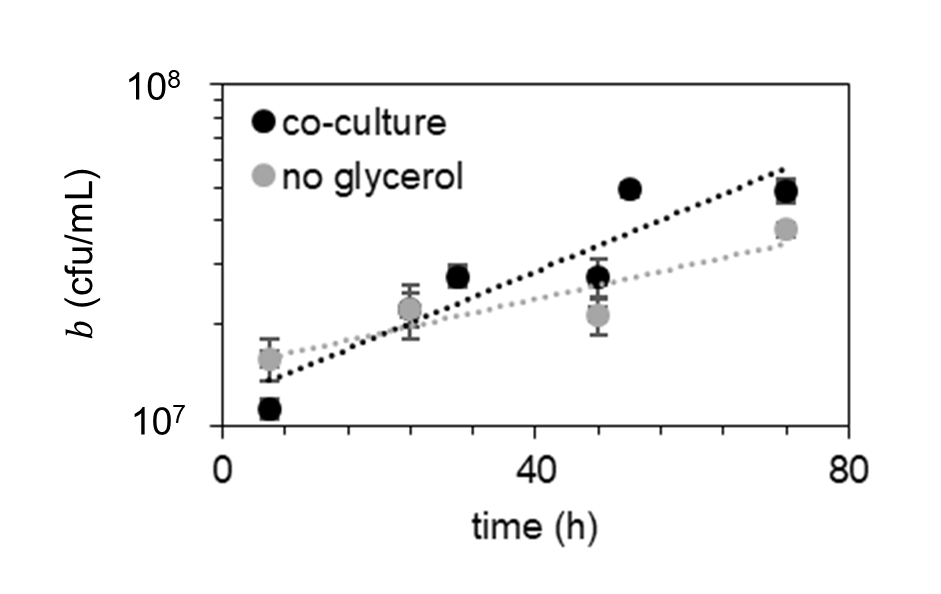

Supplement: S7 Fig — Plotted points show the viable count results for the growth of M. japonicum in the co-culture (black) and in the axenic culture grown without glycerol (grey). The dotted lines indicate the results for the exponential growth fit using equation b = b(0) exp(μB t), giving b(0) = 1.2±0.01×10^7^ cfu mL−1 and μB = 0.022±0.005 h−1 for the co-culture and b(0) = 1.5±0.01×10^7^ cfu mL−1 and μB = 0.012±0.004 h−1 for the axenic culture. (TIF) [file pone.0251643.s007.tif]

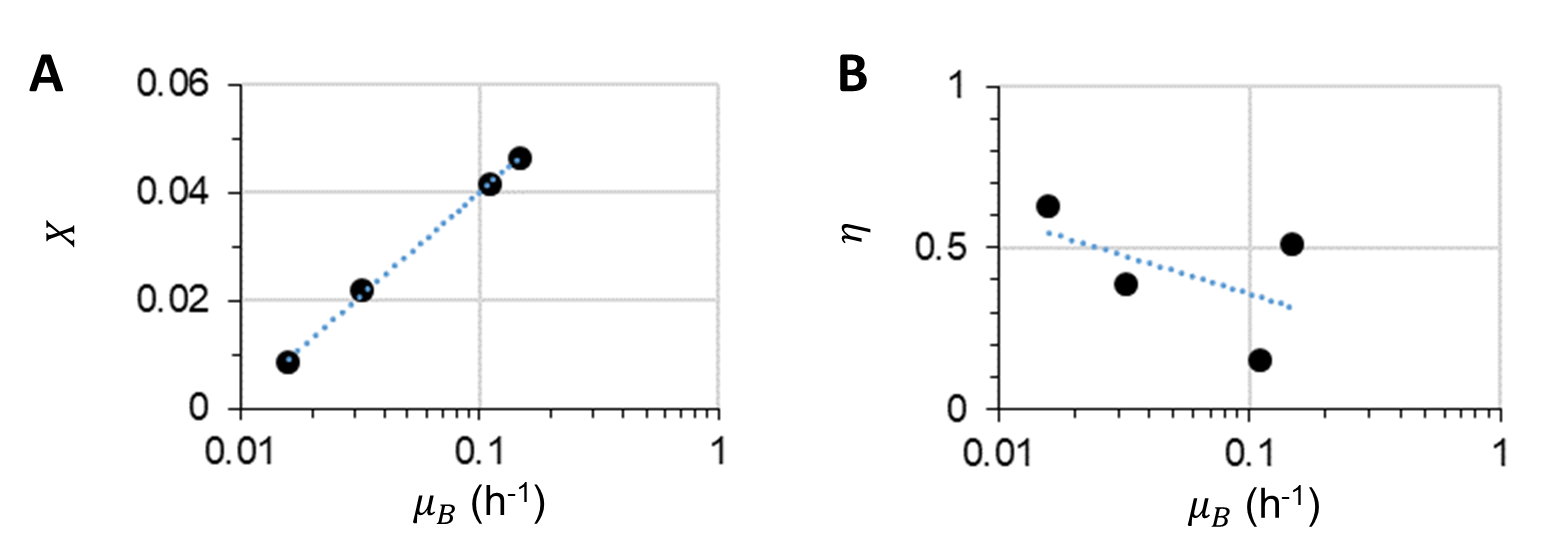

Supplement: S8 Fig — (A) The relationship between the DIC uptake parameter X and the initial exponential growth rate μB = μb co(0)/(co(0)+Kc) was approximated with a logarithmic fit using equation X = m ln(μB)+n, giving m = 0.0167±0.0004 and n = 0.0785±0.0013, with R2 = 0.999. (B) The relationship between the bacterial growth efficiency η and μB was approximated with a logarithmic fit using equation η = p ln(μB)+q, giving p = −0.10±0.12 and q = 0.12±0.36, with R2 = 0.282. (TIF) [file pone.0251643.s008.tif]

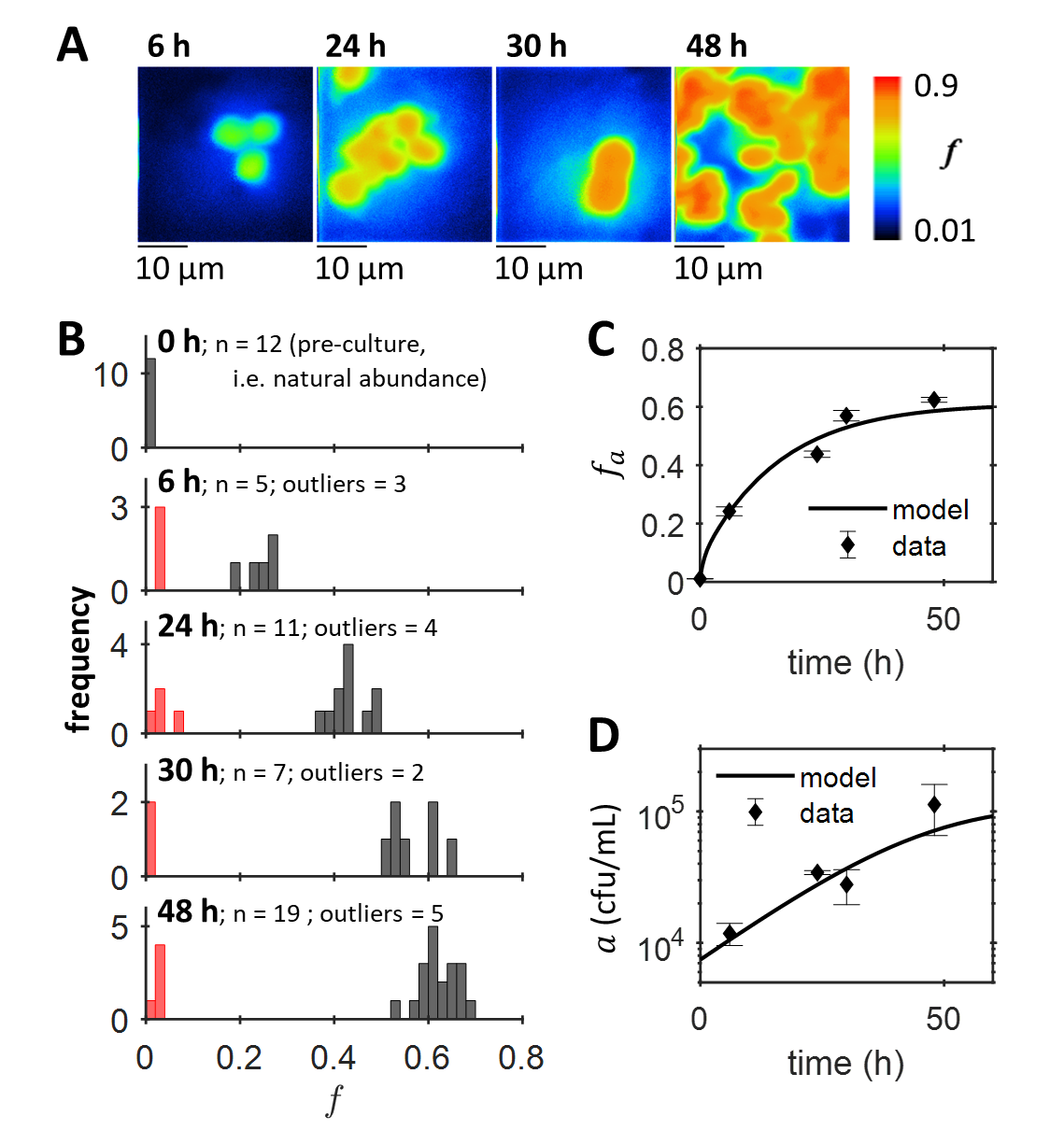

Supplement: S9 Fig — (A) Example images of the atomic fraction of 13C, f, obtained by SIMS analysis of algal cells sampled at different time-points of the pre-labelling, axenic culture grown with 5mM NaH13CO3. The colour map shows the scale, starting at natural abundance. (B) Histogram plots showing the dilution-corrected SIMS results for single cell measurements of the atomic fraction of 13C in individual algal cells. The number of cells (n) analysed and included in the calculation of the mean is indicated for each time point. The red bars indicate the algal cells that were considered ‘outliers’ and not included in the calculation of the mean because they were close to natural abundance and therefore considered inactive. Note that different scales have been used for the vertical axes. (C) The mean atomic fraction of 13C for the dilution-corrected SIMS measurements (diamonds). Error bars, showing the standard error, are small compared to the size of the plotted points. (D) Algal growth measured using viable counts (cfu mL−1), plotted on a logarithmic scale as the mean and standard error of two measurements (diamonds). The results of the model fit, with parameters and initial conditions as specified in Table 1, are also plotted for (C) fa and (D) a. (TIF) [file pone.0251643.s009.tif]

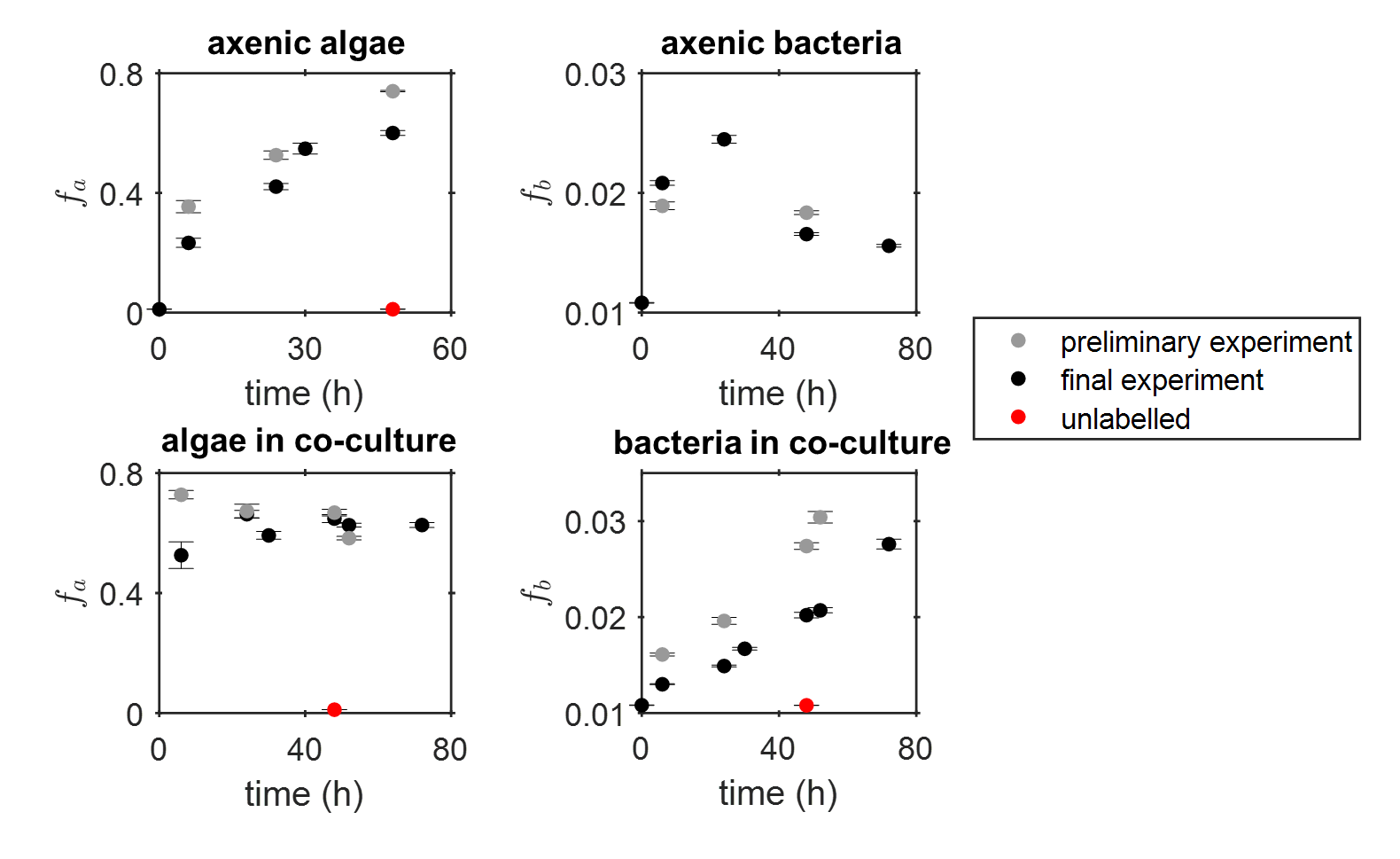

Supplement: S10 Fig — Preliminary SIMS results (grey circles) for the mean carbon isotope fraction are compared with the results from the final experiment (black circles) for the pre-labelling cultures of algae grown with 5 mM NaH13CO3, axenic cultures of bacteria grown with 0.1% glycerol and 5 mM NaH13CO3 and the algal-bacterial co-culture. Error bars correspond to the standard errors. In the preliminary experiment, control cultures of axenic algae and a co-culture were grown with 5 mM unlabeled NaHCO3 and a sample at 48 h was taken and analysed using SIMS to show that the cells remained at natural abundance (red circles). For algal cells, the preliminary experiment only obtained one SIMS measurement, whereas for the final experiment the values plotted represent the mean value for algal cells where the single cell values are the mean of 2–3 repeated SIMS measurements taken at the same location on the filter. The SIMS results presented here have not been dilution-corrected. Note that different scales have been used for the vertical axes. (TIF) [file pone.0251643.s010.tif]

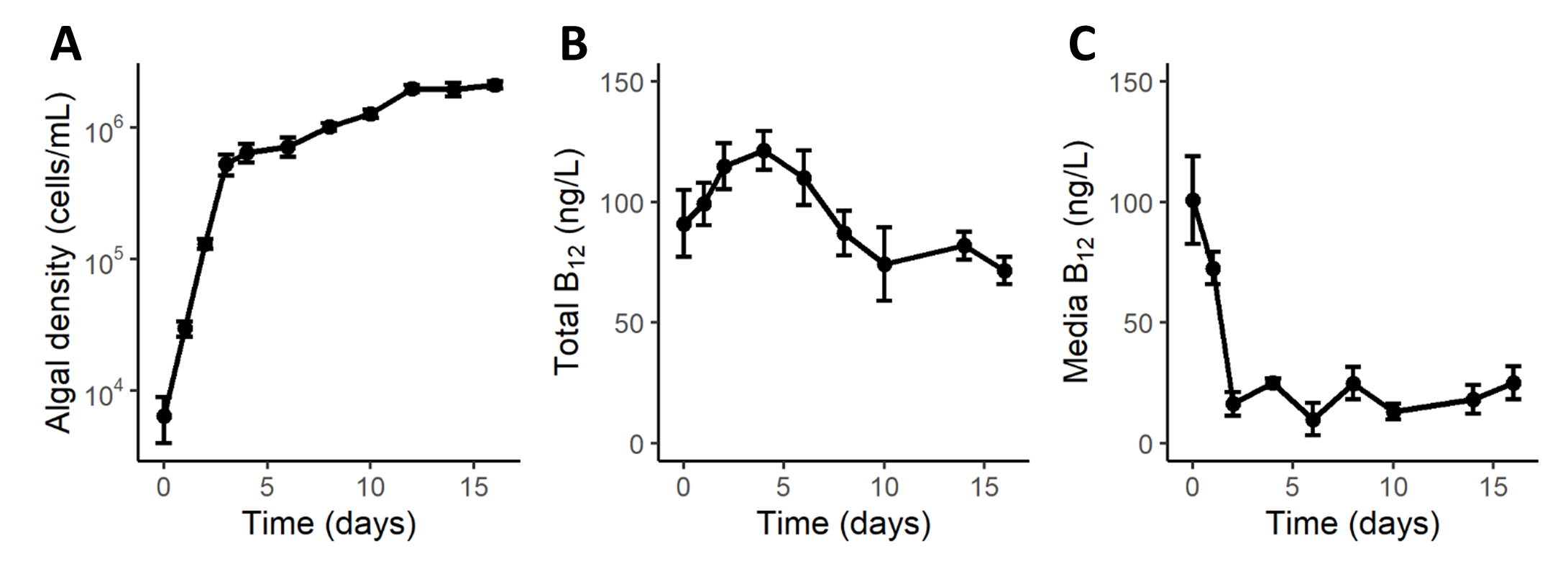

Supplement: S11 Fig — (A) Growth of C. reinhardtii metE7 in Tris minimal medium + 7.5×10^−14^ mol/mL B12 measured by counting cell density using a Z2 particle count analyser (Beckman Coulter Ltd). (B) Total B12 measured in the cells and media of the C. reinhardtii metE7 cultures. (C) B12 remaining in the media of the C. reinhardtii metE7 cultures. A bioassay, as described in [71], was used to quantify the B12 concentration, which measured the growth of a B12-dependent Salmonella typhimurium strain AR3612 when incubated with the sample of interest. Note that the B12 remaining in the media reaches a low point of roughly 1×10^−14^ mol/mL B12 after 2 days. Why this does not decrease to zero is unclear. Perhaps the affinity of the algal B12 uptake system is not high enough, or the bioassay overestimates B12, particularly when there is a lot of algal debris in the media. Error bars: standard deviation (n = 4). (TIF) [file pone.0251643.s011.tif]

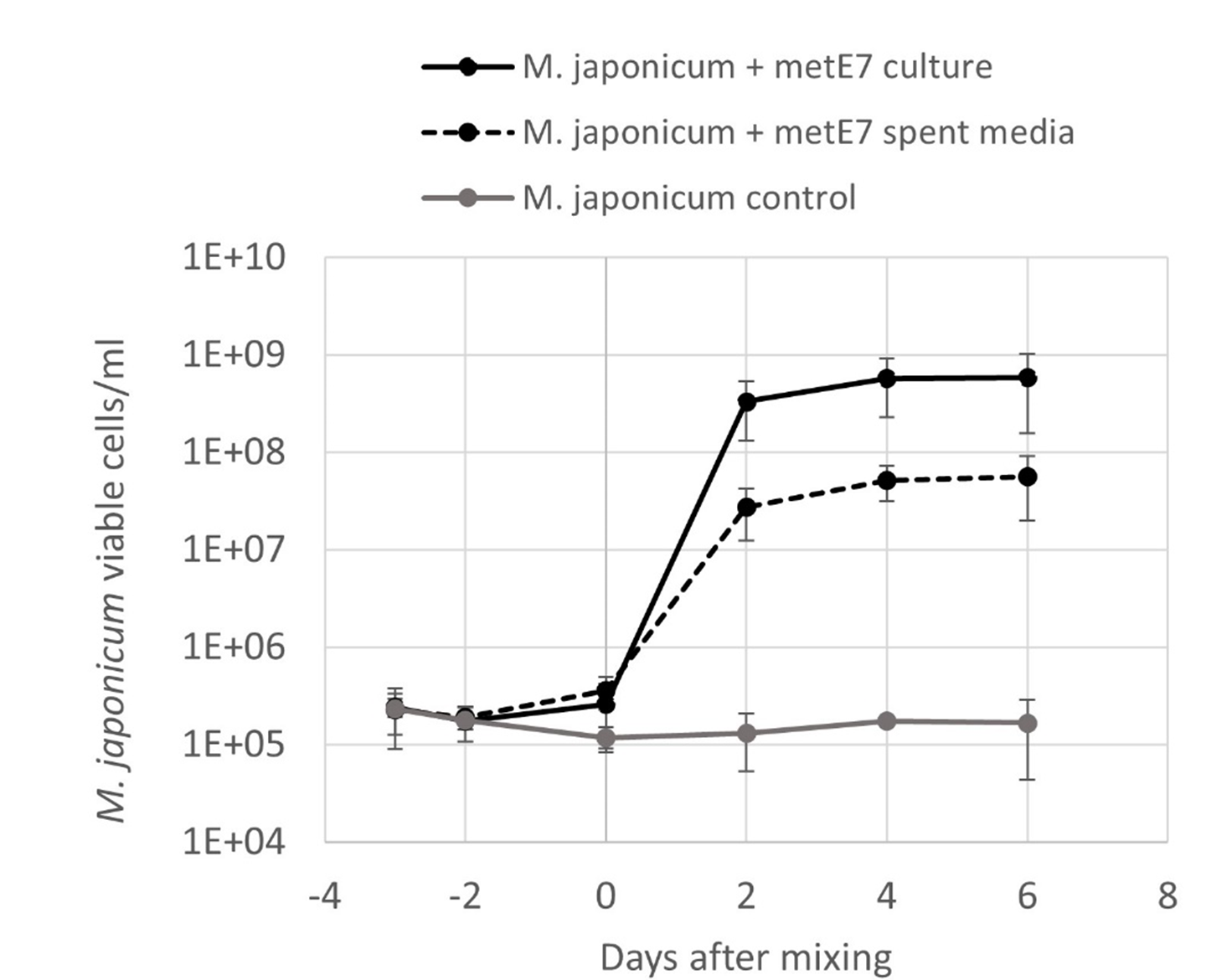

Supplement: S12 Fig — M. japonicum was cultured in 20 mL TP medium + 0.001% glycerol at 25°C and 12-hour light (100 μE·m-2·s-1) and 12-hour dark cycles with 120rpm rotational shaking. After M. japonicum cultures reached stationary phase, 2 mL of 1) stationary phase metE7 cultured in the same manner but with 200 ng·L-1 B12 instead of 0.001% glycerol was added to one set of M. japonicum cultures (black solid line), 2) the same metE7 culture filtered through a 0.4 μm filter (to remove the cells) was added to the second set (black dashed line), 3) TP medium was added to the third set (grey line). M. japonicum CFUs were measured before and after addition of these cultures. Error bars = standard deviation, n = 3. (TIF) [file pone.0251643.s012.tif]
