# Supplementary material for: Combining SIMS and mechanistic modelling to reveal nutrient kinetics in an algal-bacterial mutualism
Source: PLoS One. 2021 May 20;16(5):e0251643. doi: 10.1371/journal.pone.0251643 (PMC8136852; doi:10.1371/journal.pone.0251643)
Supplement: S1 Table — The concentrations of the different chemical components for each of the seven stock solutions of trace elements for the Tris-minimal media used in this work. For 1 L of Tris-minimal media, 1 mL of each solution was added. (DOCX) [file pone.0251643.s013.docx]

**Supplementary Table S1: Trace elements adapted from (Kropat et al. 2011)**. The concentrations of the different chemical components for each of the seven stock solutions of trace elements for the Tris-minimal media used in this work. For $1 L$ of Tris-minimal media, $1 mL$ of each solution was added.

| **Number** | **Chemical Component** | **Concentration (**$\boldsymbol{mM}$**)** |
| --- | --- | --- |
| 1 | $EDTA\cdot Na_{2}\cdot2H_{2}O$ | $25$ |
| 2 | $\left( NH_{4} \right)_{6}Mo_{7}O_{24}\cdot4H_{2}O$ | $0.032$ |
| 3 | $CuCl_{2}\cdot2H_{2}O$  $EDTA$ | $1.4$  $2$ |
| 4 | $ZnSO_{4}\cdot7H_{2}O$  $EDTA$ | $2.5$  $2.7$ |
| 5 | $MnCl_{2}\cdot4H_{2}O$  $EDTA$ | $6$  $6$ |
| 6 | $FeCl_{3}\cdot6H_{2}O$  $EDTA$  $Na_{2}CO_{3}$ | $20$  $22$  $22$ |
| 7 | $CoCl_{2}\cdot6H_{2}O$ | $7$ |
