# Supplementary material for: Combining SIMS and mechanistic modelling to reveal nutrient kinetics in an algal-bacterial mutualism
Source: PLoS One. 2021 May 20;16(5):e0251643. doi: 10.1371/journal.pone.0251643 (PMC8136852; doi:10.1371/journal.pone.0251643)
Supplement: S2 Table — A complete list of the cultures grown as part of the stable isotope labelling experiments described in this work. Tris-minimal growth medium was used for all cultures with the addition of B12, glycerol and sodium bicarbonate as listed in this table. These cultures were grown in 2 L conical flasks except for the pre-cultures, which were grown in 1 L flasks. Note that for B12 concentrations 1 ng/L = 7.5×10^−16^ mol/mL. (DOCX) [file pone.0251643.s014.docx]

**Supplementary Table S2: List of cultures.** A complete list of the cultures grown as part of the stable isotope labelling experiments described in this work. Tris-minimal growth medium was used for all cultures with the addition of B_12_, glycerol and sodium bicarbonate as listed in this table. These cultures were grown in $2 L$ conical flasks except for the pre-cultures, which were grown in $1 L$ flasks. Note that for B_12_ concentrations $1 ng/L=7.5\times{10}^{-16} mol/mL$.

| **Cultures for the preliminary experiment** | **Volume**  **(**$\boldsymbol{mL}$**)** | **B_12_**  **(**$\boldsymbol{ng/L}$**)** | **Glycerol**  **(**$\boldsymbol{\%v/v}$**)** | **Sodium bicarbonate** |
| --- | --- | --- | --- | --- |
| Algal pre-culture | $600$ | $1000$ |  |  |
| Axenic algae (pre-labelling) | $1000$ | $100$ |  | $5 mM NaH{}^{13}CO_{3}$ |
| Axenic algae (unlabelled) | $1000$ | $100$ |  | $5 mM NaHCO_{3}$ |
| Bacterial pre-culture | $400$ |  | $0.1$ |  |
| Axenic bacteria (0.1% glycerol) | $1000$ |  | $0.1$ | $5 mM NaH{}^{13}CO_{3}$ |
| Labelled co-culture | $1000$ |  |  | $5 mM NaH{}^{13}CO_{3}$ |
| Unlabelled co-culture | $1000$ |  |  | $5 mM NaHCO_{3}$ |
| **Cultures for the final experiment** | **Volume**  **(**$\boldsymbol{mL}$**)** | **B_12_**  **(**$\boldsymbol{ng/L}$**)** | **Glycerol**  **(**$\boldsymbol{\%v/v}$**)** | **Sodium bicarbonate** |
| Algal pre-culture | $600$ | $1000$ |  |  |
| Axenic algae (pre-labelling) | $1000$ | $100$ |  | $5 mM NaH{}^{13}CO_{3}$ |
| Bacterial pre-culture | $400$ |  | $0.1$ |  |
| Axenic bacteria (0.1% glycerol) | $1000$ |  | $0.1$ | $5 mM NaH{}^{13}CO_{3}$ |
| Axenic bacteria (0.01% glycerol) | $1000$ |  | $0.01$ | $5 mM NaH{}^{13}CO_{3}$ |
| Axenic bacteria (0.001% glycerol) | $1000$ |  | $0.001$ | $5 mM NaH{}^{13}CO_{3}$ |
| Axenic bacteria (no glycerol) | $1000$ |  |  | $5 mM NaH{}^{13}CO_{3}$ |
| Labelled co-culture | $1000$ |  |  | $5 mM NaH{}^{13}CO_{3}$ |
