# Supplementary material for: Combining SIMS and mechanistic modelling to reveal nutrient kinetics in an algal-bacterial mutualism
Source: PLoS One. 2021 May 20;16(5):e0251643. doi: 10.1371/journal.pone.0251643 (PMC8136852; doi:10.1371/journal.pone.0251643)
Supplement: S3 Table — The dilution factor, D, was obtained from a least squares fit of Eq (S4) in Supplementary Methods in S1 Text using the curve fitting application in Matlab and with fch = 0.0108. This table lists the results for D, the 95% confidence bounds, the number of points in the fit, n, and the least square displacements, R2. For bacteria, the fit was carried out using only data from axenic cultures. (DOCX) [file pone.0251643.s015.docx]

**Supplementary Table S3: The dilution factor results.** The dilution factor, $D$, was obtained from a least squares fit of equation (S4) in Supplementary Methods using the curve fitting application in Matlab and with $f_{ch}=0.0108$. This table lists the results for $D$, the$95 \%$confidence bounds, the number of points in the fit, $n$, and the least square displacements, $R^{2}$. For bacteria, the fit was carried out using only data from axenic cultures.

|  | $\boldsymbol{D}$ | $\boldsymbol{95\%}$ **confidence bound** | $\boldsymbol{n}$ | $\boldsymbol{R}^{\boldsymbol{2}}$ |
| --- | --- | --- | --- | --- |
| **Algae** | $0.04$ | $\boldsymbol{\pm}0.07$ | $8$ | $0.968$ |
| **Bacteria** | $1.29$ | $\boldsymbol{\pm}0.41$ | $9$ | $0.84$ |
