# Supplementary material for: Combining SIMS and mechanistic modelling to reveal nutrient kinetics in an algal-bacterial mutualism
Source: PLoS One. 2021 May 20;16(5):e0251643. doi: 10.1371/journal.pone.0251643 (PMC8136852; doi:10.1371/journal.pone.0251643)
Supplement: S4 Table — Summary of the EA-IRMS results for the carbon and nitrogen content and the carbon yield for the alga C. reinhardtii metE7 and bacterium M. japonicum. The table gives the mean, standard error and the number of samples included in the mean (n). (DOCX) [file pone.0251643.s016.docx]

**Supplementary Table S4: C-N content and carbon yield (**$\boldsymbol{Y}_{\boldsymbol{a,c}}$ **and** $\boldsymbol{Y}_{\boldsymbol{b,c}}$ **for algae and bacteria respectively).** Summary of the EA-IRMS results for the carbon and nitrogen content and the carbon yield for the alga *C. reinhardtii* metE7 and bacterium *M. japonicum*. The table gives the mean, standard error and the number of samples included in the mean ($n$).

|  |  | **Mean** | **Standard error** | $\boldsymbol{n}$ |
| --- | --- | --- | --- | --- |
| **Algae** | $\%AmtC$ | $35$ | $4$ | $8$ |
|  | $\%AmtN$ | $8$ | $1$ | $8$ |
|  | $C:N$ ratio | $4.4$ | $0.1$ | $8$ |
|  | $Y_{a,c}$ ($cells molC^{-1}$) | $4\times{10}^{12}$ | $1\times{10}^{12}$ | $4$ |
| **Bacteria** | $\%AmtC$ | $39$ | $2$ | $13$ |
|  | $\%AmtN$ | $11$ | $1$ | $13$ |
|  | $C:N$ ratio | $3.71$ | $0.03$ | $13$ |
|  | $Y_{b,c}$ ($cfu molC^{-1}$) | $5\times{10}^{14}$ | $1\times{10}^{14}$ | $10$ |
