# Supplementary material for: Combining SIMS and mechanistic modelling to reveal nutrient kinetics in an algal-bacterial mutualism
Source: PLoS One. 2021 May 20;16(5):e0251643. doi: 10.1371/journal.pone.0251643 (PMC8136852; doi:10.1371/journal.pone.0251643)
Supplement: S5 Table — These were the fitting intervals used for the various free parameters and free initial conditions of the parameter optimisations run for axenic algae, axenic bacteria and the co-culture. When units are not specified the parameter/initial condition is in dimensionless units. The fitting intervals for ϕs, η and X came from their definition requiring these parameters to be between 0 and 1. Other fitting intervals were chosen to ensure that the parameter optimisation results were reasonable when considering their biological interpretation. In particular, the choice of fitting intervals was informed by the physiologically relevant parameter ranges for the mutualistic association of M. japonicum and L. rostrata provided by (Peaudecerf et al. 2018). (DOCX) [file pone.0251643.s017.docx]

**Supplementary Table S5: Fitting intervals for the parameter optimisations.** These were the fitting intervals used for the various free parameters and free initial conditions of the parameter optimisations run for axenic algae, axenic bacteria and the co-culture. When units are not specified the parameter/initial condition is in dimensionless units. The fitting intervals for $\phi_{s}$, $\eta$ and $X$ came from their definition requiring these parameters to be between 0 and 1. Other fitting intervals were chosen to ensure that the parameter optimisation results were reasonable when considering their biological interpretation. In particular, the choice of fitting intervals was informed by the physiologically relevant parameter ranges for the mutualistic association of *M. japonicum* and *L. rostrata* provided by (Peaudecerf et al. 2018).

|  | **Fitting Interval** | **Units** |
| --- | --- | --- |
| All parameter optimisations: | $0\leq\varphi_{s}\leq0.99$ |  |
|  | $0.01\leq\eta\leq1$ |  |
|  | $0\leq X\leq1$ |  |
| Axenic algae: | $0\leq s_{c}\leq10$ |  |
|  | $0\leq\hat{v}(0)\leq5$ |  |
|  | $0.001\leq\hat{a}(0)\leq0.01$ |  |
|  | $0\leq f_{i}(0)\leq1$ |  |
| Axenic bacteria: | $5\times{10}^{6}\leq b\left( 0 \right)\leq5\times{10}^{7}$ | $cells mL^{-1}$ |
|  | $0.01\leq\mu_{b}\leq2$ | $h^{-1}$ |
|  | $1\times{10}^{-10}\leq K_{c}\leq1\times{10}^{-4}$ | $molC mL^{-1}$ |
| Axenic bacteria, no glycerol: | $0\leq c_{o}\left( 0 \right)\leq4\times{10}^{-7}$ | $molC mL^{-1}$ |
| Co-culture: | $0\leq s_{c}\leq10$ |  |
|  | $0.001\leq\hat{a}(0)\leq0.01$ |  |
|  | $0.001\leq\hat{b}(0)\leq0.03$ |  |
|  | $1\times{10}^{-5}\leq\hat{c}_{o}(0)\leq0.5$ |  |
|  | $0.0108\leq f_{o}(0)\leq1$ |  |
