# Supplementary material for: Combining SIMS and mechanistic modelling to reveal nutrient kinetics in an algal-bacterial mutualism
Source: PLoS One. 2021 May 20;16(5):e0251643. doi: 10.1371/journal.pone.0251643 (PMC8136852; doi:10.1371/journal.pone.0251643)
Supplement: S6 Table — The non-dimensional model parameter definitions and estimated values for C. reinhardtii metE7 and M. japonicum grown both axenically and in co-culture. (DOCX) [file pone.0251643.s018.docx]

**Supplementary Table S6: Non-dimensional model parameters.** The non-dimensional model parameter definitions and estimated values for *C. reinhardtii* metE7 and *M. japonicum* grown both axenically and in co-culture.

| **Non-dimensional parameter** | **Symbol** | **Definition** | **Axenic algae** | **Axenic bacteria** | **Co-culture** |
| --- | --- | --- | --- | --- | --- |
| Ratio of maximum growth rates | $\varepsilon$ | $=\mu_{a}/\mu_{b}$ | $0.51$ [a] | - | $0.51$ [a] |
| Algal B12 uptake parameter | $k_{a,v}$ | $=K_{a}/(K_{v} Y_{a,v})$ | $7.8$ [a] | - | $7.8$ [a] |
| Algal carbon uptake parameter | $k_{a,c}$ | $=K_{a}/(K_{c} Y_{a,c})$ | $0.91$ [b] | - | $0.91$ [b] |
| Bacterial carbon uptake parameter | $k_{b,c}$ | $=K_{b}/(K_{c} Y_{b,c})$ | - | $1.73$ [c] | $3.6$ [a] |
| B12 production strength | $s_{v}$ | $=(p_{v} K_{b})/(\mu_{a} K_{v})$ | - | $4.2$ [a] | $4.2$ [a] |
| DOC production strength | $s_{c}$ | $=(p_{c} K_{a})/(\mu_{b} K_{c})$ | $2.13$ [d] | - | $0.047$ [e] |

[a] From fitting a simplified co-culture model (i.e. $\phi_{s}=0$, $\eta'=1$ and $X=0$) to population growth and B_12_ concentration data, see Supplementary Methods for details.

[b] From the definition $k_{a,c}=\frac{K_{a}}{K_{c} Y_{a,c}}.$

[c] From the definition $k_{b,c}=\frac{K_{b}}{K_{c} Y_{b,c}}$.

[d] Parameter optimisation results from fitting the model to the axenic, pre-labelling culture of *C. reinhardtii* metE7, see Supplementary Methods for details. The residual sum of squares for this global parameter optimisation result was 0.313. For comparison, when storage was not included in the model (i.e. $\phi_{s}=0$), the parameter optimisation result gave $s_{c}=0.041$ and the residual sum of squares was 0.323.

[e] Parameter optimisation results from fitting the model to co-culture growth and SIMS data, i.e. fit 1 in Supplementary Table S8, see Supplementary Methods for further details.
