# Supplementary material for: Combining SIMS and mechanistic modelling to reveal nutrient kinetics in an algal-bacterial mutualism
Source: PLoS One. 2021 May 20;16(5):e0251643. doi: 10.1371/journal.pone.0251643 (PMC8136852; doi:10.1371/journal.pone.0251643)
Supplement: S7 Table — Model parameter values for the axenic cultures of M. japonicum grown with different concentrations of glycerol determined by a global parameter optimisation performed for the four axenic cultures of M. japonicum grown with 0.1%, 0.01%, 0.001% and no glycerol. The global free parameters were μb and Kc, which were constrained to have the same value for all four cultures. The free parameters and initial conditions that were permitted to be different for the different cultures were η, X and b(0). The initial DOC concentration co(0) for the culture grown without glycerol was also included as a free parameter. The fixed initial conditions were c^i(0)=5,v^(0)=0, fb(0) = 0.0108, fo(0) = 0.0108, and fi(0) = 0.65, since for the experiments it was assumed that the DIC was in excess, initially there was no B12 and the bacteria had natural abundance, the glycerol was unlabelled and the atomic fraction of 13C in the DIC was taken as the estimate obtained from the parameter optimisation for axenic algae (see Table 1). The residual sum of squares for this global parameter optimisation result was 0.58, whereas when respiration was not included in the model it was 2.24. (DOCX) [file pone.0251643.s019.docx]

**Supplementary Table S7: Culture specific model parameters and initial conditions for axenic bacteria.** Model parameter values for the axenic cultures of *M. japonicum* grown with different concentrations of glycerol determined by a global parameter optimisation performed for the four axenic cultures of *M. japonicum* grown with $0.1 \%$, $0.01 \%$, $0.001 \%$ and no glycerol. The global free parameters were $\mu_{b}$ and $K_{c}$, which were constrained to have the same value for all four cultures. The free parameters and initial conditions that were permitted to be different for the different cultures were $\eta$, $X$ and $b\left( 0 \right)$. The initial DOC concentration $c_{o}\left( 0 \right)$ for the culture grown without glycerol was also included as a free parameter. The fixed initial conditions were $\hat{c}_{i}\left( 0 \right)=5$, $\hat{v}\left( 0 \right)=0$, $f_{b}\left( 0 \right)=0.0108$, $f_{o}\left( 0 \right)=0.0108$ and $f_{i}\left( 0 \right)=0.65$, since for the experiments it was assumed that the DIC was in excess, initially there was no B_12_ and the bacteria had natural abundance, the glycerol was unlabelled and the atomic fraction of ${}^{13}C$ in the DIC was taken as the estimate obtained from the parameter optimisation for axenic algae (see Table 1). The residual sum of squares for this global parameter optimisation result was $0.58$, whereas when respiration was not included in the model it was $2.24$.

| **Culture** | $\boldsymbol{c}_{\boldsymbol{o}}\left( \boldsymbol{0} \right)$  **(**$\boldsymbol{molC m}\boldsymbol{L}^{\boldsymbol{-1}}$**)** | $\boldsymbol{b}\left( \boldsymbol{0} \right)$  **(**$\boldsymbol{cells m}\boldsymbol{L}^{\boldsymbol{-1}}$**)** | $\boldsymbol{\eta}$ | $\boldsymbol{X}$ |
| --- | --- | --- | --- | --- |
| $0.1 \%$ glycerol | $4\times{10}^{-5}$ [a] | $8.8\times{10}^{6}$ | $0.51$ | $0.046$ |
| $0.01 \%$ glycerol | $4\times{10}^{-6}$ [a] | $1.6\times{10}^{7}$ | $0.15$ | $0.042$ |
| $0.001 \%$ glycerol | $4\times{10}^{-7}$ [a] | $1.8\times{10}^{7}$ | $0.39$ | $0.022$ |
| no glycerol | $1.7\times{10}^{-7}$ | $1.3\times{10}^{7}$ | $0.63$ | $0.009$ |

[a] Not free in the parameter optimisation, calculated from the $\%$ glycerol concentration using the molar mass of glycerol, $92.09 g mol^{-1}$, and its density, $1.26 g mol^{-1}$.
