# Supplementary material for: Combining SIMS and mechanistic modelling to reveal nutrient kinetics in an algal-bacterial mutualism
Source: PLoS One. 2021 May 20;16(5):e0251643. doi: 10.1371/journal.pone.0251643 (PMC8136852; doi:10.1371/journal.pone.0251643)
Supplement: S8 Table — Results of different parameter optimisation results for the co-culture between C. reinhardtii metE7 and M. japonicum. The only free parameter was sc and the free initial conditions were a^(0),b^(0) and c^o(0), with f^o(0) included as an additional free initial condition for fit 2. The parameters ϕs = 0.9, η = 0.51 and X = 0.015 were estimated using results from axenic cultures as specified in the text. All other parameters had values as specified in Table 1. The fixed initial conditions were c^i(0)=5 (i.e. DIC concentration in excess), v^(0)=0 (i.e. initially no B12 in the media), fa(0) = 0.59 and fa,p(0) = 0.65 (i.e. using model results for the pre-labelling, axenic culture of algae, see text for details), fb(0) = 0.0108 (i.e. bacteria initially have natural abundance), and fi(0) = 0.65 (i.e. from the parameter optimisation result for axenic algae, see Table 1). (DOCX) [file pone.0251643.s020.docx]

**Supplementary Table S8: Comparison of parameter optimisation results for the algal-bacterial co-culture.** Results of different parameter optimisation results for the co-culture between *C. reinhardtii* metE7 and *M. japonicum*. The only free parameter was $s_{c}$ and the free initial conditions were $\hat{a}\left( 0 \right)$, $\hat{b}\left( 0 \right)$ and $\hat{c}_{o}\left( 0 \right)$, with $\hat{f}_{o}\left( 0 \right)$ included as an additional free initial condition for fit 2. The parameters $\phi_{s}=0.9$, $\eta=0.51$ and $X=0.015$ were estimated using results from axenic cultures as specified in the text. All other parameters had values as specified in Table 1. The fixed initial conditions were $\hat{c}_{i}\left( 0 \right)=5$ (i.e. DIC concentration in excess), $\hat{v}\left( 0 \right)=0$ (i.e. initially no B_12_ in the media), $f_{a}\left( 0 \right)=0.59$ and $f_{a,p}\left( 0 \right)=0.65$ (i.e. using model results for the pre-labelling, axenic culture of algae, see text for details), $f_{b}\left( 0 \right)=0.0108$ (i.e. bacteria initially have natural abundance), and $f_{i}\left( 0 \right)=0.65$ (i.e. from the parameter optimisation result for axenic algae, see Table 1).

| **Fit** | $\boldsymbol{s}_{\boldsymbol{c}}$  **DOC export parameter** | $\hat{\boldsymbol{a}}\left( \boldsymbol{0} \right)$  **Initial algal cell density** | $\hat{\boldsymbol{b}}\left( \boldsymbol{0} \right)$  **Initial bacterial cell density** | ${\hat{\boldsymbol{c}}}_{\boldsymbol{o}}\left( \boldsymbol{0} \right)$  **Initial DOC concentration** | $\boldsymbol{f}_{\boldsymbol{o}}\left( \boldsymbol{0} \right)$  **Initial DOC atomic fraction** | $\boldsymbol{r}^{\boldsymbol{2}}$  **Residual sum of squares** |
| --- | --- | --- | --- | --- | --- | --- |
| 1 [a] | $0.047$ | $0.005$ | $0.017$ | $0.0014$ | $0.64$ [a] | 1.96 |
| 2 [b] | $0.074$ | $0.005$ | $0.009$ | $0.13$ | $0.0144$ | 1.29 |

[a] Initial atomic fraction of ${}^{13}C$ for the DOC, $f_{o}\left( 0 \right)=0.64$, estimate obtained using the parameter optimisation result for axenic algae.

[b] Initial atomic fraction of ${}^{13}C$ for the DOC included as a free parameter.
