# Supplementary material for: Combining SIMS and mechanistic modelling to reveal nutrient kinetics in an algal-bacterial mutualism
Source: PLoS One. 2021 May 20;16(5):e0251643. doi: 10.1371/journal.pone.0251643 (PMC8136852; doi:10.1371/journal.pone.0251643)
Supplement: S1 Text — (DOCX) [file pone.0251643.s021.docx]

**SUPPORTING INFORMATION**

Title: Combining SIMS and mechanistic modelling to reveal nutrient kinetics in an algal-bacterial mutualism

Hannah Laeverenz Schlogelhofer [1] ^[[1]](#footnote-1)^, François J. Peaudecerf [2] ^[[2]](#footnote-2)^, Freddy Bunbury [3] ^[[3]](#footnote-3)^, Martin J. Whitehouse [4], Rachel A. Foster [5], Alison G. Smith [3] and Ottavio A. Croze [1]

[1] Cavendish Laboratory, University of Cambridge, United Kingdom; [2] Department of Applied Mathematics and Theoretical Physics, University of Cambridge, United Kingdom; [3] Department of Plant Sciences, University of Cambridge, United Kingdom; [4] Swedish Museum of Natural History, Stockholm, Sweden; [5] Department of Ecology, Environment and Plant Sciences, Stockholm University, Sweden

*Corresponding author:

Email: [o.croze@physics.org](mailto:o.croze@physics.org)

**Short title:** SIMS-modelling approach to microbial interactions

1. Supplementary Methods
2. Supplementary Results

I. Supplementary Methods

# EA-IRMS analysis and estimating the algal and bacterial carbon yield

## Sample preparation and analysis

To prepare samples for EA-IRMS analysis, first the biomass was concentrated using centrifugation. For co-cultures, an additional slow centrifugation step was used to concentrate the algal cells as a pellet, the supernatant was then passed through a $3 \mu m$ filter and the filtrate was used as the bacterial fraction of the co-culture biomass, which was concentrated into a pellet by centrifugation. The concentrated biomass samples were transferred to eppendorfs and dried overnight in an oven at $50^{\circ}C$. To remove any excess $NaH{}^{13}CO_{3}$ the samples were placed in a desiccator with $32 \% HCl$ for acid fumigation. The dry mass of the samples was measured and the required amount for EA-IRMS analysis was weighed out and encapsulated in tin. It was not possible to collect enough dry mass for EA-IRMS analysis at every time-point. For samples with enough dry mass, $1$ to $4$ sub-samples were analysed at the Godwin lab, Department of Earth Sciences, University of Cambridge using the Thermo Delta V Plus and Costech equipment.

## Estimating the algal and bacterial carbon yield

The carbon and nitrogen content of *M. japonicum* and *C. reinhardtii* metE7 were obtained from EA-IRMS analysis, the results are given in Table S4. To calculate the carbon yield for algal cells (i.e. $Y_{a,c}$, the number of cells per mole of carbon) we used the equation

$Y_{a,c}=M_{r}\frac{a\cdot V\cdot100}{m\cdot\%\mathrm{AmtC}}$ , (S1)

with $a$ the algal cell density in $cells mL^{-1}$ measured using a Coulter counter, $V$ the sample volume in $mL$, $m$the sample dry mass in $g$, $\%\text{AmtC}$ the percent of dry mass that is carbon and $M_{r}$ the molar mass of carbon in $g mol^{-1}$. The value for the molar mass depends on the atomic fraction of ${}^{13}C$ and therefore $M_{r}$ was calculated using

$M_{r}=12\cdot\left( 1-f \right)+13\cdot f$ , (S2)

with $f$ the atomic fraction of ${}^{13}C$ obtained from EA-IRMS analysis. There were four algal samples that had suitable dry mass and EA-IRMS measurements to be able to estimate the carbon yield. From these four estimates the carbon yield for algal cells was found to be $4\pm1\times{10}^{12} cells molC^{-1}.$ The carbon yield for bacterial cells, $Y_{b,c}$, was calculated in the same way, but using the viable count measurement of cell density in $cfu mL^{-1}$. There were ten bacterial samples that had suitable dry mass and EA-IRMS measurements to be able to estimate the carbon yield. From these ten estimates the carbon yield for bacterial cells was found to be $5\pm1\times{10}^{14} cells molC^{-1}$.

# SIMS technical details

## Sample preparation

***Chemical fixation with formaldehyde***. For every $10 mL$ of sample volume, $0.54 mL$ of $37-41 \% (w/v)$ formaldehyde was added to reach a final formaldehyde concentration of about $2 \% (w/v)$. The sample was gently vortexed and then incubated at $4-6^{\circ}C$ for $1 h$. To remove the fixative, the sample was washed twice by centrifugation followed by re-suspension in $1$X PBS buffer (i.e. phosphate buffered saline solution consisting of $10 mM Na_{2}HPO_{4}$ and $150 mM NaCl$). The sample was then centrifuged for a third time and finally re-suspended in a $1:1$ by volume mix of $1$X PBS buffer and $96 \%$ ethanol solution. Samples were stored in the fridge ($4-6^{\circ}C$) until further use.

***Cell staining and vacuum filtration.*** In order to be able to visualise the distribution of algal and bacterial cells on the membrane filter, SYTO9 green fluorescent nucleic acid stain (taken from a Molecular Probes LIVE/DEAD BacLight bacterial viability kit) was used for both bacterial and algal cells. Per $1 mL$ of sample, $1.5 \mu L$ of $3.34 mM$ SYTO9 was added, the sample was then incubated in the dark and at room temperature for $15$ minutes. An appropriate sample volume was chosen for vacuum filtration in order to achieve an even distribution of cells on the filter, which meant choosing a volume that contained $0.5\times{10}^{5}$ to $2\times{10}^{5} cfu$ for algae and $1\times{10}^{7}$ to $1\times{10}^{8} cfu$ for bacteria. Isopore membrane filters with a pore size of $0.22 \mu m$ and diameter $25 mm$ (Merck Millipore) were pre-sputtered with $\approx20 nm$ gold coating, using a BioRad SEM Coating System, and cells were then deposited on these gold-coated filters by vacuum filtration using a Charles Austen Capex 8C vacuum pump.

***Confocal microscopy.*** It is important that samples prepared for SIMS are flat, as an uneven sample can result in unreliable measurements (Watrous et al. 2011). An Olympus Fluoview laser scanning confocal microscope (FV1200) was used to image the filter samples and to ensure an even distribution of cells. A $473 nm$ excitation laser was used and fluorescence emission was detected in two channels; $490-525 nm$ to detect the green fluorescence of the SYTO9 nucleic acid stain and $560-660 nm$ to detect the chlorophyll autofluorescence of algae. The microscope images showed that an even distribution of algal and bacterial cells was achieved across the filter in a relatively uniform layer. The orthogonal views obtained from a series of z-stack images (with a $2 \mu m$ step size) confirmed that the vacuum filtration achieved an approximate monolayer of cells.

***Laser marking and gold coating.*** A single hole punch was used to cut out $4-6 mm$ disks from the filter samples. Following this, a Zeiss laser micro-dissection microscope (Zeiss LSM710-NLO housed at the LCI facility of the Karolinska Institute, Stockholm) was used to laser-mark the filter samples and to image the autofluorescence of the algal chlorophyll using the FITC and Rhodamine filter sets. The laser markings could be seen with the camera of the SIMS instrument, and so the SIMS measurements could be matched to chosen sample areas corresponding to particular algal cells in the fluorescence images. After laser-marking the filter samples, they were placed on a conductive sticky tape and mounted onto a glass disk to be placed in the sample holder of the SIMS instrument. The samples were then sputter coated with gold at the NordSIM facility to ensure conductivity of the sample.

## Data analysis using WinImage

The WinImage2 software (CAMECA) was used to calculate the isotope ratio $R={}^{13}C/^{12}C$ for single cells of algae and bacteria from SIMS measurements. For bacterial cells, the elliptical tool was used to select regions of interest (ROIs) in the ${}^{12}C{{}^{14}N}^{-}$. The isotope ratio for each cell was calculated by taking the mean value for the $100$ scans of SIMS measurements, from which the atomic fraction of ${}^{13}C$, i.e. $f= {}^{13}C/({}^{13}C+{}^{12}C)$ was calculated using

$f =\frac{R}{1+R}$. (S3)

During the SIMS analysis there were a few fields of view that contained region(s) of a size comparable to bacterial cells and with a relatively high atomic fraction of ${}^{13}C$. Points were considered outliers and not included in the calculation of the mean if they had an atomic fraction of ${}^{13}C$ greater than $f_{\max}= p_{2}+ 4\cdot\left( p_{2}-p_{1} \right)$, where $p_{1}$ and $p_{2}$ are the $25th$ and $75th$ percentile respectively. These regions might correspond to bacteria with a relatively high DIC uptake rate or could be the result of an experimental artefact, for example small amounts of sub-resolution organic matter debris or cross-contamination between samples. From confocal microscopy images, *C. reinhardtii* metE7 cells were measured to be about 5-10 µm in diameter and *M. japonicum* cells approximately 1 µm in length. Due to the relatively low spatial resolution of the instrument, it is possible that algal-derived smaller particles or other kinds of organic matter debris were included in the analyses of bacterial cells and could account for the small number of outliers. The rare occurrence of cross-contamination can occur during sample preparation or inside the SIMS instrument. Sputtering with the primary ion beam could cause material from one sample to be deposited on a neighbouring sample, or due to the close proximity of the first lens to the sample surface, material from one sample can land on the mechanical structure of the lens and subsequently be re-deposited onto a different sample (Deline 1983; McPhail, D. and Dowsett 2009). Future work could benefit from further consideration of this potential for cross-contamination, taking care to not arrange samples too tightly and to minimise the swapping between samples in the run sequence of SIMS analysis.

For algae, the SIMS results for highly labelled cells showed an inhomogeneous enrichment in${}^{13}C$, which meant that using only the ${}^{12}C{}^{14}N$ isotope signal to define the cells in the SIMS images could have led to biases. Therefore, in order to select algal cells in a way that was not biased towards a particular carbon isotope, a linear combination image was created by a simple addition of the two isotope counts $\left( 1\cdot{}^{12}C{}^{14}N + 1\cdot{}^{13}C{}^{14}N \right)$, which gives the total distribution of carbon across the area scanned. By comparing this with the fluorescence images, the ROIs corresponding to algal cells were selected. The isotope ratio $R$was calculated by taking the mean for the $100$ scans of each measurement, from which the atomic fraction of ${}^{13}C$ was calculated using equation (S3). For the preliminary experiment only one measurement of $100$ scans was completed, whereas for the final experiment $2-8$ repeated measurements for each algal cell was obtained.

## Depth analysis

The isotope content can be heterogeneously distributed within the cell and therefore a depth analysis was performed by obtaining repeated measurements of the same cells. SIMS is a destructive technique, meaning that through the process of measurement, as the primary ion beam scans across the sample, the cellular biomass is gradually degraded. For algal cells, the first measurement resulted in only partial degradation of the algal biomass. For three repeated measurements of the atomic fraction $f$ of ${}^{13}C$ for the same algal cells, the difference between the third and first measurements $\left( \Delta f=f_{3}-f_{1} \right)$ was calculated relative to the mean $\left( \bar{f}=\left( f_{1}+f_{2}+f_{3} \right)/3 \right)$. The results showed that for the majority of algal cells $f$ either increased or decreased for repeated measurements (Figure S2A), suggesting that the ${}^{13}C$-enrichment of algal cells was not homogeneous. In order to obtain a measurement that was representative of the whole cell, the mean of three repeated measurements was taken as the value for $f$ of an individual algal cell (with the exception of two cells for the $6 h$ sample of the pre-labelling culture of algae, for which only two repeated measurements were taken). For bacterial cells, most of the biomass was degraded after the first measurement (Figure S2B), therefore one measurement was sufficient for analysing the carbon isotope content of bacteria.

## Scattering effect for highly labelled algae

When the SIMS scan area contained a labelled algal cell, the $f$ values for bacterial cells in that area were both higher and more variable (Figure S2C). Proximity of algal and bacterial cells on the filter did not necessarily mean physical proximity during growth in the co-culture. Therefore the increase in $f$ for bacteria close to algae on the filter was not simply due to preferential access to DOC exudate. As the caesium ion beam is scanned across the sample, the cellular material is sputtered away to produce secondary ions. However, some of the algal biomass may not be captured as secondary ions and could instead be scattered on the filter in the region around the algal cell. This could explain the observed increase in the mean and standard deviation of the atomic fraction of ${}^{13}C$ for bacterial cells analysed in the same area as a labelled algal cell. As a result of this observation, only bacteria from scan areas that did not contain labelled algae were included in the analysis described in this work.

## Dilution effect - comparing bulk and single cell measurements

The sample preparation for SIMS analysis introduced unlabelled carbon into the cells during chemical fixation and nucleic acid staining, therefore the atomic fraction of ${}^{13}C$ was diluted. As established by (Musat et al. 2014), the relationship between the atomic fraction measured by SIMS $f_{SIMS}$ and the atomic fraction for the sample before chemical fixation and staining $f$ is

$f=f_{SIMS}+D \left( f_{SIMS}-f_{ch} \right)$, (S4)

where $D$ is the dilution factor and $f_{ch}$ is the atomic fraction of ${}^{13}C$ in the chemical fixative, and the nucleic acid stain, which were both assumed to be at natural abundance, i.e. $f_{ch}=0.0108$. The samples for EA-IRMS analysis, which was used for bulk analysis of the carbon isotope content, did not undergo any chemical fixation or staining. Therefore, the EA-IRMS results were assumed to give the true, undiluted value for $f$. To estimate the dilution factor $D$, equation (S4) was fitted to the SIMS and EA-IRMS data using $f=f_{IRMS}$ (Figure S2D and Table S3). For bacteria, the fit was carried out using data from only the axenic cultures. In subsequent analysis, to estimate the undiluted atomic fraction of ${}^{13}C$, the SIMS results were *dilution-corrected* using equation (S4) and the dilution factors $D_{a}=0.04$ for algal cells and $D_{b}=1.29$ for bacterial cells (Table S3). The dilution factor is higher for bacteria than for algae. This is likely to be because the bacterial cells were approximately 10 times smaller than the algal cells and therefore had a greater surface area to volume ratio, which could account for a greater uptake of the chemical fixative and nucleic acid stain.

# Nutrient-explicit model of the algal-bacterial co-culture

## Deriving atomic fractions from the nutrient-explicit model

The atomic fraction of ${}^{13}C$ is defined as the concentration of ${}^{13}C$ relative to the total carbon concentration, i.e. $f={}^{13}C/\left( {}^{13}C+{}^{12}C \right)$. Each of the different carbon components of the model (bacterial carbon, DOC, DIC etc.) can be considered as a separate carbon pool. For a general case, we consider the $n^{th}$ carbon pool $c_{n}$ with $r_{gain}$, the rate of carbon coming from the $c_{n-1}$ pool and $r_{loss}$, the rate of carbon going to $c_{n+1}$. This can be summarised as

$c_{n-1}\underset{\to}{r_{gain}}c_{n}\underset{\to}{r_{loss}}c_{n+1}$.

For example, when considering the whole DOC carbon pool,$c_{n}=c_{o}$, then $c_{n-1}=c_{a,p}$ and $c_{n+1}=c_{b}$ (the photosynthetic component of algal carbon and the bacterial carbon respectively), $r_{gain}=r_{e}$ (the rate of DOC exudation by algae) and $r_{loss}=X r_{u}$ (the rate of DOC uptake by bacteria), giving

$c_{a,p}\underset{\to}{r_{e}}c_{o}\underset{\to}{X r_{u}}c_{b}$.

The rate of change of the total carbon concentration for the $nth$ carbon pool is

$\frac{dc_{n}}{dt}=r_{gain}-r_{loss}$ (S5)

and the rate of change of the ${}^{13}C$ concentration for the $nth$ carbon pool is

$\frac{dc_{n}^{13}}{dt}=f_{n-1} r_{gain}-f_{n} r_{loss}$, (S6)

where $f_{n}$ is the atomic fraction of ${}^{13}C$ in the $n^{th}$ carbon pool and $f_{n-1}$ is the atomic fraction of ${}^{13}C$ in the $\left( n-1 \right)^{th}$ carbon pool. From equations (S5) and (S6), the rate of change of the atomic fraction of ${}^{13}C$ in the $n^{th}$ carbon pool can be derived using the quotient rule, giving

$\frac{df_{n}}{dt}=\left( f_{n-1}-f_{n} \right)\frac{r_{gain}}{c_{n}}$. (S7)

This general result for the isotope labelling dynamics of a carbon pool assumes that isotopic fractionation is negligible, which corresponds to the assumption that the difference between the nutrient rates for the different carbon isotopes is negligible compared to the overall labelling rates. This assumption leads to the conclusion that the rate of loss to the $c_{n+1}$ carbon pool for the ${}^{13}C$ and ${}^{12}C$ isotopes are equal, meaning that the rate of isotope labelling is explicitly independent of $r_{loss}$. However, $r_{loss}$ matters implicitly when comparing the growth and isotope labelling rates (i.e. substituting $r_{gain}$ in equation (S7) with $r_{gain}=\dot{c}_{n}+r_{loss}$ from equation (S6) gives $\dot{f}_{n}=\left( f_{n-1}-f_{n} \right)\left( \dot{c}_{n}+r_{loss} \right)/c_{n}$). If $r_{loss}$ is neglected, then $\dot{f}_{n}$ would overestimate $\dot{c}_{n}$, i.e. the labelling rate would overestimate the growth rate.

The algal carbon biomass of the model has two different internal carbon components, meaning that the carbon isotope labelling dynamics for algae does not follow the general case discussed above. In the model, the DOC produced by algae comes from only the photosynthetically active component of the algal biomass and therefore when $f_{a,s}$ is not equal to $f_{a,p}$, the rate of loss for ${}^{13}C$ and ${}^{12}C$ from the total algal biomass are not equal. The rate of algal carbon biomass growth is

$\frac{dc_{a}}{dt}=r_{p}-r_{e}=\frac{\dot{a}}{Y_{a,c}}$ (S8)

and the rate of change of the ${}^{13}C$ concentration for the algal carbon pool is

$\frac{dc_{a}^{13}}{dt}=f_{i} r_{p}-f_{a,p} r_{e}$, (S9)

from which the differential equation for the atomic fraction of ${}^{13}C$ in algae

$\frac{df_{a}}{dt}=\left( f_{i}-f_{a} \right)\mu_{a}\left( 1-\frac{a}{K_{a}} \right)\left( \frac{v}{K_{v}+v} \right)+\left( f_{i}-f_{a,p} \right)\left( 1-\phi_{s} \right) p_{c} Y_{a,c}$ (S10)

is obtained. In contrast to the general case outlined above, the rate of change of the atomic fraction of ${}^{13}C$ in algae includes a term for the rate of carbon loss.

Taking into consideration the ${}^{13}C$ isotope labelling dynamics in the general case for the $nth$ carbon pool and in the specific case for algae, the rate of change for the atomic fractions of ${}^{13}C$ in the co-culture are obtained, giving

$\frac{df_{a}}{dt}=\left( f_{i}-f_{a} \right) \mu_{a}\left( 1-\frac{a}{K_{a}} \right)\left( \frac{v}{K_{v}+v} \right)+\left( f_{i}-f_{a,p} \right)\left( 1-\phi_{s} \right) p_{c} Y_{a,c}$, (S11)

$\frac{df_{a,p}}{dt}=\left( f_{i}-f_{a,p} \right)\left[ \left( 1-\frac{a}{K_{a}} \right)\left( \frac{\mu_{a}}{1-\phi_{s}} \right)\left( \frac{v}{K_{v}+v} \right)+p_{c} Y_{a,c} \right]$, (S12)

$\frac{df_{a,s}}{dt}=\left( f_{a,p}-f_{a,s} \right) \mu_{a}\left( 1-\frac{a}{K_{a}} \right)\left( \frac{v}{K_{v}+v} \right)$, (S13)

$\frac{df_{b}}{dt}=\left( X f_{i}+\left( 1-X \right)f_{o}-f_{b} \right)\frac{\mu_{b}}{\eta}\left( \frac{c_{o}}{K_{c}+c_{o}} \right)$, (S14)

$\frac{df_{o}}{dt}=\left( f_{a,p}-f_{o} \right)\left( 1-\phi_{s} \right)\frac{p_{c} a}{c_{o}}$, (S15)

$\frac{df_{i}}{dt}=\left( f_{b}-f_{i} \right)\left( 1-\eta\left( 1-\frac{b}{K_{b}} \right) \right)\frac{\mu_{b} b}{Y_{b,c} \eta c_{i}}\left( \frac{c_{o}}{K_{c}+c_{o}} \right)$, (S16)

with $f_{a}$, $f_{a,p}$, $f_{a,s}$, $f_{b}$, $f_{o}$ and $f_{i}$ the atomic fractions of ${}^{13}C$ in the total algal carbon biomass, *photosynthetically-active* algal carbon, *stored* algal carbon, bacterial carbon, DOC and DIC respectively. This illustrates how a nutrient-explicit model can be used to derive equations for isotope labelling dynamics, allowing the model to make predictions that can be experimentally tested.

## Non-dimensional model

It is instructive to nondimensionalise the co-culture model in order to obtain the minimal set of parameters that characterise the general behaviour of the model. We used the non-dimensional model to perform a fixed point stability analysis and to obtain the parameter inequality constraints necessary for the parameter optimisations that ensure that the fixed point exists at positive values (see section below).

The algal and bacterial cell densities were nondimensionalised using their carrying capacities, that is $\hat{a}=a/K_{a}$ and $\hat{b}=b/K_{b}$ respectively. The B_12_ concentration was rescaled using the half-saturation concentration for algal growth, that is $\hat{v}=v/K_{v}$. All the carbon concentrations were rescaled using the half-saturation concentration for bacterial growth, that is $\hat{c}=c/K_{c}$. To nondimensionalise time the bacterial maximum growth rate was used, that is $\hat{t}=t \mu_{b}$. See Table S6 for the definitions of the non-dimensional parameters $\varepsilon$, $k_{a,v}$, $k_{a,c}$, $k_{b,c}$, $s_{v}$ and $s_{c}$. From these definitions the non-dimensional ODEs are

$\frac{d\hat{a}}{d\hat{t}}=\varepsilon\hat{a} \left( 1-\hat{a} \right)\left( \frac{\hat{v}}{1+\hat{v}} \right)$, $\frac{d\hat{b}}{d\hat{t}}=\hat{b} \left( 1-\hat{b} \right)\left( \frac{\hat{c}_{o}}{1+\hat{c}_{o}} \right)$, (S17)

$\frac{d\hat{c}_{o}}{d\hat{t}}=r_{e}-\left( 1-X \right)r_{u}$, $\frac{d\hat{c}_{i}}{d\hat{t}}=r_{r}-X r_{u}-r_{p}$, (S18)

$\frac{d\hat{v}}{d\hat{t}}=\varepsilon s_{v} \hat{b}-r_{v}$. (S19)

The non-dimensional carbon biomass conversion relations are

$\hat{c}_{a}=k_{a,c} \hat{a}$, $\hat{c}_{b}=k_{b,c}\hat{b}$, (S20)

$\hat{c}_{a,s}=\phi_{s} \hat{c}_{a}$, $\hat{c}_{a,p}=\hat{c}_{a}-\hat{c}_{a,s}$. (S21)

The non-dimensional metabolite rates are

$r_{s}=\phi_{s} k_{a,c}\frac{d\hat{a}}{d\hat{t}}$, $r_{e}=\left( 1-\phi_{s} \right) s_{c} \hat{a}$, (S22)

$r_{p}=k_{a,c}\frac{d\hat{a}}{d\hat{t}}+r_{e}$, $r_{v}=\varepsilon k_{a,v} \hat{a}\left( \frac{\hat{v}}{1+\hat{v}} \right)$, (S23)

$r_{u}=\frac{k_{b,c}\hat{b}}{\eta}\left( \frac{\hat{c}_{o}}{1+\hat{c}_{o}} \right)$, $r_{r}=\left( 1-\eta\left( 1-\hat{b} \right) \right)r_{u}$. (S24)

The non-dimensional ODEs for the atomic fractions are

$\frac{df_{a}}{d\hat{t}}=\left( f_{i}-f_{a} \right) \varepsilon\left( 1-\hat{a} \right)\left( \frac{\hat{v}}{1+\hat{v}} \right)+\left( f_{i}-f_{a,p} \right)\frac{\left( 1-\phi_{s} \right) s_{c}}{k_{a,c}}$, (S25)

$\frac{df_{a,p}}{d\hat{t}}=\left( f_{i}-f_{a,p} \right)\left[ \frac{\varepsilon\left( 1-\hat{a} \right)}{\left( 1-\phi_{s} \right)}\left( \frac{\hat{v}}{1+\hat{v}} \right)+\frac{s_{c}}{k_{a,c}} \right]$, (S26)

$\frac{df_{a,s}}{d\hat{t}}=\left( f_{a,p}-f_{a,s} \right) \varepsilon\left( 1-\hat{a} \right)\left( \frac{\hat{v}}{1+\hat{v}} \right)$, (S27)

$\frac{df_{b}}{d\hat{t}}=\left( X f_{i}+\left( 1-X \right)f_{o}-f_{b} \right)\frac{1}{\eta}\left( \frac{\hat{c}_{o}}{1+\hat{c}_{o}} \right)$, (S28)

$\frac{df_{o}}{d\hat{t}}=\left( f_{a,p}-f_{o} \right)\frac{\left( 1-\phi_{s} \right) s_{c} \hat{a}}{\hat{c}_{o}} ,$ (S29)

$\frac{df_{i}}{d\hat{t}}=\left( f_{b}-f_{i} \right)\frac{\left( 1-\eta\left( 1-\hat{b} \right) \right)k_{b,c} \hat{b}}{\eta\hat{c}_{i}}\left( \frac{\hat{c}_{o}}{1+\hat{c}_{o}} \right)$. (S30)

## Fixed point

In order for the model to describe a real system the fixed point must have positive values. Therefore, the equations defining the fixed point can be used to derive parameter constraints, which ensure that over time the model variables tend towards positive values.

For the co-culture model developed here, a non-zero fixed point exists where the algal cell density, bacterial cell density, DOC concentration and vitamin B_12_ concentration are all constant. The fixed point for the non-dimensional model is obtained by setting $\frac{d\hat{a}}{dt}=\frac{d\hat{b}}{dt}=\frac{d\hat{c}_{o}}{dt}=\frac{d\hat{v}}{dt}=0$, giving

$\hat{a}^{*}=1$, (S31)

$\hat{b}^{*}=1$, (S32)

$r_{e}=\left( 1-X \right)r_{u}$ $\to$ $\hat{c}_{o}^{*}=\frac{\left( 1-\phi_{s} \right)s_{c}}{\left( 1-X \right)\frac{k_{b,c}}{\eta} - \left( 1-\phi_{s} \right)s_{c}}$ , (S33)

$r_{v}=\varepsilon s_{v} \hat{b}^{*}$ $\to$ $\hat{v}^{*}=\frac{s_{v}}{k_{a,v}-s_{v}}$ . (S34)

At this fixed point, the algal and bacterial populations have reached carrying capacity, the rate of DOC production by algae is equal to the rate of DOC uptake by bacteria and the rate of B_12_ production by bacteria is equal to the rate of B_12_ uptake by algae. It is not relevant to consider the case where the DIC concentration is constant, because the model assumes that DIC is in excess and does not affect the rate of algal or bacterial growth.

In order for this fixed point to exist at positive values of $\hat{c}_{o}^{*}$ and $\hat{v}^{*}$, the parameters of the model must satisfy the inequality constraints

$\left( 1-X \right)\frac{k_{b,c}}{\eta}-\left( 1-\phi_{s} \right) s_{c}>0$, (S35)

$k_{a,v}-s_{v}>0$, (S36)

The isotope labelling dynamics reach a fixed point when all the atomic fractions of ${}^{13}C$are equal (i.e. $f_{i}^{*}=f_{o}^{*}=f_{a}^{*}=f_{a,p}^{*}=f_{a,s}^{*}=f_{b}^{*}=f^{*}$). This fixed point is defined as

$f^{*} =\frac{f_{i}\left( 0 \right) c_{i}\left( 0 \right)+f_{o}\left( 0 \right) c_{o}\left( 0 \right)+f_{a}\left( 0 \right) c_{a}\left( 0 \right)+f_{b}\left( 0 \right) c_{b}\left( 0 \right)}{c_{i}\left( 0 \right)+c_{o}\left( 0 \right)+c_{a}\left( 0 \right)+c_{b}\left( 0 \right)}$, (S37)

which can be intuitively understood as simply the weighted average of the initial atomic fractions of ${}^{13}C$ present in the system. This fixed point depends on the initial conditions, since it depends on the total amount of ${}^{13}C$ in the co-culture system.

Using the extended co-culture model equations in their non-dimensional form, the Jacobian matrix

$J=\left( \begin{matrix} \frac{\varepsilon\left( 1-2\hat{a} \right) \hat{v}}{1+\hat{v}} & 0 & 0 & \frac{\varepsilon\hat{a} \left( 1-\hat{a} \right)}{\left( 1+\hat{v} \right)^{2}} \\ 0 & \frac{\left( 1-2\hat{b} \right) \hat{c}_{o}}{1+\hat{c}_{o}} & \frac{\hat{b} \left( 1-\hat{b} \right)}{\left( 1+\hat{c}_{o} \right)^{2}} & 0 \\ s_{c}\left( 1-\phi_{s} \right) & -\frac{\left( 1-X \right) k_{b,c} \hat{c}_{o}}{\eta\left( 1+\hat{c}_{o} \right)} & -\frac{\left( 1-X \right) k_{b,c} \hat{b}}{\eta\left( 1+\hat{c}_{o} \right)^{2}} & 0 \\ -\frac{\varepsilon k_{a,v} \hat{v}}{1+\hat{v}} & \varepsilon s_{v} & 0 & -\frac{\varepsilon k_{a,v} \hat{a}}{\left( 1+\hat{v} \right)^{2}} \end{matrix} \right)$ (S38)

was obtained for the ordinary differential equations describing the rate of change of the algal cell density, bacterial cell density, DOC concentration and vitamin B_12_ concentration. The atomic fraction of ${}^{13}C$ is not included in this analysis because the fixed point $f^{*}$ in equation (S37) and the fixed point for $\hat{a}^{*}$, $\hat{b}^{*}$, ${\hat{c}_{o}}^{*}$ and $\hat{v}^{*}$ defined in equations (S31)-(S34) are independent. In order to determine the stability of the fixed point associated with the population sizes and nutrient concentrations, the Jacobian matrix was evaluated at the fixed point ($\hat{a}^{*}$, $\hat{b}^{*}$, ${\hat{c}_{o}}^{*}$, $\hat{v}^{*}$), giving

$J^{*}=\left( \begin{matrix} -x_{1} & 0 & 0 & 0 \\ 0 & -x_{2} & 0 & 0 \\ y_{1} & -y_{1} & -x_{3} & 0 \\ -y_{2} & y_{2} & 0 & -x_{4} \end{matrix} \right)$, (S39)

with $x_{1}=\frac{\varepsilon s_{v}}{k_{a,v}}$,

$x_{2}=\frac{\left( 1-\phi_{s} \right)s_{c}}{\left( 1-X \right)k_{b,c}/\eta}$,

$x_{3}=\frac{\left( 1-X \right)k_{b,c}}{\eta}\left[ 1-\frac{\left( 1-\phi_{s} \right)s_{c}}{\left( 1-X \right)k_{b,c}/\eta} \right]^{2}$,

$x_{4}=\varepsilon k_{a,v}\left[ 1-\frac{s_{v}}{k_{a,v}} \right]^{2}$,

$y_{1}=s_{c}\left( 1-\phi_{s} \right)$,

$y_{2}=\varepsilon s_{v}$.

The four eigenvalues of $J^{*}$ are

$\lambda=-x_{1}, -x_{2}, -x_{3} or -x_{4}$, (S40)

which are all negative because $x_{1}$, $x_{2}$, $x_{3}$ and $x_{4}$ are strictly positive (equations (S39)). Therefore the fixed point is asymptotically stable, meaning that any small perturbation will converge back to the fixed point (Terrell 2009).

# Estimating model parameters and solving the model equations

To reduce the number of free parameters, the majority of parameter values were constrained to match values obtained independently from axenic cultures and additional co-culture experiments. The majority of the model parameters were determined using a simplified version of the co-culture model (i.e. with $\phi_{s}=0$, $\eta^{'}=1$ and $X=0$) to run a global fit of three independent co-culture experiments, which measured colony forming units, particle counts and B_12_ concentrations for a co-culture between *C. reinhardtii* metE7 and *M. japonicum*, see below for details. The algal and bacterial carbon yields were estimated from dry mass measurements and EA-IRMS analysis, see above for details. The remaining parameters were obtained from fitting the model to the stable isotope experiments in this work, see below for details. The full set of model parameters and initial conditions for *C. reinhardtii* metE7 and *M. japonicum* grown both axenically and in co-culture are given in Table 1, and the non-dimensional parameters are given in Table S6. Table S7 defines the culture specific parameters and initial conditions for the four axenic cultures of bacteria grown with different concentrations of glycerol. Table S8 compares the results of two parameter optimisations, one with $f_{o}\left( 0 \right)=0.64$ and the other with $f_{o}\left( 0 \right)$ included as a free initial condition. The Matlab ordinary differential equation solver *ode45* was used to numerically solve the model equations.

## Parameter optimisation for a simplified co-culture model

Several of the model parameters were estimated for a co-culture between *C. reinhardtii* metE7 and *M. japonicum* by a global fit of a simplified co-culture model (i.e. with $\phi_{s}=0$, $\eta'=1$ and $X=0$) to experimental results for three independent co-culture experiments using a basin-hopping algorithm (Figure S5). The co-cultures were grown in Tris-minimal media, $25^{\circ}C$, $16:8 h$ light:dark cycle and for $42$, $16$ and $24$ days for experiments $1$, $2$ and $3$ respectively. All three experiments measured colony forming units of *M. japonicum* and a bioassay, as described in (Bunbury et al. 2020), was used to measure the total vitamin B_12_ concentration. For experiment $1$ colony forming units of *C. reinhardtii* metE7 were used for the fit, whereas for experiments $2$ and $3$ algal counts in terms of particles $>3 \mu m$ on the Coulter counter were used. Experiments$1$, $2$ and $3$ include $8$, $4$ and $5$ replicates respectively.

## Estimating $K_{b}$ for axenic bacteria

To estimate the carrying capacity $K_{b}$ for axenic cultures of *M. japonicum*, the logistic growth equation $b=K_{b}/(1+M e^{-r t})$, with positive constant $M$ and $r$, was fit to data taken from (Kazamia et al. 2012) for *M. japonicum* grown axenically with $0.1 \%$ glycerol. The result is given in Figure S6.

## Parameter optimisations using SIMS results

Parameter optimisations were performed by fitting the nutrient-explicit co-culture model defined above to the population growth and SIMS ${}^{13}C$-enrichment results. Growth was measured using viable counts and the atomic fractions of ${}^{13}C$ used were the mean values of the dilution-corrected, single cell measurements obtained using SIMS for each time-point. The Matlab ordinary differential equation solver *ode45* was used to numerically solve the model equations. The parameter optimisations were performed as a global search of the parameter space in order to obtain the best estimate for the set of parameters that minimise the deviation of the model from experiment and that lie within the fitting intervals (Table S5) and inequality constraints (equations (S35) and (S36)). Global parameter optimisations were performed using the *GlobalSearch* and *createOptimProblem* functions in Matlab's global optimisation toolbox, with *fmincon* as the solver for each minimisation. All default settings were used except for the *StartPointsToRun* property of the *GlobalSearch* function, which was selected to run with the *bounds-ineqs* option, meaning that all starting points for the minimisations had to lie within the fitting intervals and satisfy the inequality constraints. In order to minimise the number of free parameters, the parameters obtained for the fit of a simplified co-culture model (as outlined above) were used in the parameter optimisations.

## Parameter optimisation for the pre-labelling, axenic culture of algae.

From the non-dimensional co-culture model defined above, an axenic culture of algae can be modelled by setting the initial bacterial concentration to zero (i.e. $b\left( 0 \right)=0$). Using the experimental data obtained for the pre-labelling, axenic culture of *C. reinhardtii* metE7, the objective function minimised by the parameter optimisation was

$r^{2}\left( a, f_{a} \right)=\sum_{t} \left( \frac{a_{model}\left( t \right)-a_{exp}\left( t \right)}{a_{exp}\left( t \right)} \right)^{2}+\left( \frac{f_{a, model}\left( t \right)-f_{a, exp}\left( t \right)}{f_{a,exp}\left( t \right)} \right)^{2}$, (S41)

which gives a measure for the deviation of the model from the experiment for both the algal cell density $a$ and atomic fraction of ${}^{13}C$ for the algal biomass $f_{a}$. In equation (S41) the sum corresponds to the sum over all time-points in the experiment, the subscript $model$ refers to the value obtained from the model and the subscript $exp$ refers to the value measured experimentally.

*Free parameters and initial conditions*. The free parameters were $s_{c}$ and $\phi_{s}$. All other parameter values used were as defined in Table 1 and Supplementary Table S6. For the experiment, it was assumed that initially there was no DOC in the media, the DIC was in excess and the algae were initially unlabelled, therefore $\hat{c}_{o}\left( 0 \right)=0$, $\hat{c}_{i}\left( 0 \right)=5$, $f_{a}\left( 0 \right)=0.0108$ and $f_{o}\left( 0 \right)=0.0108$. No reliable measurement for the initial algal cell density was obtained and although the initial B_12_ concentration was $7.5\times{10}^{-14} mol {mL}^{-1}$ (i.e. $100 ng L^{-1}$), the model for algal growth neglects the internal B_12_ recycling dynamics and so the B_12_ concentrations in the model do not necessarily correspond to the quantitative values of the experiment, therefore the initial algal cell density and B_12_ concentration were kept free. Although the $NaH{}^{13}CO_{3}$ used for the stable isotope labelling cultures had $98 atm\%{}^{13}C$, due to the equilibria between different forms of inorganic carbon, the actual atomic fraction of ${}^{13}C$ for the DIC assimilated by the algae was unknown, therefore the initial condition $f_{i}\left( 0 \right)$ was also kept free.

## Parameter optimisation for axenic bacteria

An axenic culture of bacteria can be modelled using the co-culture model defined above and setting the initial algal cell density to zero (i.e. $a\left( 0 \right)=0$). A global parameter optimisation was performed for axenic bacteria using the experimental results of four cultures of *M. japonicum*, each grown with a different concentration of glycerol ($0.1 \%$, $0.01 \%$, $0.001 \%$ and no glycerol). The objective function minimised by the global parameter optimisation was

$r^{2}\left( b, f_{b} \right)=\sum_{all cultures} \sum_{t} \left( \frac{b_{model}\left( t \right)-b_{exp}\left( t \right)}{b_{exp}\left( t \right)} \right)^{2}+\left( \frac{f_{b,model}\left( t \right)-f_{b,exp}\left( t \right)}{f_{b,exp}\left( t \right)} \right)^{2}$, (S42)

with the sum over $all cultures$ indicating that the aim was to minimise the difference between the model and the experimental results for the bacterial cell density $b$ and the atomic fraction of ${}^{13}C$for bacteria $f_{b}$ for all four axenic cultures simultaneously.

***Free parameters***. The model parameters for axenic bacteria were considered as global parameters, with the exception of $\eta$ and $X$ that could have values specific to the different cultures. The carrying capacity, maximum growth rate and carbon uptake parameter for bacteria ($K_{b}$, $\mu_{b}$ and $k_{b,c}$ respectively) obtained for *M. japonicum* in co-culture with *C. reinhardtii* metE7 might not be the same as for *M. japonicum* grown in axenic cultures in which bacteria are grown with glycerol as their organic carbon source. Therefore $K_{b}$ was determined as described above, and $\mu_{b}$ and $K_{c}$ were kept as free global parameters. Using $Y_{b,c}=5\times{10}^{14} cells molC^{-1}$ obtained from dry mass measurements and EA-IRMS results (as described above), the value for the carbon uptake parameter $k_{b,c}$ was updated throughout the parameter optimisation as $K_{c}$ changed, according to the parameter definition $k_{b,c}=K_{b}/(Y_{b,c} K_{c})$.

***Initial conditions***. It was assumed that in the experiments the DIC was in excess and the atomic fraction of ${}^{13}C$in the DIC was taken as the estimate obtained from the parameter optimisation for axenic algae, meaning $\hat{c}_{i}\left( 0 \right)=5$ and $f_{i}\left( 0 \right)=0.65$. Initially, there was no B_12_ in the media, the bacteria were at natural abundance and the glycerol was unlabelled; therefore $\hat{v}\left( 0 \right)=0$, $f_{b}\left( 0 \right)=0.0108$ and $f_{o}\left( 0 \right)=0.0108$. The initial DOC concentrations were calculated for $0.1 \%$, $0.01 \%$ and $0.001 \%$ glycerol concentrations to be $4\times{10}^{-5}$, $4\times{10}^{-6}$ and $4\times{10}^{-7} molC mL^{-1}$ respectively, using the molar mass of glycerol, $92.09 g mol^{-1}$, and its density, $1.26 g mol^{-1}$. Results for axenic bacteria grown without glycerol showed a small amount of bacterial growth (Figure 2), which could be due to internal carbon carried forward from the pre-culture or slow growth on other organic carbon sources, e.g. Tris buffer, present in the media. In order to account for this observation, $c_{o}\left( 0 \right)$ for the `no glycerol' culture was kept free, but was constrained to be less than $4\times{10}^{-7} molC mL^{-1}$ (i.e. $0.001 \%$ glycerol). No reliable measurement for the initial bacterial cell density was obtained experimentally, therefore $b\left( 0 \right)$ for each culture was also kept free in the global parameter optimisation.

## Parameter optimisation for the co-culture

The objective function of the parameter optimisations for the co-culture was

$r^{2}\left( a, b, f_{a}, f_{b} \right)=\sum_{t} \left( \frac{a_{model}\left( t \right)-a_{exp}\left( t \right)}{a_{exp}\left( t \right)} \right)^{2}+\left( \frac{b_{model}\left( t \right)-b_{exp}\left( t \right)}{b_{exp}\left( t \right)} \right)^{2}$ $+\left( \frac{f_{a, model}\left( t \right)-f_{a, exp}\left( t \right)}{f_{a,exp}\left( t \right)} \right)^{2}+\left( \frac{f_{b,model}\left( t \right)-f_{b,exp}\left( t \right)}{f_{b,exp}\left( t \right)} \right)^{2}$, (S43)

which gives a measure for the deviation of the model from the experiment for both the cell densities ($a$ and $b$ for algae and bacteria respectively) and the atomic fractions of ${}^{13}C$ ($f_{a}$ and $f_{b}$ for algae and bacteria respectively).

***Estimating*** $\boldsymbol{\phi}_{\boldsymbol{s}}$***,*** $\boldsymbol{\eta}$ ***and*** $\boldsymbol{X}$. The result $\phi_{s}=0.9$, obtained from the parameter optimisation for axenic algae, was carried forward for the co-culture model. The results for the axenic bacteria suggested that for a higher initial glycerol concentration, and therefore a higher exponential growth rate, the value for the DIC uptake parameter $X$ increases and the value for the bacterial growth efficiency $\eta$ decreases. These trends were used to estimate $X$ and $\eta$ for the co-culture. For axenic bacteria, the initial glycerol concentration $c_{o}\left( 0 \right)$ was used to estimate the exponential growth rate $\mu_{B}=\mu_{b} c_{o}\left( 0 \right)/\left( c_{o}\left( 0 \right)+K_{c} \right)$. An exponential growth rate fit for bacteria in the co-culture gave estimates for the initial bacterial cell density $b\left( 0 \right)=1.2\times{10}^{7}\pm1.5\times{10}^{5} cfu mL^{-1}$ and the exponential growth rate $\mu_{B}=0.022\pm0.005 h^{-1}$ (Figure S7). A linear fit for $X$ against $\text{ln}\left( \mu_{B} \right)$ (Figure S8A) was used to obtain the estimate $X=0.015\pm0.001$ for bacteria in the co-culture. A linear fit for $\eta$ against $\text{ln}\left( \mu_{B} \right)$ (Figure S8B) was used to obtain the estimate $\eta=0.51\pm0.21$ for bacteria in the co-culture.

***Free parameters and initial conditions***. The majority of the model parameters were fixed with values as defined in Table 1 and Supplementary Table S6, apart from $s_{c}$, which was included as a free parameter. The initial conditions of the co-culture meant that DIC was in excess and the B_12_ concentration was assumed to be zero (because bacteria were washed thoroughly prior to establishing the co-culture and B_12_ was assumed to have been fully depleted in the pre-labelling culture of algae because it was inoculated with only $7.5\times{10}^{-14} mol {mL}^{-1}$B_12_), therefore $\hat{c}_{i}\left( 0 \right)=5$ and $\hat{v}\left( 0 \right)=0$. For the initial atomic fraction of ${}^{13}C$ in the DIC, the estimate obtained from the parameter optimisation for axenic algae was used, i.e. $f_{i}\left( 0 \right)=0.65$. The co-culture was inoculated with pre-labelled algae, therefore the $48 h$ time-point of the pre-labelling culture was used to estimate the initial atomic fractions of ${}^{13}C$ in the algae and DOC. Using the model fit results for the axenic algae given in Table 1, estimates for the initial conditions $f_{a}\left( 0 \right)=0.59$, $f_{a,p}\left( 0 \right)=0.65$ and $f_{o}\left( 0 \right)=0.64$ for the co-culture were obtained. The bacteria started the co-culture at natural abundance and so $f_{b}\left( 0 \right)=0.0108$. The initial conditions that remained free during the parameter optimisations were $\hat{a}\left( 0 \right)$, $\hat{b}\left( 0 \right)$ and $\hat{c}_{o}\left( 0 \right)$, with $f_{o}\left( 0 \right)$ also included as a free initial condition for fit 2.

II. Supplementary Results

# Pre-labelling algae in an axenic culture

The B_12_ dependent *C. reinhardtii* metE7 was grown axenically for $48 h$ in media containing $5 mM$ $NaH{}^{13}CO_{3}$, which provided a ${}^{13}C$-enriched inorganic carbon source for photosynthesis. For each time-point, SIMS images (Figure S9A) were used to obtain measurements of the atomic fraction of ${}^{13}C$ in individual algal cells (Figure S9B). The mean $f_{a}$ was then calculated (Figure S9C), excluding the cells close to natural abundance, which are highlighted in red in S9B, because they were assumed to be inactive and not contribute to the carbon dynamics of the culture. The value for $f_{a}$ increased throughout the culture, indicating that *C. reinhardtii* metE7 used the ${}^{13}C$-enriched DIC for photosynthesis and growth (Figure S9C-D). The rate of ${}^{13}C$-enrichment decreased as the culture progressed, with $f_{a}$ beginning to plateau (Figure S9C). It is likely that the labelling rate decreases when $f_{a}$ approaches the value of $f_{i}$, meaning that $f_{a}$ and $f_{i}$ reach an equilibrium. Although the $NaH{}^{13}CO_{3}$ used had an atomic fraction of ${}^{13}C$ of $0.98$, due to the equilibria between different forms of DIC and atmospheric carbon dioxide, the actual atomic fraction of ${}^{13}C$ for the DIC assimilated by the algae is unknown. The model achieved a good fit to the experimental data for the axenic culture of algae (Figure S9 and Table 1) and estimated the initial atomic fraction of ${}^{13}C$ in the DIC to be $f_{i}\left( 0 \right)=0.65$. This value for $f_{i}\left( 0 \right)$ was used in the parameter optimisations for the axenic cultures of bacteria and the co-culture. In the model, as algae become labelled, the DOC they exude also becomes labelled. Using the parameter optimisation results given in Table 1, it was found that after 48 hours of the pre-labelling culture of algae, $f_{a}=0.59$, $f_{a,p}=0.65$ and $f_{o}=0.64$. These values were used as initial conditions for the model fit of the co-culture.

# Comparing growth rate and net carbon assimilation rate

The data we have acquired from SIMS provides the first quantitative measurement of carbon dynamics in a mutualistic algal-bacterial co-culture as a function of time. It is instructive to use the data to infer the carbon assimilation rate at each time point of our SIMS data. Two quantitative approaches are typically used to estimate net nutrient assimilation rates from isotope labelling experiments. Firstly, a method derived by (Montoya et al. 1996) and used more recently to quantify the carbon and nitrogen fixation rates of cyanobacteria (Foster et al. 2013; Eichner et al. 2017), defines the net carbon assimilated as the amount of carbon assimilated relative to the total carbon content in the organism. From this definition, the net carbon assimilation rate, in units $h^{-1}$, is given by

$v_{net}=\frac{1}{t}\left( \frac{f\left( t \right)-f_{0}}{f_{s}-f_{0}} \right)$, (S44)

with $f_{0}$ and $f\left( t \right)$ the atomic fraction of ${}^{13}C$ in the organism at time zero and at time $t$ respectively, and $f_{s}$ the atomic fraction of ${}^{13}C$ in the ${}^{13}C$-enriched source (Montoya et al. 1996). An alternative approach, established by (Popa et al. 2007) and recently used by (Arandia-Gorostidi et al. 2016) to study the effect of temperature and physical attachment on the carbon and nitrogen fluxes between phytoplankton and heterotrophic bacteria, defines the net carbon assimilation $Fx_{net}$ as the fraction of carbon in the sampled organism taken up from the labelled source relative to the fraction of carbon in the sampled organism remaining from the initial carbon content of the organism at time $t$, giving

$Fx_{net}=\frac{f\left( t \right)-f_{0}}{f_{s}-f\left( t \right)}$, (S45)

from which the net carbon assimilation rate is calculated as $Fx_{net}/t$, which has units $h^{-1}$. Note that in (Popa et al. 2007) although equation (6) omits a “-1” in the last term of the denominator, the correct equation for $Fx_{net}$ is used for the analysis (P. Weber, personal communication and (Dekas et al. 2019)). In contrast to $v_{net}$ (equation (S44)), $Fx_{net}$ does not rely on the assumption that there is no carbon loss from the organism, meaning that it can be used more generally and is more suitable for longer time frames. Therefore, $Fx_{net}$ is used here. However, both methods assume that carbon assimilation is linear in time and therefore are not suitable for complex carbon dynamics. It is also important to note that in order to obtain a true ‘cellular carbon-assimilation rate’ with units of${molC h}^{-1}$, $v_{net}$ or $Fx_{net}/t$ would need to be multiplied by the cellular carbon content. For comparison with the exponential growth rate, here we use the assimilation rate in units of $h^{-1}$ as defined by $Fx_{net}/t$.

Exponential growth of a bacterial population is given by the equation

$b = b\left( 0 \right)\exp\left( \mu_{B} t \right)$, (S46)

with $b$ and $b\left( 0 \right)$ the bacterial population size at time $t$ and at time zero respectively, and $\mu_{B}$ the exponential growth rate. To relate population growth to the bacterial carbon biomass, it is assumed that

$c_{b}=\frac{b}{Y_{b,c}}$, (S47)

with $c_{b}$ the bacterial biomass in $molC mL^{-1}$ and $Y_{b,c}$ a constant that defines the bacterial carbon yield in $cells molC^{-1}$. Substituting equation (S46) into equation (S47) gives an exponential growth equation for the bacterial carbon biomass

$c_{b}=c_{b}\left( 0 \right)\exp\left( \mu_{B} t \right)$, (S48)

with $c_{b}\left( 0 \right)$ the initial bacterial carbon biomass concentration. The change in carbon biomass concentration is defined as

$\Delta c_{b}=c_{b}-c_{b}\left( 0 \right)$. (S49)

Using equation (S48), equation (S49) can be rewritten as

$\Delta c_{b}=c_{b}\left( 0 \right)\left( \exp(\mu_{B} t \right)-1)$, (S50)

which can be approximated for short time periods, assuming $\mu_{B} t\ll1$, as

$\Delta c_{b}\approx c_{b}\left( 0 \right) \mu_{B} t$. (S51)

Rearranging, gives an expression for the exponential growth rate accounted for by carbon assimilation

$\mu_{B}=\frac{\Delta c_{b}}{t c_{b}\left( 0 \right)}$. (S52)

If bacteria loose carbon from their biomass due to respiration and take up carbon for growth and as an energy source, the net change in carbon biomass concentration can be defined as

$\Delta c_{b}=\Delta c^{+}-\Delta c^{-}$, (S53)

with $\Delta c^{+}$ the concentration of carbon biomass taken up and $\Delta c^{-}$ the concentration of carbon biomass lost from bacterial biomass. The fraction of carbon in the sampled bacteria taken up from the labelled carbon source can be approximated as

$F_{s}=\Delta c^{+}/c_{b}$. (S54)

The fraction of carbon in the sampled bacteria from the initial carbon content can be approximated as

$F_{i}=\frac{c_{b}\left( 0 \right)-\Delta c^{-}}{c_{b}}$. (S55)

According to the definition of net carbon assimilation $Fx_{net}$ (Popa et al. 2007) and using equations (S54) and (S55), the net carbon assimilation can be approximated as

$Fx_{net}=\frac{\Delta c^{+}}{c_{b}\left( 0 \right)-\Delta c^{-}}$. (S56)

In order to compare net carbon assimilation with growth rate it is assumed that $\Delta c^{-}\ll\Delta c^{+}$, such that $\Delta c_{b}\approx\Delta c^{+}$, which gives

$Fx_{net}\approx\frac{\Delta c_{b}}{c_{b}\left( 0 \right)}$. (S57)

Therefore $\Delta c_{b}\approx Fx_{net} c_{b}\left( 0 \right)$, which can be substituted into equation (S52) to give

$\mu_{B}\approx\frac{Fx_{net}}{t}$. (S58)

In summary, the above discussion shows that $Fx_{net}/t$ is a reasonable approximation to the population growth rate when $t\ll1/\mu_{B}$, $\Delta c^{-}\ll\Delta c^{+}$ and a linear relationship between population size and carbon biomass is assumed.

A fit of equation (S46) to the viable count measurements of co-cultured bacteria gave $b\left( 0 \right)=1.2\pm0.01\times{10}^{7}cfu mL^{-1}$ and $\mu_{B}=0.022\pm0.005 h^{-1}$, and for the axenic culture grown without glycerol the fit result was $b\left( 0 \right)=1.5\pm0.01\times{10}^{7} cfu mL^{-1}$ and $\mu_{B}=0.012\pm0.004 h^{-1}$ (Figure S7). Comparing the exponential growth rate for bacteria in the co-culture with the mean net carbon assimilation rate for the $6 h$ time-point, i.e. $Fx_{net}/t=0.0014 h^{-1}$ (using equation (S45)), implies that carbon assimilation of algal photosynthate accounts for approximately $6 \%$ of bacterial population growth in the co-culture. The growth rate of axenic bacteria grown without an organic carbon source as a percentage of the growth rate of bacteria in the co-culture was estimated to be $55 \%$. These comparisons between growth and carbon assimilation rates suggest that the uptake of organic carbon produced by algae was responsible for only some of the observed bacterial growth in the co-culture, with a significant contribution to bacterial growth from internal carbon storage carried forward from the pre-cultured bacteria.

# Comparing SIMS results of two independent experiments

A preliminary experiment for the SIMS analysis was performed for a pre-labelling culture of axenic algae, a labelled co-culture and an axenic culture of bacteria with $0.1 \%$ glycerol. The results from the preliminary SIMS experiment show the same trends in the isotope labelling dynamics as those observed for the final SIMS experiment (Figure S10). This illustrates the repeatability of the measurements obtained. The unlabelled control cultures for axenic algae and a co-culture were included in the preliminary experiments and showed the expected result of natural abundance.

# Supplementary references

Arandia-Gorostidi, N., Weber, P.K., Alonso-Sáez, L., Morán, X.A.G., and Mayali, X. (2016). Elevated Temperature Increases Carbon and Nitrogen Fluxes between Phytoplankton and Heterotrophic Bacteria through Physical Attachment. *The ISME Journal* 11 (3): 641.

Bunbury, F., Helliwell, K.E., Mehrshahi, P., Davey, M.P., Salmon, D.L., Holzer, A., et al. (2020). Physiological and Molecular Responses of a Newly Evolved Auxotroph of Chlamydomonas to B12 Deprivation. *Plant Physiology*, pp.01375.2019.

Dekas, A.E., Parada, A.E., Mayali, X., Fuhrman, J.A., Wollard, J., Weber, P.K., and Pett-Ridge, J. (2019). Characterizing Chemoautotrophy and Heterotrophy in Marine Archaea and Bacteria With Single-Cell Multi-Isotope NanoSIP. *Frontiers in Microbiology* 10 (December).

Deline, V.R. (1983). Instrumental Cross-Contamination in the Cameca IMS-3F Secondary Ion Microscope. *Nuclear Instruments and Methods in Physics Research* 218: 316–18.

Eichner, M.J., Klawonn, I., Wilson, S.T., Littmann, S., Whitehouse, M.J., Church, M.J., et al. (2017). Chemical Microenvironments and Single-Cell Carbon and Nitrogen Uptake in Field-Collected Colonies of Trichodesmium under Different PCO2. *Nature Publishing Group* 11 (6): 1305–17.

Foster, R.A., Sztejrenszus, S., and Kuypers, M.M.M. (2013). Measuring Carbon and N2 Fixation in Field Populations of Colonial and Free-Living Unicellular Cyanobacteria Using Nanometer-Scale Secondary Ion Mass Spectrometry. *Journal of Phycology* 516: 502–16.

Kazamia, E., Czesnick, H., Nguyen, T.T. Van, Croft, M.T., Sherwood, E., Sasso, S., et al. (2012). Mutualistic Interactions between Vitamin B12-Dependent Algae and Heterotrophic Bacteria Exhibit Regulation. *Environmental Microbiology* 14 (6): 1466–76.

Kropat, J., Hong-Hermesdorf, A., Casero, D., Ent, P., Castruita, M., Pellegrini, M., et al. (2011). A Revised Mineral Nutrient Supplement Increases Biomass and Growth Rate in Chlamydomonas Reinhardtii. *The Plant Journal* 66 (5): 770–80.

McPhail, D. and Dowsett, M. (2009). Dynamic SIMS. In *Surface Analysis - The Principal Techniques: Second Edition*, 207–68. John Wiley and Sons.

Montoya, J.P., Voss, M., Kahler, P., and Capone, D.G. (1996). A Simple, High-Precision, High-Sensitivity Tracer Assay for N2 Fixation. *Applied and Environmental Microbiology* 62 (3): 986–93.

Musat, N., Stryhanyuk, H., Bombach, P., Adrian, L., Audinot, J.N., and Richnow, H.H. (2014). The Effect of FISH and CARD-FISH on the Isotopic Composition of 13C- and 15N-Labeled Pseudomonas Putida Cells Measured by NanoSIMS. *Systematic and Applied Microbiology* 37 (4): 267–76.

Peaudecerf, F.J., Bunbury, F., Bhardwaj, V., Bees, M.A., Smith, A.G., Goldstein, R.E., and Croze, O.A. (2018). Microbial Mutualism at a Distance: The Role of Geometry in Diffusive Exchanges. *Physical Review E* 97: 022411.

Popa, R., Weber, P.K., Pett-Ridge, J., Finzi, J.A., Fallon, S.J., Hutcheon, I.D., et al. (2007). Carbon and Nitrogen Fixation and Metabolite Exchange in and between Individual Cells of Anabaena Oscillarioides. *The ISME Journal* 1 (4): 354–60.

Terrell, W.J. (2009). *Stability and Stabilization: An Introduction*. Princeton University Press.

Watrous, J.D., and Dorrestein, P.C. (2011). Imaging Mass Spectrometry in Microbiology. *Nature Reviews. Microbiology* 9 (9): 683–94.

1. Current address: Living Systems Institute, University of Exeter, United Kingdom [↑](#footnote-ref-1)
2. Current address: Institute of Environmental Engineering, Department of Civil, Environmental and Geomatic Engineering, ETH Zürich, Switzerland [↑](#footnote-ref-2)
3. Current address: Carnegie Institution for Science, Stanford, United States [↑](#footnote-ref-3)
